# The genetic variability, phylogeny and functional significance of E6, E7 and LCR in human papillomavirus type 52 isolates in Sichuan, China

**DOI:** 10.1186/s12985-021-01565-5

**Published:** 2021-05-03

**Authors:** Zhilin Song, Yanru Cui, Qiufu Li, Junhang Deng, Xianping Ding, Jiaoyu He, Yiran Liu, Zhuang Ju, Liyuan Fang

**Affiliations:** 1 Key Laboratory of Bio-Resource and Eco-Environment of Ministry of Education, College of Life Sciences, Sichuan University, Chengdu, 610065 Sichuan People’s Republic of China; 2 Bio-Resource Research and Utilization Joint Key Laboratory of Sichuan and Chongqing, Chongqing, People’s Republic of China

**Keywords:** Human papillomavirus, E6, E7, LCR, Polymorphism, Phylogeny, Antigen epitopes, Transcription factor binding site

## Abstract

**Background:**

Variations in human papillomavirus (HPV) E6 and E7 have been shown to be closely related to the persistence of the virus and the occurrence and development of cervical cancer. Long control region (LCR) of HPV has been shown multiple functions on regulating viral transcription. In recent years, there have been reports on E6/E7/LCR of HPV-16 and HPV-58, but there are few studies on HPV-52, especially for LCR. In this study, we focused on gene polymorphism of the HPV-52 E6/E7/LCR sequences, assessed the effects of variations on the immune recognition of viral E6 and E7 antigens, predicted the effect of LCR variations on transcription factor binding sites and provided more basic date for further study of E6/E7/LCR in Chengdu, China.

**Methods:**

LCR/E6/E7 of the HPV-52 were amplified and sequenced to do polymorphic and phylogenetic analysis. Sequences were aligned with the reference sequence by MEGA 7.0 to identify SNP. A neighbor-joining phylogenetic tree was constructed by MEGA 7.0, followed by the secondary structure prediction of the related proteins using PSIPRED 4.0. The selection pressure of E6 and E7 coding regions were estimated by Bayes empirical Bayes analysis of PAML 4.9. The HLA class-I and II binding peptides were predicted by the Immune Epitope Database server. The B cell epitopes were predicted by ABCpred server. Transcription factor binding sites in LCR were predicted by JASPAR database.

**Results:**

50 SNP sites (6 in E6, 10 in E7, 34 in LCR) were found. From the most variable to the least variable, the nucleotide variations were LCR > E7 > E6. Two deletions were found between the nucleotide sites 7387–7391 (TTATG) and 7698–7700 (CTT) in all samples. A deletion was found between the nucleotide sites 7287–7288 (TG) in 97.56% (40/41) of the samples. The combinations of all the SNP sites and deletions resulted in 12 unique sequences. As shown in the neighbor-joining phylogenetic tree, except for one belonging to sub-lineage C2, others sequences clustered into sub-lineage B2. No positive selection was observed in E6 and E7. 8 non-synonymous amino acid substitutions (including E3Q and K93R in the E6, and T37I, S52D, Y59D, H61Y, D64N and L99R in the E7) were potential affecting multiple putative epitopes for both CD4+ and CD8+ T-cells and B-cells. A7168G was the most variable site (100%) and the binding sites for transcription factor VAX1 in LCR. In addition, the prediction results showed that LCR had the high probability binding sites for transcription factors SOX9, FOS, RAX, HOXA5, VAX1 and SRY.

**Conclusion:**

This study provides basic data for understanding the relation among E6/E7/LCR mutations, lineages and carcinogenesis. Furthermore, it provides an insight into the intrinsic geographical relatedness and biological differences of the HPV-52 variants, and contributes to further research on the HPV-52 therapeutic vaccine development.

**Supplementary Information:**

The online version contains supplementary material available at 10.1186/s12985-021-01565-5.

## Background

According to statistics, about 1.4 million women worldwide suffer from cervical cancer, which is the second most common cancer among women in the world and the main cause of cancer death in some developing countries [1–3]. Cervical cancer not only causes a serious threat to the health of women all over the world, but also causes a very high burden to social economy. Strong epidemiological and molecular evidence accumulated over the past few decades has confirmed the close connection between the persistent infection of high-risk human papillomavirus and the development of cervical cancer, almost all cervical cancer biopsies have found one or more high-risk human papillomavirus [4, 5].

Human papillomavirus (HPV) is a capsid-enclosed small circular double-stranded type of DNA virus of approximately 8 kb in size [6]. It can specifically infect human epithelium and mucosa, causing a variety of diseases, and is a large group of virus, which consists of more than 250 different types [7]. According to its pathogenicity, HPV types are classified into high risk and low risk [8]. HPV-16, 18, 31, 33, 35, 39, 45, 51, 52, 53, 56, 58, 59, 66, 68, 73 and 82 are the common high-risk types and have been proven to cause more than 96% of cervical cancers; among low-risk HPV types, HPV-6 and HPV-11 are the most common and have been proven to cause 90% of genital warts in men [9–12]. In addition, with the in-depth study of HPV, it is found that high-risk HPV is highly correlated with various malignant tumors such as anal cancer, head and neck cancer, and throat cancer [13–15].

The genome of HPV is comprised of an early region (E1, E2, E4, E5, E6 and E7), a late region (L1 and L2), a small non-coding region (NCR) and a long control region (LCR) [16, 17]. Among these genes, the early genes E6 and E7 are regarded as the main oncogenes, and their expression is essential for the transformation and maintenance of the malignant state. The E6 protein binds to the ligase E6AP, forming a complex that can inactivate the p53 tumor suppressor protein, whereas the E7 protein prevents expression of the retinoblastoma (Rb) tumor suppressor protein through ubiquitination [18, 19]. When human papillomavirus integrates into the host cell, E6 and E7 are invariably reserved and uncontrolled expressed so the cell obtains the function of immortalization and transformation [20]. The antigenic epitope of HPV E6 and E7 proteins are ideal targets for therapeutic vaccine design, so it is very important to identify the epitopes of E6 and E7 proteins. The HPV LCR contains the viral origin of replication, the viral early promoter and transcriptional enhancer, the late polyadenylation site and the late regulatory element and which has a variety of the regulatory sites for the viral factors and cellular transcriptional factors, such as E1, E2 and SOX2 [21–24]. The LCR has been shown to be the most variable region of HPV genome, because it does not encode any gene and can able to accumulate and tolerate more mutations. The LCR variants have been shown to differently regulate the replication of HPV throughout the viral life cycle and the transcriptional activity of E6 and E7 [25, 26].

There have been reports about the E6, E7 and LCR of HPV-16 and HPV-58 in recent years. However, studies of the HPV-52 are few, especially for the LCR. In addition, statistics shown that the infection rate of HPV-52 among Sichuan women has increased year by year. Therefore, it is necessary to carry out in-depth research on the nucleotide polymorphisms with the HPV-52 E6, E7 and LCR. This study identified the single nucleotide polymorphism (SNP) in the HPV-52 E6, E7 and LCR in Sichuan, southwest China, assessed the possible association of polymorphisms in the HPV-52 E6, E7 and LCR with the virus infection, propagation and replication, predicted the high affinity antigen epitope of HPV-52 E6 and E7, and analyzed the binding sites of the transcription factors in the LCR. Our results could provide basic data for further studies on the HPV-52 epidemiology, prevention, and therapeutic vaccine development.

## Methods

### Ethics statement

All participants were informed of the study aims, and a written informed consent was received from each patient before sample collection, and the patients' privacy have been fully protected. This study was approved by education and research committee and Ethics Committee of Sichuan University, China (approval number SCU20100196494), and was carried out in line with the Helsinki Declaration.

### Collection of clinical specimens

3432 cervical scrape cell samples were collected from outpatients who underwent routine cervical screenings at The Affiliate Reproductive Hospital of Sichuan Genitalia Hygiene Research Center, Chengdu SongZiNiao Sterility Hospital and Chengdu Medical College Affiliated Infertility Hospital from December 2018 to December 2019. The sample was stored at − 20 °C in cell preservation solution and all methods were performed in accordance with the relevant guidelines and regulations.

### Genomic DNA extraction and HPV typing

HPV DNA was extracted and genotyped using Advanced Fragment Analysis (AFA) based on capillary electrophoresis system with the commercial Human Papillomavirus Genotyping Kit for 25 types (HEALTH Gene technologies, Ningbo, China) according to the manufacturer’s guidelines. This kit is able to classify 25 different HPV types (HPV 16, 18, 26, 31, 33, 35, 39, 45, 51, 52, 53, 56, 58, 59, 66, 68, 73, 82, 6, 11, 42, 43, 44 and 83). The existence of other types of HPV may have a certain impact on HPV-52, only samples that tested positive for single infection of HPV-52 were picked out for amplification, sequencing and following study to ensure the single of variables.

### DNA amplification and sequencing

The primer pairs were designed by Primer premier 5.0 according to the HPV-52 reference sequence (GenBank: X74481.1) and the primer sequences were: HPV-52 E6 F: 5′-ACCCACAACCACTTTTTTTTAT-3′, HPV-52 E6 R: 5′-CACCATCTGTATCCTCCTCATC-3′; HPV-52 E7 F: 5′-TTGTCAAACGCCATTATGTCCT-3′, HPV-52 E7 R: 5′-TTGCCTCTACTTCAAACCAGCC-3′; HPV-52 LCR F: 5′-GCCCAAACTAAAACGCCCT-3′, HPV-52 LCR R: 5′-CACCGATTCTTCCAGCACC-3′. All the primers were synthesized by TSINGKE, China. The E6, E7 and LCR fragments were amplified in 50 μl PCR reaction volumes containing 18 μl of extracted DNA, 25 μl Taq 2X PCR Master Mix with Dye (ABclonal), 1 μl forward primer, 1 μl reverse primer and 5 μl Nuclease-free water.

The PCR amplification was performed under the following conditions: an initial 30 s denaturation step at 95 °C, followed by 30 amplification cycles, with each cycle including a 30 s denaturation step at 95 °C, a 30 s annealing step at 52–55 °C (52 °C for E6, 55 °C for E7 and LCR), and a 35–65 s (35 s for E7, 45 s for E6 and 65 s for LCR) elongation step at 68 °C, and then 5 min final extension at 68 °C, ended up and held at 4 °C. The PCR products were examined under UV light after electrophoretic separation on a 2% agarose gel. The positive fragments were subjected to the bi-directional DNA sequencing (TSINGKE, China) for the further analysis.

### Variant identification and analysis

To identify the SNP in E6, E7 and LCR, the HPV-52 E6/E7/LCR sequences were aligned to the HPV-52 reference sequence (GenBank: X74481.1) by MEGA 7.0 after sequencing. The secondary structures of the HPV-52 E6 and E7 proteins were predicted by PSIPRED 4.0 (http://bioinf.cs.ucl.ac.uk/psipred/) using the default parameters.

The neighbor-joining phylogenetic tree based on the HPV-52 E6/E7/LCR was constructed by MEGA 7.0 using the Kimura 2-parameter model and the number of bootstrap replications was set at 1000. To construct the phylogenetic branches, the following reference sequences were used: X74481 ((A1), HQ537739 (A2), HQ537740 (B1), HQ537743 (B2), HQ537744 (C1), HQ537746 (C2), HQ537748 (D) [27].

To estimate the selection pressure acting on the HPV-52 E6 and E7 protein coding regions, the non-synonymous and synonymous nucleotide divergence were calculated by CODEML in PAML 4.9. Using the Bayes empirical Bayes (BEB) analysis, the sites with the posterior probability > 95% were identified as the positively selected sites [23].

### T and B cell antigen epitope prediction

The Immune Epitope Database (IEDB) server (http://www.iedb.org/) was used to predict the major HLA-I and HLA-II binding peptides [28, 29]. In our study, we used IEDB recommended methods to predict the epitopes against 34 HLA-I alleles by comprehensive analysis the frequency of Chinese in allele frequency net database (AFND) (http://www.allelefrequencies.net/hla6006a.asp) and 27 HLA-II allele [30]. (The more details see Additional file 1: Table S1 and S2). According to the IEDB recommended method, among HLA-I a low percentile rank (PR) showed a good binder, among HLA-II a low adjusted rank (AR) showed a good binder. In prediction of HLA-I and HLA-II restricted epitopes the PR ≤ 1.0 and AR ≤ 5.0 were selected for the further analysis, respectively [29, 31].

The ABCpred server (https://webs.iiitd.edu.in/raghava/abcpred/index.html) was used to predict B cell antigen epitopes of HPV-52 E6/E7 reference and variant sequences according to the default parameters[32]. The higher predicted score represented a better affinity [29, 31]. The server is able to predict epitopes with 65.93% accuracy using recurrent neural network.

### Transcription factor binding sites prediction

To analyze the transcription factor binding sites in the LCR of HPV-52, the JASPAR database (http://jaspar.genereg.net/) was used [33]. The sites for CEBPA, CEBPB, CREB1, ELK4, ESR2, ETS1, FOS, FOXC1, FOXL1, FOXP3, HOXA5, HOXC11, HSF1, IRF2, JUN, NFIA, NFKB1, PHOX2A, POU2F2, RAX, SMAD3, SOX9, SOX10, SPIB, SRY, STAT1, VAX1 and YY1 were included. The relative profile score threshold was set at 85% [23].

## Results

### HPV prevalence in Sichuan China

**Fig. 1:**
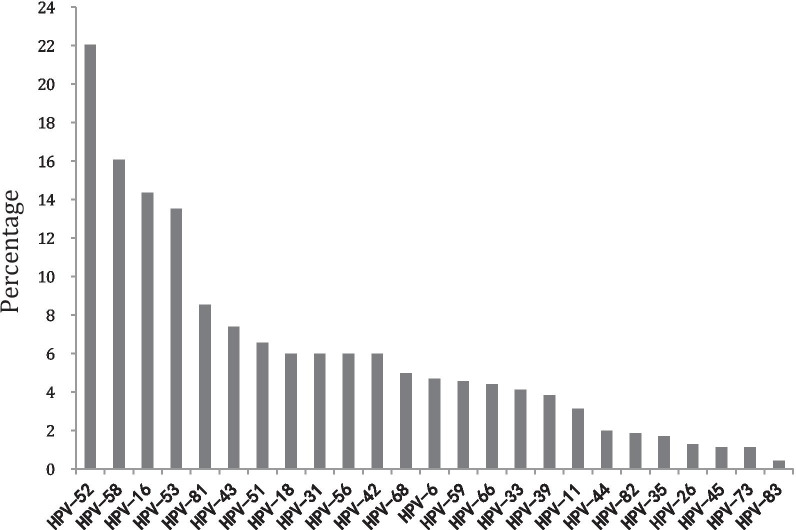
Prevalence of HPV in Sichuan

Of the 3432 collected outpatient samples, 703 samples (20.48%, 703/3432) were HPV positive including single infection (70.41%, 495/703) and coinfections (70.41%, 495/703). Up to six infections were found in coinfections, double infection accounted for 19.06%, triple infection accounted for 6.54%, quadruple infection accounted for 2.42%, fifth infection accounted for 1.42%, and sixth infection accounted for 0.14% (Fig. 1). By genotyping, 25 HPV types were identified, including high-risk HPV (HPV-16, 18, 26, 31, 33, 35, 39, 45, 51, 52, 53, 56, 58, 59, 66, 68, 73 and 82) and low-risk HPV (HPV-6, 11, 42, 43, 44, 81 and 83). Of the types observed, HPV-52 (22.05%, 155/703), 58 (16.07%, 113/703) and 16 (14.37%, 101/703) were the three most common types in the positive samples. The number of HPV52 single infection samples was 46.

### Genomic polymorphisms of HPV-52 E6/E7/LCR

Although 46 HPV-52 positive single infection samples were detected, only 41 entire E6/E7/LCR sequences were obtained and further analyzed. It is might be the possible explanation for not all sequences successfully amplified that the small copy number of HPV in some samples. Our data showed that the total length of the HPV-52 E6 Open Reading Frame (ORF) was 447 bp and that of E7 was 300 bp, which were consistent with the reference sequence. LCR was only 879 bp, with a 10 bp deletion compared with the reference sequence in 97.56% (40/41) samples. A total of 50 SNP sites and 3 deletions were identified across the E6, E7 and LCR. The combinations of all these SNP sites made for 12 unique E6/E7/LCR sequences (variant no.: 1-12, Table 1). The 12 variant had the most variation compared with reference sequence.

**Table 1:** HPV 52 E6/E7/LCR nucleotide mutation sites (variable sites only)

Variant ID	E6						E7										LCR																																		N	Lineage
																	7	7	7	7	7	7	7	7	7	7	7	7	7	7	7	7	7	7	7	7	7	7	7	7	7	7	7	7								
	1	3	3	3	3	5	5	6	7	7	7	7	7	7	8	8																																				
																	1	2	3	3	3	4	4	4	5	5	5	6	6	6	6	6	6	6	7	7	7	7	8	8	8	9	9	9	1	1	2	6	7	8		
	0	5	5	7	7	3	7	6	0	0	2	3	4	5	0	4																																				
																	6	0	7	7	9	1	2	9	0	8	8	2	2	5	5	7	9	9	1	1	4	8	6	6	8	1	3	3	3	5	1	1	6	3		
	8	0	6	8	9	0	3	2	6	7	7	3	2	1	1	8																																				
																	8	7	1	7	3	4	1	5	9	0	6	2	4	7	9	1	3	6	2	3	3	8	1	5	9	7	3	8								
X74481.1	G	G	G	A	A	A	T	C	A	G	T	C	G	C	A	T	G	C	G	T	T	T	G	T	C	A	G	G	T	A	T	C	G	T	G	G	C	A	G	A	C	C	T	A	T	A	G	C	G	G		A1
1	–	T	–	–	G	–	–	–	–	–	–	–	–	T	G	–	C	A	T	–	–	–	–	–	–	–	–	A	G	C	C	–	–	–	C	–	–	–	A	G	–	–	C	G	C	–	–	–	–		12	B2
2	–	T	–	–	G	–	–	–	–	–	–	–	–	T	G	–	C	A	T	–	–	–	–	–	–	–	–	A	G	C	C	–	–	–	C	–	–	–	A	G	–	–	–	G	C	–	–	–	–	–	11	B2
3	–	T	A	–	G	–	–	–	–	–	–	–	–	T	G	–	C	A	T	–	–	–	–	–	–	–	–	A	G	C	C	–	–	–	C	–	–	–	A	G	–	–	–	–	C	–	–	–	–	–	5	B2
4	–	T	–	C	G	–	–	–	–	–	–	–	–	T	G	–	C	A	T	–	–	–	–	–	–	–	–	A	G	C	C	–	–	–	C	–	–	–	A	G	–	–	–	–	C	–	–	–	–	–	3	B2
5	–	T	–	–	G	–	–	–	–	–	–	–	–	T	G	–	C	A	T	–	–	–	–	–	–	–	–	A	G	C	C	–	–	–	C	–	–	–	A	G	–	–	–	–	C	–	–	–	–	–	3	B2
6	C	T	–	–	G	–	–	–	–	–	–	–	–	T	G	–	C	A	T	–	–	–	–	–	–	–	–	A	G	C	C	–	–	–	C	–	–	–	A	G	–	–	C	G	C	C	–	–	–	–		B2
7	–	T	A	–	G	–	–	–	–	–	–	–	–	T	G	–	C	A	T	–	–	–	–	–	–	–	–	A	G	C	C	–	–	–	C	–	–	–	A	G		–	C	–	C	–	–	–	–	–		B2
8	–	T	A	–	G	–	–	–	–	–	–	–	–	T	G	–	C	A	T	C	–	–	–	–	–	–	–	A	G	C	C	–	–	–	C	–	–	–	A	G	–	–	–	–	C	–	–	T	–	–		B2
9	–	T	–	–	G	–	–	–	–	–	–	–	–	T	G	–	C	A	T	–	–	–	–	–	–	–	–	A	G	C	C	–	–	–	C	–	–	–	A	G	–	–	C	–	C	–	–	–	–	–		B2
10	–	T	–	–	G	–	–	–	–	–	–	–	–	T	G	–	C	A	T	–	–	–	–	G	–	–	–	A	G	C	C	–	–	–	C	–	–	–	A	G	–	–	–	–	C	–	–	–	–	–		B2
11	–	T	–	–	G	–	–	–	–	–	–	–	–	T	G	–	C	A	T	–	–	–	–	–	–	–	–	A	G	C	C	–	–	–	C	–	–	–	A	G	T	–	–	G	C	–	T	–	C	T		B2
12	–	T	–	–	–	G	A	T	G	A	G	T	A	–	G	G	–	–	–	–	C	C	A	–	G	C	A	A	G	–	C	T	A	C	C	A	T	G	A	–	–	A	–	–	–	–	–	–	–	–		C2
	T7				K			T	S	S	Y	H	D			L																																				
AA	E				9			3	5	5	5	6	6			9																																				
changes	3				3			7	2	2	9	1	4			9																																				
	^Q^				R			I	D	D	D	Y	N			R																																				

The nucleotides matching the reference (GenBank: X74481.1) are marked with a dash (–).The C and S in the row of secondary structure designate "coil" and "strand", respectively

For E6, 6 SNP sites were observed compared with the reference sequence and the combinations of all these SNP made for 5 unique E6 sequences. G350T and A379G were the most variable sites and were observed in 100% and 97.56% (40/41) of the samples, respectively. G108C and A379G, leading to the amino acids substitution of E3Q and K93R, respectively. A378C and A379G together led to the amino acid substitution of K93R. G350T, G356A and A530G were synonymous mutation.

For E7, 10 SNP sites were observed compared with the reference sequence and the combinations of all these SNP made for 2 unique E7 sequences. A801G and C751T were the most variable sites and were observed in 100% and 97.56% (40/41) of the samples, respectively. C662T, AG706/707GA, T727G, C733T, G742A and T848G, leading to the amino acids substitution of T37I, S52D, Y59D, H61Y, D64N and L99R, respectively. T573A, C751T and A801G were synonymous mutation. No deletion or insertion mutation sites were found in either E6 or E7 sequences. The secondary structures also had not changed in either E6 or E7 protein.

For LCR, 34 SNP sites and 3 deletions were observed compared with the reference sequence and the combinations of all these mutations made for 9 unique LCR sequences. G7622A, T7624G, T9659C, G7712C and G7861A were observed in all samples. G7168C, C7207A, G7371, A7657C, A7865G and T13C were observed in 97.56% (40/41) of the samples. Two deletions were found between the nucleotide sites 7387 to 7391 (TTATG) and 7698 to 7700 (CTT) in all samples. A deletion was found between the nucleotide sites 7287 to 7288 (TG) in 97.56% (40/41) of the samples. Also, T7933C and A7938G were the common variable sites and were observed in 36.59% (15/41) and 60.98% (25/41) of the samples, respectively.

### Phylogenetic analysis

**Fig. 2:**
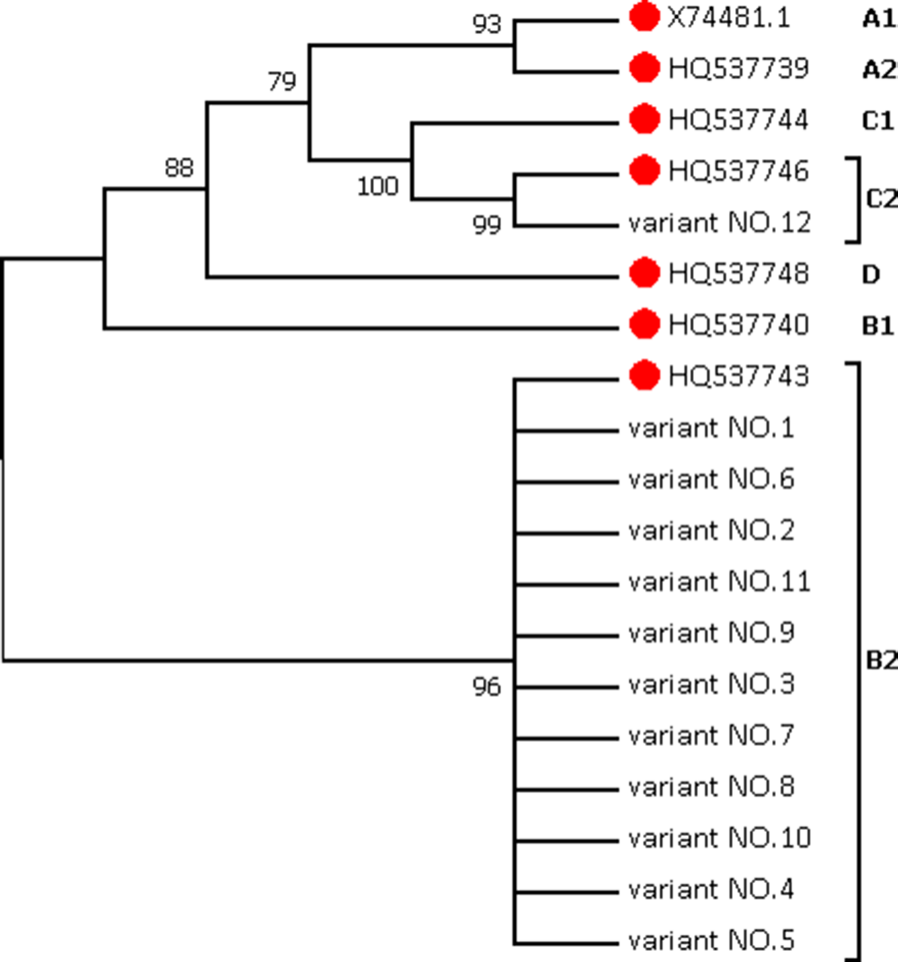
Neighbor-joining phylogenetic tree based on HPV-52 E6/E7/LCR sequences, and reference sequences of sub-lineages were marked with red dot

The neighbor-joining phylogenetic trees were constructed by MEGA 7.0, using the 12 unique HPV-52 E6/E7/LCR variant sequences and 7 sub-lineages reference sequences. The phylogenetic tree (Fig. 2) showed that all variants were clustered in sub-lineage B2, except variant NO.12 (sub-lineage C2). It was basically consistent with a study related to HPV-52 in Japan [34].

### Selective pressure analysis

The selective pressure analysis results by the Bayes empirical Bayes (BEB) analysis of PAML 4.9 were indicated that: in the HPV-52 E6 and E7 protein coding sequences, no positive selection sites were observed.

### HLA-I and HLA-II binding peptides prediction

To evaluate the impacts of the HPV-52 E6 and E7 sequence polymorphisms on the immune recognition of antigens, the binding peptides for both HLA I and HLA II were predicted by the IEDB servers. Based on the principle of epitope selection described in the methods section, 273 and 275 E6 HLA I predicted epitopes were selected form the E6 reference and variant sequences, respectively (the more details see Additional file 2: Table S3 and S4). HLA-A*30:02 was the most frequency, followed by HLA-C*06:02, HLA-C*07:02 and HLA-B*38:01. HLA-A*11:01 (_86–94_KTLEERVKK), HLA-A*33:03 (_127–135_NIMGRWTGR) and HLA-A*30:01 (_91–99_RVRKPLSEI/_91–99_RVKKPLSEI) showed the best binding affinity (PR = 0.01). 134 and 114 E7 HLA I predicted epitopes were selected form the E7 reference and variant sequence, respectively (the more details see Additional file 3: Table S5 and S6). HLA-A*01:01 was the most frequency, followed by HLA-B*44:02, HLA-B*44:03 and HLA-A*30:02. HLA-B*44:02 (_47–55_AEQATSNYY/_47–55_AEQATDNYY), HLA-B*44:03 (_47–55_AEQATSNYY/_47–55_AEQATDNYY) and HLA-B*15:01 (_45–54_GQAEQATSNY) showed the best binding affinity (PR = 0.01).

556 E6 HLA II predicted epitopes were selected in both of the E6 reference and variant sequence (The more details see Additional file 4: Table S7 and S8). The three best epitopes were HLA-DPA1*01:03/DPB1*04:01, HLA-DRB1*03:01 and HLA-DRB4*01:01. Due to K93R, there was a potentially affected on the epitope HLA-DPA1*02:01/DPB1*01:01. 101 and 120 E7 HLA II predicted epitopes were selected form the E7 reference and variant sequence, respectively (The more details see Additional file 5: Table S9 and S10). The three best epitopes were HLA-DQA1*03:01/DQB1*03:02, HLA-DRB3*01:01 and HLA-DQA1*05:01/DQB1*02:01. Due to Y59D, H61Y and D64N, there were a potentially affected on the epitopes HLA-DRB3*02:02 and HLA-DQA1*01:02/DQB1*06:02.

### B cell binding peptides prediction

In HPV-52 E6, a total of 13 B cell potential epitopes were predicted in both of the reference and variant sequences. The most potent epitopes were _129–144_MGRWTGRCSECWRPRP and _108–123_TPLCPEEKERHVNANK. Due to K93R, the score of epitopes _89–104_EERVKKPLSEITIRCI and _81–96_YSLYGKTLEERVKKPL changed from 0.87 to 0.90 and 0.81 to 0.84, respectively; due to E3Q, the score of epitope _3–18_EDPATRPRTLHELCEV changed from 0.68 to 0.74. The other prediction epitopes were consistent between reference and variant sequences, and no increased epitopes in the variant sequences (The more details see Additional file 6: Table S11).

In HPV-52 E7, a total of 10 B cell potential epitopes were predicted in both of the reference and variant sequences. The most potent epitopes were _23–38_HCYEQLGDSSDEEDTD and _34–49_EEIDGVDRPDGQAEQ. Due to T37I, S52D, Y59D, H61Y and D64N, the prediction epitopes of reference and variant sequences had some differences, only 4 prediction epitopes were completely consistent between reference and variant sequences, an increased high affinity epitope _44–59_DGQAEQATDNYYIVTD was discovered in the variant sequences (the more details see Additional file 6: Table S12).

### Prediction of the transcription factor binding sites

The online JASPAR database was used to investigate the potential binding sites for the transcription factors in HPV-52 LCR reference and variant sequences. The results showed that LCR region had high-affinity binding sites for transcription factors of SOX9, FOS, RAX, HOXA5, VAX1 and SRY. G7622A and T7624G, G7861A and C7917A/G76C lead to increase the binding sites for FOXC1, RAX/VAX1/HOXA5/PHOX2A and FOXL1, respectively. In addition, the nucleotide sites 21, 7168, 7414, 7580, 7865 and 7983 potentially affected the binding sites for FOXP3, CEBPB, SRY, CEBPB, VAX1 and VAX1/RAX/HOXA5, respectively.

## Discussion

The persistent infection with high-risk HPV types is the main cause in triggering the development of cervical cancer, such as the types 16, 18, 52 and 58 [35]. HPV 52 is one of the most relevant HPV types especially in Southeast Asia, where it causes up to 20% of all cervical cancer [36, 37]. Through preliminary exploration in the early stage, we found that HPV-52 accounting for 22.05% of all the HPV-positive samples was the most common high-risk types, followed by HPV-58 and HPV-16 in Sichuan, China. In addition, other studies have shown that the distribution of HPV-52 has a certain regional distribution, mainly related to cervical cancer in Asian countries like China and South Korea [38, 39]. Compared with the reference sequence, the HPV-52 variants were clustered in sub-lineage B2 and C2, no variants belonged to the lineage A and D in our study. In addition, the HPV-52 LCR variants from Sichuan, China have not been reported yet. Our study showed that the variation of the HPV-52 LCR was showed in a higher ratio than those of the E6 and E7, the nucleotide variations were LCR > E7 > E6 found in 3.82%, 3.33% and 1.34%, respectively. The most common non-synonymous substitution in the HPV-58 E6 was A278G (K93R), and E7 was only one sample with non-synonymous. The viral proteins E6 and E7 function as the main regulators of HPV-induced tumorigenesis, and changes in amino acids may influence the transforming activity of the E6 and E7 oncoproteins [40]. Identifying new variants in HPV-52 E6/E7 may inform the rational design of new vaccines specifically for women in southwest China.

Currently, the majority of therapeutic vaccines target HPV oncoproteins E6 and E7 with the aim to deliver E6 and E7 antigens in various forms to antigen presenting cells in order to activate HPV antigen-specific CD8 + cytotoxic T cells or CD4 + helper T cells, respectively. Importantly, E6 and E7 antigens need to be processed and digested by proteasomes into smaller peptides before they can be presented on the HLA-I molecule of the APCs for the activation of CD8 + T cells [31]. However, not all peptide fragments from the antigenic proteins are loaded on HLA molecules and recognized by antigen-specific T cells. Only a selected few of these short peptides contain the sequence of antigenic fragments (epitopes) that can bind to the HLA molecule with high affinity and subsequently interact with the T cell receptor of antigen-specific T cells to elicit an immune response [28, 41, 42].

The amino acid changes in E6 and E7 oncoproteins can influence the HLA binding peptides and have a significant immunological effect on the immune system’s ability of recognition of these viral antigens. We had identified the high affinity epitopes of E6/E7 oncoproteins for T cells and B cells, providing certain basic data for the development of therapeutic vaccines. In our study, SNP sites were common in E6 and E7, but the existence of these SNP sites had almost no effect on immune recognition. The two non-synonymous mutations of E6, E3Q and K93R, had almost no effect on the high-affinity epitopes of T and B cell; E7 non-synonymous mutations were found in only one sample, and these non-synonymous mutations enhanced its affinity with B cell. These findings are undoubtedly good news for vaccine development. Even with the presence of SNP sites, the effectiveness of the vaccine is still guaranteed.

Unlike prophylactic HPV vaccines, which are used to generate neutralizing antibodies against viral particles, therapeutic HPV vaccines are used to stimulate cell-mediated immune responses to specifically target and kill infected cells. In the cell mediated immune responses, cytotoxic T lymphocytes (CTLs) were considered as the major eradicators of both HPV-infected cells and cervical cancer [43]. HPV oncoproteins E6 and E7 are responsible for the malignant progression of HPV-associated diseases and are consistently expressed in HPV-associated diseases and cancer lesions. E6 and E7 act as the promising specific tumor antigens and are available as the therapeutic targets [44]. Furthermore, therapeutic HPV vaccines targeting E6 and E7 can circumvent the problem of immune tolerance against selfantigens because these virus encoded oncogenic proteins are foreign proteins to human bodies [41].

Mutations on LCR may influence the binding sites and the function of it. The HPV LCR which contains the binding sites for both viral and cellular factors, has shown regulatory functions on replication of HPV, transcriptional activity of the E6/E7 and the other interaction through the virus life cycle [25, 38]. In our study, VAX1, CEBPB, FOXL1, PHOX2A and HOXA5 were the transcription factors that may be affected in HPV-52. One study had shown that VAX1 was closely related to bladder cancer recurrence [45]. CEBPB is a leucine-zipper transcription factor that regulates growth and differentiation of hematopoietic and epithelial cells. One study based on breast cancer found that CEBPB was a novel transcriptional regulator of CLDN4. The upregulation of CEBPB-CLDN4 signaling caused the migration and invasion of cancer cell [46]. FOXL1 is a member of the Forkhead box (FOX) superfamily and was reported to be dysregulated in various types of cancers [47]. PHOX2A was a transcription factor involving in cell proliferation and migration in lung cancer [48]. HOXA5 is a member of the homeobox (HOX) family and is upregulated in many types of tumors [48, 49].

In addition, SOX10, a transcription factor of the sex determining region Y (SRY)-related high motility group (HMG)-box gene family, playing an important role in cancer progression, including tumorigenesis, changes in the tumor microenvironment, and metastasis [50, 51]. Studies had shown that FOS was closely related to the pathogenesis of bone tumors in mice [52]. The study by Yang M et al. showed that in addition to stimulating PKR activity, RAX can positively regulate both SV40 large T antigen-dependent DNA replication and transcription in a mechanism that may alter the interaction of the cellular factor(s) with the SV40 enhancer via the dsRNA-binding domains of RAX [53]. This function of RAX may have implications for regulation of HPV replication and transcription because of the many similarities between the viral and cellular processes. SOX9, FOS, RAX and SRY were the high probability binding sites in HPV-52 LCR.

## Conclusion

In conclusion, this study investigated the gene polymorphisms and phylogeny of high-risk HPV-52 E6/E7/LCR, the possible influence of non-synonymous substitutions in E6/E7 on the T-cell and B-cell response and the impacts of the LCR variations on the bindings of the cellular transcription factors from Southwest China. Knowledge of genetic variation in HPV may be useful as an epidemiologic correlate of cervical cancer risk, or may even provide critical information for developing diagnostic probes. Although our study showed some limitations on sample capacity and source, it provided more basic data for the further immunotherapeutic approaches and vaccine development strategies. It also helps performing further study to demonstrate the biological function of HPV-52 E6/E7/LCR variants and the effect of multiple infection of high-risk HPV on tumor progression. The TFBS we found are still need deeper exploration for the potential of them to be marker in diagnosis and therapy.

## Supplementary Information

**Additional file 1: Table S1**. HLA-I alleles and lengths selected for prediction. **Table S2**. HLA-II alleles and lengths selected for prediction.

**Additional file 2: Table S3**. Prediction results for HPV52 E6 reference sequence HLA-I epitope peptides. **Table S4**. Prediction results for HPV52 E6 variant sequence HLA-I epitope peptides.

**Additional file 3: Table S5**. Prediction results for HPV-52 E7 reference sequence HLA-I epitope peptides. **Table S6**. Prediction results for HPV-52 E7 variant sequence HLA-I epitope peptides.

**Additional file 4: Table S7**. Prediction results for HPV-52 E6 reference sequence HLA-II epitope peptides. **Table S8**. Prediction results for HPV-52 E6 variant sequence HLA-II epitope peptides.

**Additional file 5: Table S9**. Prediction results for HPV-52 E7 reference sequence HLA-II epitope peptides. **Table S10**. Prediction results for HPV-52 E7 variant sequence HLA-II epitope peptides.

**Additional file 6: Table S11**. Predicted linear B cell epitopes of HPV-52 E6. **Table S12**. Predicted linear B cell epitopes of HPV-52 E7.

## Data Availability

All data generated or analyzed during this study are included in this published article and GenBank.

## Abbreviations

DNA: Deoxyribonucleic acid
HPV: Human papillomavirus
IEDB: The immune epitope database
LCR: Long control region
MEGA: Molecular evolutionary genetics analysis
NCBI: National Center for Biotechnology Information
PCR: Polymerase Chain Reaction
SNP: Single nucleotide polymorphism
PAML: Phylogenetic Analysis by Maximum Likelihood
TFBS: Transcription factor binding site
The amino acid shorthand A, C, D, E, F, G, H, I, K, L, M, N, P, Q, R, S, T, V, W and Y: Alanine, cysteine, aspartic acid, glutamic acid, phenylalanine, glycine, histidine, isoleucine, lysine, leucine, methionine, asparagine, proline, glutamine, arginine, serine, threonine, valine, tryptophan and tyrosine
