# Supplementary material for: The genetic variability, phylogeny and functional significance of E6, E7 and LCR in human papillomavirus type 52 isolates in Sichuan, China
Source: Virol J. 2021 May 3;18:94. doi: 10.1186/s12985-021-01565-5 (PMC8091156; doi:10.1186/s12985-021-01565-5)
Supplement: Supplementary file 1 — Additional file 1: Table S1. HLA-I alleles and lengths selected for prediction. Table S2. HLA-II alleles and lengths selected for prediction. [file 12985_2021_1565_MOESM1_ESM.pdf]

Table S1 HLA-I alleles and lengths selected for prediction

| Alleles | Length | Alleles | Length | Alleles | Length |
|---|---|---|---|---|---|
| HLA-A*01:01 | All lengths | HLA-B*15:02 | All lengths | HLA-B*52:01 | All lengths |
| HLA-A*02:01 | All lengths | HLA-B*15:25 | All lengths | HLA-B*58:01 | All lengths |
| HLA-A*02:06 | All lengths | HLA-B*35:01 | All lengths | HLA-B*58:02 | All lengths |
| HLA-A*11:01 | All lengths | HLA-B*35:03 | All lengths | HLA-C*01:02 | All lengths |
| HLA-A*24:02 | All lengths | HLA-B*38:01 | All lengths | HLA-C*03:02 | All lengths |
| HLA-A*26:01 | All lengths | HLA-B*40:01 | All lengths | HLA-C*03:04 | All lengths |
| HLA-A*30:01 | All lengths | HLA-B*40:02 | All lengths | HLA-C*04:01 | All lengths |
| HLA-A*30:02 | All lengths | HLA-B*44:02 | All lengths | HLA-C*04:03 | All lengths |
| HLA-A*33:03 | All lengths | HLA-B*44:03 | All lengths | HLA-C*06:02 | All lengths |
| HLA-B*13:01 | All lengths | HLA-B*46:01 | All lengths | HLA-C*07:02 | All lengths |
| HLA-B*13:02 | All lengths | HLA-B*51:01 | All lengths | HLA-C*08:01 | All lengths |
| HLA-B*15:01 | All lengths | | | | |

Table S2 HLA-II alleles and lengths selected for prediction

| Alleles | Length | Alleles | Length |
|---|---|---|---|
| HLA-DRB1*01:01 | 12-18 | HLA-DQA1*05:01/DQB1*02:01 | 12-18 |
| HLA-DRB1*03:01 | 12-18 | HLA-DQA1*05:01/DQB1*03:01 | 12-18 |
| HLA-DRB1*04:01 | 12-18 | HLA-DQA1*03:01/DQB1*03:02 | 12-18 |
| HLA-DRB1*04:05 | 12-18 | HLA-DQA1*04:01/DQB1*04:02 | 12-18 |
| HLA-DRB1*07:01 | 12-18 | HLA-DQA1*01:01/DQB1*05:01 | 12-18 |
| HLA-DRB1*08:02 | 12-18 | HLA-DQA1*01:02/DQB1*06:02 | 12-18 |
| HLA-DRB1*09:01 | 12-18 | HLA-DPA1*02:01/DPB1*01:01 | 12-18 |
| HLA-DRB1*11:01 | 12-18 | HLA-DPA1*01:03/DPB1*02:01 | 12-18 |
| HLA-DRB1*12:01 | 12-18 | HLA-DPA1*01:03/DPB1*04:01 | 12-18 |
| HLA-DRB1*13:02 | 12-18 | HLA-DPA1*03:01/DPB1*04:02 | 12-18 |
| HLA-DRB1*15:01 | 12-18 | HLA-DPA1*02:01/DPB1*05:01 | 12-18 |
| HLA-DRB3*01:01 | 12-18 | HLA-DPA1*02:01/DPB1*14:01 | 12-18 |
| HLA-DRB3*02:02 | 12-18 | | |
| HLA-DRB4*01:01 | 12-18 | | |
| HLA-DRB5*01:01 | 12-18 | | |
