# Supplementary material for: The genetic variability, phylogeny and functional significance of E6, E7 and LCR in human papillomavirus type 52 isolates in Sichuan, China
Source: Virol J. 2021 May 3;18:94. doi: 10.1186/s12985-021-01565-5 (PMC8091156; doi:10.1186/s12985-021-01565-5)
Supplement: Supplementary file 2 — Additional file 2: Table S3. Prediction results for HPV52 E6 reference sequence HLA-I epitope peptides. Table S4. Prediction results for HPV52 E6 variant sequence HLA-I epitope peptides. [file 12985_2021_1565_MOESM2_ESM.pdf]

Table S3 Prediction results for HPV52 E6 reference sequence HLA-I epitope peptides

allele	start	end	length	peptide	percentile rank (PR)
HLA-A*33:03	127	135	9	NIMGRWTGR	0.01
HLA-A*11:01	86	94	9	KTLEERVKK	0.01
HLA-A*30:01	91	99	9	RVKKPLSEI	0.01
HLA-C*06:02	76	84	9	YRHYQYSLY	0.02
HLA-A*30:01	86	94	9	KTLEERVKK	0.03
HLA-A*30:02	68	76	9	RFLSKISEY	0.04
HLA-B*38:01	54	62	9	YRDNNPYGV	0.04
HLA-B*15:25	52	60	9	IVYRDNNPY	0.04
HLA-A*01:01	73	81	9	ISEYRHYQY	0.04
HLA-B*44:02	35	43	9	KELQRREVY	0.05
HLA-B*44:03	35	43	9	KELQRREVY	0.05
HLA-B*15:01	37	45	9	LQRREVYKF	0.05
HLA-B*15:02	52	60	9	IVYRDNNPY	0.05
HLA-A*01:01	47	60	14	FTDLRIVYRDNNPY	0.05
HLA-A*01:01	47	54	8	FTDLRIVY	0.05
HLA-C*06:02	54	62	9	YRDNNPYGV	0.05
HLA-C*07:02	76	84	9	YRHYQYSLY	0.06
HLA-A*02:01	18	26	9	VLEESVHEI	0.06
HLA-B*15:01	52	60	9	IVYRDNNPY	0.06
HLA-C*04:03	18	26	9	VLEESVHEI	0.07
HLA-A*02:01	45	53	9	FLFTDLRIV	0.07
HLA-A*30:02	52	60	9	IVYRDNNPY	0.07
HLA-A*33:03	47	55	9	FTDLRIVYR	0.07
HLA-C*07:02	54	62	9	YRDNNPYGV	0.07
HLA-C*06:02	38	46	9	QRREVYKFL	0.08
HLA-B*15:25	37	45	9	LQRREVYKF	0.09
HLA-C*06:02	7	15	9	TRPRTLHEL	0.09
HLA-B*46:01	52	60	9	IVYRDNNPY	0.10
HLA-A*02:06	45	53	9	FLFTDLRIV	0.10
HLA-A*30:01	6	14	9	ATRPRTLHE	0.10
HLA-A*33:03	69	77	9	FLSKISEYR	0.11
HLA-A*33:03	46	55	10	LFTDLRIVYR	0.11
HLA-C*07:02	68	76	9	RFLSKISEY	0.11
HLA-C*07:02	7	15	9	TRPRTLHEL	0.12
HLA-A*30:02	73	81	9	ISEYRHYQY	0.13
HLA-C*06:02	140	148	9	WRPRPVTQV	0.13
HLA-C*06:02	76	83	8	YRHYQYSL	0.14
HLA-B*35:01	52	60	9	IVYRDNNPY	0.15
HLA-A*02:06	18	26	9	VLEESVHEI	0.15
HLA-C*03:02	52	60	9	IVYRDNNPY	0.15
HLA-C*07:02	39	47	9	RREVYKFLF	0.15

HLA-C*04:01	54	62	9	YRDNNPYGV	0.16
HLA-B*52:01	79	88	10	YQYSLYGKTL	0.17
HLA-C*06:02	39	47	9	RREVYKFLF	0.17
HLA-B*40:02	74	83	10	SEYRHYQYSL	0.18
HLA-B*40:01	74	83	10	SEYRHYQYSL	0.18
HLA-A*30:02	72	81	10	KISEYRHYQY	0.18
HLA-A*33:03	126	135	10	HNIMGRWTGR	0.18
HLA-B*44:02	113	125	13	EEKERHVNANKRF	0.18
HLA-C*07:02	80	88	9	QYSLYGKTL	0.18
HLA-A*30:02	51	60	10	RIVYRDNNPY	0.19
HLA-B*13:01	79	88	10	YQYSLYGKTL	0.20
HLA-B*35:03	4	12	9	DPATRPRTL	0.20
HLA-B*38:01	117	125	9	RHVNANKRF	0.20
HLA-B*44:03	113	125	13	EEKERHVNANKRF	0.20
HLA-B*51:01	120	128	9	NANKRFHNI	0.21
HLA-A*11:01	85	94	10	GKTLEERVKK	0.21
HLA-B*40:02	97	105	9	SEITIRCII	0.21
HLA-B*40:01	97	105	9	SEITIRCII	0.22
HLA-B*40:01	2	12	11	FEDPATRPRTL	0.22
HLA-C*07:02	76	83	8	YRHYQYSL	0.22
HLA-A*24:02	80	88	9	QYSLYGKTL	0.23
HLA-A*30:02	46	54	9	LFTDLRIVY	0.23
HLA-C*01:02	18	26	9	VLEESVHEI	0.24
HLA-B*44:03	115	125	11	KERHVNANKRF	0.24
HLA-B*44:03	74	81	8	SEYRHYQY	0.24
HLA-A*26:01	41	54	14	EVYKFLFTDLRIVY	0.25
HLA-C*08:01	18	26	9	VLEESVHEI	0.25
HLA-A*33:03	1	10	10	MFEDPATRPR	0.25
HLA-B*15:01	51	60	10	RIVYRDNNPY	0.25
HLA-B*44:02	115	125	11	KERHVNANKRF	0.25
HLA-C*04:01	1	12	12	MFEDPATRPRTL	0.25
HLA-C*07:02	140	148	9	WRPRPVTQV	0.26
HLA-A*30:01	6	15	10	ATRPRTLHEL	0.27
HLA-B*44:03	20	28	9	EESVHEIRL	0.27
HLA-C*04:01	53	62	10	VYRDNNPYGV	0.27
HLA-A*26:01	52	60	9	IVYRDNNPY	0.28
HLA-B*44:02	74	81	8	SEYRHYQY	0.28
HLA-C*07:02	75	83	9	EYRHYQYSL	0.28
HLA-C*07:02	38	46	9	QRREVYKFL	0.28
HLA-B*51:01	4	12	9	DPATRPRTL	0.29
HLA-B*13:02	97	105	9	SEITIRCII	0.30
HLA-A*01:01	73	84	12	ISEYRHYQYSLY	0.30
HLA-A*24:02	42	50	9	VYKFLFTDL	0.30

HLA-B*15:25	45	54	10	FLFTDLRIVY	0.31
HLA-B*44:02	20	28	9	EESVHEIRL	0.31
HLA-B*44:02	34	43	10	KKELQRREVY	0.31
HLA-C*04:01	18	26	9	VLEESVHEI	0.31
HLA-A*30:02	76	84	9	YRHYQYSLY	0.32
HLA-B*15:25	51	60	10	RIVYRDNNPY	0.33
HLA-B*52:01	97	105	9	SEITIRCII	0.34
HLA-A*11:01	47	55	9	FTDLRIVYR	0.35
HLA-C*06:02	68	76	9	RFLSKISEY	0.35
HLA-C*07:02	46	54	9	LFTDLRIVY	0.35
HLA-A*33:03	78	86	9	HYQYSLYGK	0.36
HLA-B*44:03	34	43	10	KKELQRREVY	0.36
HLA-A*24:02	124	132	9	RFHNIMGRW	0.37
HLA-B*13:02	79	88	10	YQYSLYGKTL	0.37
HLA-A*33:03	82	91	10	SLYGKTLEER	0.38
HLA-B*15:02	37	45	9	LQRREVYKF	0.38
HLA-B*15:02	45	54	10	FLFTDLRIVY	0.38
HLA-B*38:01	7	15	9	TRPRTLHEL	0.38
HLA-B*15:01	45	54	10	FLFTDLRIVY	0.39
HLA-B*51:01	94	101	8	KPLSEITI	0.39
HLA-A*02:01	82	92	11	SLYGKTLEERV	0.40
HLA-B*35:03	58	65	8	NPYGVCIM	0.40
HLA-B*40:01	20	28	9	EESVHEIRL	0.40
HLA-B*52:01	97	104	8	SEITIRCI	0.40
HLA-C*06:02	67	76	10	LRFLSKISEY	0.40
HLA-B*38:01	79	88	10	YQYSLYGKTL	0.41
HLA-B*38:01	2	12	11	FEDPATRPRTL	0.42
HLA-B*46:01	45	54	10	FLFTDLRIVY	0.42
HLA-B*46:01	46	54	9	LFTDLRIVY	0.42
HLA-B*58:02	73	81	9	ISEYRHYQY	0.43
HLA-A*01:01	47	55	9	FTDLRIVYR	0.43
HLA-A*24:02	75	83	9	EYRHYQYSL	0.44
HLA-B*38:01	54	65	12	YRDNNPYGVCIM	0.44
HLA-B*46:01	91	99	9	RVKKPLSEI	0.44
HLA-A*33:03	68	77	10	RFLSKISEYR	0.45
HLA-B*15:25	68	76	9	RFLSKISEY	0.45
HLA-B*15:01	71	79	9	SKISEYRHY	0.46
HLA-B*15:02	69	76	8	FLSKISEY	0.46
HLA-B*51:01	58	65	8	NPYGVCIM	0.47
HLA-B*44:03	74	84	11	SEYRHYQYSLY	0.48
HLA-B*40:01	19	28	10	LEESVHEIRL	0.49
HLA-B*40:02	2	12	11	FEDPATRPRTL	0.49
HLA-B*15:25	35	43	9	KELQRREVY	0.50

HLA-B*15:01	35	43	9	KELQRREVY	0.51
HLA-B*38:01	39	47	9	RREVYKFLF	0.51
HLA-C*04:03	54	62	9	YRDNNPYGV	0.51
HLA-A*01:01	72	81	10	KISEYRHYQY	0.52
HLA-A*02:06	10	18	9	RTLHELCEV	0.52
HLA-A*30:01	68	76	9	RFLSKISEY	0.52
HLA-B*15:02	35	43	9	KELQRREVY	0.52
HLA-A*11:01	82	94	13	SLYGKTLEERVKK	0.53
HLA-B*13:01	18	26	9	VLEESVHEI	0.53
HLA-B*58:02	124	132	9	RFHNIMGRW	0.53
HLA-A*24:02	44	52	9	KFLFTDLRI	0.54
HLA-B*44:02	74	84	11	SEYRHYQYSLY	0.54
HLA-C*01:02	91	99	9	RVKKPLSEI	0.54
HLA-C*06:02	123	132	10	KRFHNIMGRW	0.54
HLA-A*24:02	79	88	10	YQYSLYGKTL	0.55
HLA-A*30:02	71	79	9	SKISEYRHY	0.55
HLA-B*13:01	2	12	11	FEDPATRPRTL	0.55
HLA-B*15:01	68	76	9	RFLSKISEY	0.55
HLA-B*15:02	46	54	9	LFTDLRIVY	0.55
HLA-B*44:02	35	45	11	KELQRREVYKF	0.55
HLA-B*44:03	35	45	11	KELQRREVYKF	0.55
HLA-B*15:25	69	76	8	FLSKISEY	0.56
HLA-C*06:02	38	47	10	QRREVYKFLF	0.56
HLA-A*30:02	67	76	10	LRFLSKISEY	0.57
HLA-C*08:01	54	62	9	YRDNNPYGV	0.57
HLA-B*38:01	54	67	14	YRDNNPYGVCIMCL	0.58
HLA-B*13:02	74	83	10	SEYRHYQYSL	0.59
HLA-B*35:01	46	54	9	LFTDLRIVY	0.59
HLA-B*44:03	74	83	10	SEYRHYQYSL	0.59
HLA-A*30:01	115	123	9	KERHVNANK	0.60
HLA-B*40:01	2	15	14	FEDPATRPRTLHEL	0.60
HLA-B*44:02	97	105	9	SEITIRCII	0.60
HLA-B*44:03	97	105	9	SEITIRCII	0.60
HLA-B*51:01	141	148	8	RPRPVTQV	0.60
HLA-C*06:02	80	88	9	QYSLYGKTL	0.60
HLA-B*40:02	20	28	9	EESVHEIRL	0.61
HLA-C*03:02	46	54	9	LFTDLRIVY	0.62
HLA-A*01:01	69	81	13	FLSKISEYRHYQY	0.62
HLA-B*15:01	69	76	8	FLSKISEY	0.62
HLA-B*38:01	54	64	11	YRDNNPYGVCI	0.62
HLA-C*04:01	80	88	9	QYSLYGKTL	0.62
HLA-A*01:01	71	81	11	SKISEYRHYQY	0.63
HLA-B*44:02	74	83	10	SEYRHYQYSL	0.63

HLA-B*52:01	19	26	8	LEESVHEI	0.63
HLA-B*52:01	120	128	9	NANKRFHNI	0.64
HLA-B*52:01	37	45	9	LQRREVYKF	0.64
HLA-C*04:01	68	76	9	RFLSKISEY	0.64
HLA-C*07:02	117	125	9	RHVNANKRF	0.64
HLA-B*15:25	71	79	9	SKISEYRHY	0.65
HLA-B*13:01	37	45	9	LQRREVYKF	0.66
HLA-B*46:01	47	54	8	FTDLRIVY	0.66
HLA-B*46:01	68	76	9	RFLSKISEY	0.67
HLA-B*58:01	124	132	9	RFHNIMGRW	0.68
HLA-B*13:01	74	83	10	SEYRHYQYSL	0.68
HLA-C*06:02	46	54	9	LFTDLRIVY	0.68
HLA-A*11:01	52	60	9	IVYRDNNPY	0.70
HLA-B*40:01	88	96	9	LEERVKKPL	0.70
HLA-B*51:01	111	119	9	CPEEKERHV	0.71
HLA-B*58:02	52	60	9	IVYRDNNPY	0.71
HLA-B*58:02	123	132	10	KRFHNIMGRW	0.71
HLA-C*04:01	46	54	9	LFTDLRIVY	0.71
HLA-A*26:01	45	54	10	FLFTDLRIVY	0.72
HLA-B*15:02	68	76	9	RFLSKISEY	0.72
HLA-B*40:02	40	47	8	REVYKFLF	0.72
HLA-B*58:01	52	60	9	IVYRDNNPY	0.73
HLA-A*11:01	82	91	10	SLYGKTLEER	0.74
HLA-A*30:02	77	84	8	RHYQYSLY	0.74
HLA-B*15:01	79	88	10	YQYSLYGKTL	0.74
HLA-A*11:01	82	93	12	SLYGKTLEERVK	0.75
HLA-B*13:02	18	26	9	VLEESVHEI	0.75
HLA-B*52:01	45	53	9	FLFTDLRIV	0.75
HLA-B*58:01	73	81	9	ISEYRHYQY	0.75
HLA-C*03:04	91	99	9	RVKKPLSEI	0.76
HLA-A*33:03	1	8	8	MFEDPATR	0.76
HLA-C*03:02	91	99	9	RVKKPLSEI	0.76
HLA-A*01:01	52	60	9	IVYRDNNPY	0.77
HLA-A*33:03	42	51	10	VYKFLFTDLR	0.77
HLA-B*13:02	97	104	8	SEITIRCI	0.77
HLA-B*15:02	51	60	10	RIVYRDNNPY	0.77
HLA-B*44:03	40	47	8	REVYKFLF	0.77
HLA-B*44:03	71	79	9	SKISEYRHY	0.77
HLA-C*07:02	79	88	10	YQYSLYGKTL	0.77
HLA-A*30:02	72	79	8	KISEYRHY	0.78
HLA-B*40:02	97	104	8	SEITIRCI	0.78
HLA-B*46:01	69	76	8	FLSKISEY	0.78
HLA-B*58:02	91	99	9	RVKKPLSEI	0.78

HLA-B*15:02	71	79	9	SKISEYRHY	0.79
HLA-B*35:03	2	12	11	FEDPATRPRTL	0.80
HLA-B*44:02	40	47	8	REVYKFLF	0.80
HLA-C*04:03	1	12	12	MFEDPATRPRTL	0.80
HLA-A*30:01	72	80	9	KISEYRHYQ	0.81
HLA-A*30:02	70	79	10	LSKISEYRHY	0.81
HLA-A*33:03	36	44	9	ELQRREVYK	0.81
HLA-C*08:01	45	53	9	FLFTDLRIV	0.81
HLA-B*44:02	32	43	12	QCKKELQRREVY	0.82
HLA-C*07:02	67	76	10	LRFLSKISEY	0.82
HLA-A*02:01	45	52	8	FLFTDLRI	0.83
HLA-B*38:01	116	125	10	ERHVNANKRF	0.83
HLA-C*04:01	39	47	9	RREVYKFLF	0.83
HLA-B*15:01	72	81	10	KISEYRHYQY	0.84
HLA-B*35:03	58	67	10	NPYGVCIMCL	0.85
HLA-B*52:01	91	99	9	RVKKPLSEI	0.85
HLA-B*38:01	2	15	14	FEDPATRPRTLHEL	0.86
HLA-B*38:01	76	83	8	YRHYQYSL	0.86
HLA-C*03:02	68	76	9	RFLSKISEY	0.86
HLA-A*24:02	53	62	10	VYRDNNPYGV	0.87
HLA-A*26:01	49	60	12	DLRIVYRDNNPY	0.87
HLA-B*15:25	79	88	10	YQYSLYGKTL	0.87
HLA-C*04:01	44	52	9	KFLFTDLRI	0.87
HLA-A*02:01	11	19	9	TLHELCEVL	0.88
HLA-B*13:01	97	105	9	SEITIRCII	0.88
HLA-B*35:01	47	54	8	FTDLRIVY	0.88
HLA-C*06:02	76	88	13	YRHYQYSLYGKTL	0.88
HLA-A*24:02	68	76	9	RFLSKISEY	0.89
HLA-A*30:02	44	54	11	KFLFTDLRIVY	0.89
HLA-B*46:01	37	45	9	LQRREVYKF	0.89
HLA-C*03:02	45	54	10	FLFTDLRIVY	0.89
HLA-A*26:01	76	84	9	YRHYQYSLY	0.90
HLA-B*40:02	19	28	10	LEESVHEIRL	0.91
HLA-C*04:01	47	55	9	FTDLRIVYR	0.91
HLA-B*38:01	140	148	9	WRPRPVTQV	0.92
HLA-A*30:02	35	43	9	KELQRREVY	0.93
HLA-B*15:01	46	54	9	LFTDLRIVY	0.93
HLA-B*44:02	71	79	9	SKISEYRHY	0.93
HLA-B*13:02	19	26	8	LEESVHEI	0.94
HLA-B*15:02	47	54	8	FTDLRIVY	0.94
HLA-A*30:02	91	99	9	RVKKPLSEI	0.95
HLA-A*01:01	69	79	11	FLSKISEYRHY	0.96
HLA-A*26:01	47	54	8	FTDLRIVY	0.96

HLA-B*15:25	46	54	9	LFTDLRIVY	0.96
HLA-B*15:25	72	81	10	KISEYRHYQY	0.96
HLA-B*51:01	58	67	10	NPYGVCIMCL	0.96
HLA-C*03:04	81	88	8	YSLYGKTL	0.96
HLA-C*04:03	45	53	9	FLFTDLRIV	0.96
HLA-C*07:02	42	50	9	VYKFLFTDL	0.96
HLA-A*30:02	68	79	12	RFLSKISEYRHY	0.97
HLA-A*30:02	68	77	10	RFLSKISEYR	0.97
HLA-C*03:02	79	88	10	YQYSLYGKTL	0.97
HLA-B*40:02	88	96	9	LEERVKKPL	0.98
HLA-B*13:01	20	28	9	EESVHEIRL	0.99
HLA-B*15:02	74	81	8	SEYRHYQY	0.99
HLA-B*38:01	38	46	9	QRREVYKFL	0.99
HLA-B*52:01	18	26	9	VLEESVHEI	0.99
HLA-C*06:02	75	83	9	EYRHYQYSL	0.99
HLA-A*02:06	54	62	9	YRDNNPYGV	1.00
HLA-B*35:01	4	12	9	DPATRPRTL	1.00

Note: A low percentile rank (PR) showed a good binder, and only epitopes with PR⩽1.00 were selected.

Table S4 Prediction results for HPV52 E6 variant sequence HLA-I epitope peptides

allele	start	end	length	peptide	percentile rank (PR)
HLA-A*33:03	127	135	9	NIMGRWTGR	0.01
HLA-A*30:01	91	99	9	RVRKPLSEI	0.01
HLA-C*06:02	76	84	9	YRHYQYSLY	0.02
HLA-A*11:01	86	94	9	KTLEERVRK	0.02
HLA-A*30:01	86	94	9	KTLEERVRK	0.03
HLA-A*30:02	68	76	9	RFLSKISEY	0.04
HLA-B*38:01	54	62	9	YRDNNPYGV	0.04
HLA-B*15:25	52	60	9	IVYRDNNPY	0.04
HLA-A*01:01	73	81	9	ISEYRHYQY	0.04
HLA-B*44:02	35	43	9	KELQRREVY	0.05
HLA-B*44:03	35	43	9	KELQRREVY	0.05
HLA-B*15:01	37	45	9	LQRREVYKF	0.05
HLA-B*15:02	52	60	9	IVYRDNNPY	0.05
HLA-A*01:01	47	60	14	FTDLRIVYRDNNPY	0.05
HLA-A*01:01	47	54	8	FTDLRIVY	0.05
HLA-C*06:02	54	62	9	YRDNNPYGV	0.05
HLA-C*07:02	76	84	9	YRHYQYSLY	0.06
HLA-A*02:01	18	26	9	VLEESVHEI	0.06
HLA-B*15:01	52	60	9	IVYRDNNPY	0.06
HLA-C*04:03	18	26	9	VLEESVHEI	0.07
HLA-A*02:01	45	53	9	FLFTDLRIV	0.07
HLA-A*30:02	52	60	9	IVYRDNNPY	0.07
HLA-A*33:03	47	55	9	FTDLRIVYR	0.07
HLA-C*07:02	54	62	9	YRDNNPYGV	0.07
HLA-C*06:02	38	46	9	QRREVYKFL	0.08
HLA-A*33:03	1	10	10	MFQDPATRPR	0.09
HLA-B*15:25	37	45	9	LQRREVYKF	0.09
HLA-C*06:02	7	15	9	TRPRTLHEL	0.09
HLA-B*46:01	52	60	9	IVYRDNNPY	0.1
HLA-A*02:06	45	53	9	FLFTDLRIV	0.1
HLA-A*30:01	6	14	9	ATRPRTLHE	0.1
HLA-A*33:03	69	77	9	FLSKISEYR	0.11
HLA-A*33:03	46	55	10	LFTDLRIVYR	0.11
HLA-C*07:02	68	76	9	RFLSKISEY	0.11
HLA-C*07:02	7	15	9	TRPRTLHEL	0.12
HLA-A*30:02	73	81	9	ISEYRHYQY	0.13
HLA-C*06:02	140	148	9	WRPRPVTQV	0.13
HLA-C*06:02	76	83	8	YRHYQYSL	0.14
HLA-C*03:02	52	60	9	IVYRDNNPY	0.15
HLA-B*35:01	52	60	9	IVYRDNNPY	0.15
HLA-A*02:06	18	26	9	VLEESVHEI	0.15

HLA-C*07:02	39	47	9	RREVYKFLF	0.15
HLA-C*04:01	54	62	9	YRDNNPYGV	0.16
HLA-B*52:01	79	88	10	YQYSLYGKTL	0.17
HLA-C*06:02	39	47	9	RREVYKFLF	0.17
HLA-B*40:02	74	83	10	SEYRHYQYSL	0.18
HLA-B*40:01	74	83	10	SEYRHYQYSL	0.18
HLA-A*30:02	72	81	10	KISEYRHYQY	0.18
HLA-A*33:03	126	135	10	HNIMGRWTGR	0.18
HLA-B*44:02	113	125	13	EEKERHVNANKRF	0.18
HLA-C*07:02	80	88	9	QYSLYGKTL	0.18
HLA-A*30:02	51	60	10	RIVYRDNNPY	0.19
HLA-B*13:01	79	88	10	YQYSLYGKTL	0.2
HLA-B*35:03	4	12	9	DPATRPRTL	0.2
HLA-B*38:01	117	125	9	RHVNANKRF	0.2
HLA-B*44:03	113	125	13	EEKERHVNANKRF	0.2
HLA-B*51:01	120	128	9	NANKRFHNI	0.21
HLA-B*40:02	97	105	9	SEITIRCII	0.21
HLA-B*40:01	97	105	9	SEITIRCII	0.22
HLA-C*07:02	76	83	8	YRHYQYSL	0.22
HLA-A*24:02	80	88	9	QYSLYGKTL	0.23
HLA-A*11:01	85	94	10	GKTLEERVRK	0.23
HLA-A*30:02	46	54	9	LFTDLRIVY	0.23
HLA-C*01:02	18	26	9	VLEESVHEI	0.24
HLA-B*44:03	115	125	11	KERHVNANKRF	0.24
HLA-B*44:03	74	81	8	SEYRHYQY	0.24
HLA-A*26:01	41	54	14	EVYKFLFTDLRIVY	0.25
HLA-C*08:01	18	26	9	VLEESVHEI	0.25
HLA-B*15:01	51	60	10	RIVYRDNNPY	0.25
HLA-B*44:02	115	125	11	KERHVNANKRF	0.25
HLA-C*07:02	140	148	9	WRPRPVTQV	0.26
HLA-A*30:01	6	15	10	ATRPRTLHEL	0.27
HLA-B*44:03	20	28	9	EESVHEIRL	0.27
HLA-C*04:01	53	62	10	VYRDNNPYGV	0.27
HLA-A*26:01	52	60	9	IVYRDNNPY	0.28
HLA-B*44:02	74	81	8	SEYRHYQY	0.28
HLA-C*07:02	75	83	9	EYRHYQYSL	0.28
HLA-C*07:02	38	46	9	QRREVYKFL	0.28
HLA-B*51:01	4	12	9	DPATRPRTL	0.29
HLA-B*13:02	97	105	9	SEITIRCII	0.3
HLA-A*01:01	73	84	12	ISEYRHYQYSLY	0.3
HLA-A*24:02	42	50	9	VYKFLFTDL	0.3
HLA-B*15:25	45	54	10	FLFTDLRIVY	0.31
HLA-B*44:02	20	28	9	EESVHEIRL	0.31

HLA-B*44:02	34	43	10	KKELQRREVY	0.31
HLA-C*04:01	18	26	9	VLEESVHEI	0.31
HLA-A*30:02	76	84	9	YRHYQYSLY	0.32
HLA-B*15:25	51	60	10	RIVYRDNNPY	0.33
HLA-B*52:01	97	105	9	SEITIRCII	0.34
HLA-C*01:02	91	99	9	RVRKPLSEI	0.34
HLA-A*11:01	47	55	9	FTDLRIVYR	0.35
HLA-B*38:01	2	12	11	FQDPATRPRTL	0.35
HLA-C*06:02	68	76	9	RFLSKISEY	0.35
HLA-C*07:02	46	54	9	LFTDLRIVY	0.35
HLA-A*33:03	78	86	9	HYQYSLYGK	0.36
HLA-A*33:03	1	8	8	MFQDPATR	0.36
HLA-B*44:03	34	43	10	KKELQRREVY	0.36
HLA-B*46:01	91	99	9	RVRKPLSEI	0.36
HLA-A*24:02	124	132	9	RFHNIMGRW	0.37
HLA-B*13:02	79	88	10	YQYSLYGKTL	0.37
HLA-A*33:03	82	91	10	SLYGKTLEER	0.38
HLA-B*15:02	37	45	9	LQRREVYKF	0.38
HLA-B*15:02	45	54	10	FLFTDLRIVY	0.38
HLA-B*38:01	7	15	9	TRPRTLHEL	0.38
HLA-C*04:03	2	12	11	FQDPATRPRTL	0.38
HLA-B*15:01	45	54	10	FLFTDLRIVY	0.39
HLA-B*51:01	94	101	8	KPLSEITI	0.39
HLA-A*02:01	82	92	11	SLYGKTLEERV	0.4
HLA-B*35:03	58	65	8	NPYGVCIM	0.4
HLA-B*40:01	20	28	9	EESVHEIRL	0.4
HLA-B*52:01	97	104	8	SEITIRCI	0.4
HLA-C*06:02	67	76	10	LRFLSKISEY	0.4
HLA-B*38:01	79	88	10	YQYSLYGKTL	0.41
HLA-B*46:01	45	54	10	FLFTDLRIVY	0.42
HLA-B*46:01	46	54	9	LFTDLRIVY	0.42
HLA-B*58:02	73	81	9	ISEYRHYQY	0.43
HLA-A*01:01	47	55	9	FTDLRIVYR	0.43
HLA-A*24:02	75	83	9	EYRHYQYSL	0.44
HLA-B*38:01	54	65	12	YRDNNPYGVCIM	0.44
HLA-A*33:03	68	77	10	RFLSKISEYR	0.45
HLA-B*15:25	68	76	9	RFLSKISEY	0.45
HLA-B*15:01	71	79	9	SKISEYRHY	0.46
HLA-B*15:02	69	76	8	FLSKISEY	0.46
HLA-C*04:03	2	15	14	FQDPATRPRTLHEL	0.46
HLA-B*51:01	58	65	8	NPYGVCIM	0.47
HLA-B*44:03	74	84	11	SEYRHYQYSLY	0.48
HLA-B*40:01	19	28	10	LEESVHEIRL	0.49

HLA-B*15:25	35	43	9	KELQRREVY	0.50
HLA-B*15:01	35	43	9	KELQRREVY	0.51
HLA-B*38:01	39	47	9	RREVYKFLF	0.51
HLA-C*04:03	54	62	9	YRDNNPYGV	0.51
HLA-A*01:01	72	81	10	KISEYRHYQY	0.52
HLA-A*02:06	10	18	9	RTLHELCEV	0.52
HLA-A*30:01	68	76	9	RFLSKISEY	0.52
HLA-B*15:02	35	43	9	KELQRREVY	0.52
HLA-B*13:01	18	26	9	VLEESVHEI	0.53
HLA-B*58:02	124	132	9	RFHNIMGRW	0.53
HLA-A*24:02	44	52	9	KFLFTDLRI	0.54
HLA-B*44:02	74	84	11	SEYRHYQYSLY	0.54
HLA-C*06:02	123	132	10	KRFHNIMGRW	0.54
HLA-A*11:01	82	94	13	SLYGKTLEERVRK	0.55
HLA-A*24:02	79	88	10	YQYSLYGKTL	0.55
HLA-A*30:02	71	79	9	SKISEYRHY	0.55
HLA-B*15:01	68	76	9	RFLSKISEY	0.55
HLA-B*15:02	46	54	9	LFTDLRIVY	0.55
HLA-B*44:02	35	45	11	KELQRREVYKF	0.55
HLA-B*44:03	35	45	11	KELQRREVYKF	0.55
HLA-B*15:25	69	76	8	FLSKISEY	0.56
HLA-C*06:02	38	47	10	QRREVYKFLF	0.56
HLA-A*30:02	67	76	10	LRFLSKISEY	0.57
HLA-C*04:01	1	12	12	MFQDPATRPRTL	0.57
HLA-C*08:01	54	62	9	YRDNNPYGV	0.57
HLA-B*38:01	54	67	14	YRDNNPYGVCIMCL	0.58
HLA-C*03:02	91	99	9	RVRKPLSEI	0.58
HLA-B*13:02	74	83	10	SEYRHYQYSL	0.59
HLA-B*35:01	46	54	9	LFTDLRIVY	0.59
HLA-B*44:03	74	83	10	SEYRHYQYSL	0.59
HLA-A*30:01	115	123	9	KERHVNANK	0.6
HLA-B*44:02	97	105	9	SEITIRCII	0.6
HLA-B*44:03	97	105	9	SEITIRCII	0.6
HLA-B*51:01	141	148	8	RPRPVTQV	0.6
HLA-C*06:02	80	88	9	QYSLYGKTL	0.6
HLA-B*40:02	20	28	9	EESVHEIRL	0.61
HLA-A*01:01	69	81	13	FLSKISEYRHYQY	0.62
HLA-B*15:01	69	76	8	FLSKISEY	0.62
HLA-B*38:01	54	64	11	YRDNNPYGVCI	0.62
HLA-C*03:02	46	54	9	LFTDLRIVY	0.62
HLA-C*04:01	80	88	9	QYSLYGKTL	0.62
HLA-C*04:01	2	12	11	FQDPATRPRTL	0.62
HLA-C*03:04	91	99	9	RVRKPLSEI	0.63

HLA-A*01:01	71	81	11	SKISEYRHYQY	0.63
HLA-B*44:02	74	83	10	SEYRHYQYSL	0.63
HLA-B*52:01	19	26	8	LEESVHEI	0.63
HLA-B*52:01	120	128	9	NANKRFHNI	0.64
HLA-B*52:01	37	45	9	LQRREVYKF	0.64
HLA-C*04:01	68	76	9	RFLSKISEY	0.64
HLA-C*07:02	117	125	9	RHVNANKRF	0.64
HLA-B*15:25	71	79	9	SKISEYRHY	0.65
HLA-B*52:01	91	99	9	RVRKPLSEI	0.65
HLA-C*06:02	92	101	10	VRKPLSEITI	0.65
HLA-B*13:01	37	45	9	LQRREVYKF	0.66
HLA-B*46:01	47	54	8	FTDLRIVY	0.66
HLA-B*46:01	68	76	9	RFLSKISEY	0.67
HLA-B*13:01	74	83	10	SEYRHYQYSL	0.68
HLA-B*58:01	124	132	9	RFHNIMGRW	0.68
HLA-B*58:02	91	99	9	RVRKPLSEI	0.68
HLA-C*06:02	46	54	9	LFTDLRIVY	0.68
HLA-B*40:01	88	96	9	LEERVRKPL	0.69
HLA-A*11:01	52	60	9	IVYRDNNPY	0.70
HLA-B*51:01	111	119	9	CPEEKERHV	0.71
HLA-B*58:02	52	60	9	IVYRDNNPY	0.71
HLA-B*58:02	123	132	10	KRFHNIMGRW	0.71
HLA-C*04:01	46	54	9	LFTDLRIVY	0.71
HLA-A*26:01	45	54	10	FLFTDLRIVY	0.72
HLA-B*15:02	68	76	9	RFLSKISEY	0.72
HLA-B*40:02	40	47	8	REVYKFLF	0.72
HLA-B*58:01	52	60	9	IVYRDNNPY	0.73
HLA-A*11:01	82	91	10	SLYGKTLEER	0.74
HLA-A*30:02	77	84	8	RHYQYSLY	0.74
HLA-B*15:01	79	88	10	YQYSLYGKTL	0.74
HLA-B*13:02	18	26	9	VLEESVHEI	0.75
HLA-B*52:01	45	53	9	FLFTDLRIV	0.75
HLA-B*58:01	73	81	9	ISEYRHYQY	0.75
HLA-A*01:01	52	60	9	IVYRDNNPY	0.77
HLA-A*33:03	42	51	10	VYKFLFTDLR	0.77
HLA-B*13:02	97	104	8	SEITIRCI	0.77
HLA-B*15:02	51	60	10	RIVYRDNNPY	0.77
HLA-B*44:03	40	47	8	REVYKFLF	0.77
HLA-B*44:03	71	79	9	SKISEYRHY	0.77
HLA-C*07:02	79	88	10	YQYSLYGKTL	0.77
HLA-A*30:02	72	79	8	KISEYRHY	0.78
HLA-B*40:02	97	104	8	SEITIRCI	0.78
HLA-B*46:01	69	76	8	FLSKISEY	0.78

HLA-C*08:01	2	12	11	FQDPATRPRTL	0.78
HLA-A*30:01	91	100	10	RVRKPLSEIT	0.79
HLA-B*15:02	71	79	9	SKISEYRHY	0.79
HLA-B*35:03	2	12	11	FQDPATRPRTL	0.8
HLA-B*44:02	40	47	8	REVYKFLF	0.8
HLA-A*30:01	72	80	9	KISEYRHYQ	0.81
HLA-A*30:02	70	79	10	LSKISEYRHY	0.81
HLA-A*33:03	36	44	9	ELQRREVYK	0.81
HLA-C*08:01	45	53	9	FLFTDLRIV	0.81
HLA-B*13:01	2	12	11	FQDPATRPRTL	0.82
HLA-B*44:02	32	43	12	QCKKELQRREVY	0.82
HLA-C*07:02	67	76	10	LRFLSKISEY	0.82
HLA-A*02:01	45	52	8	FLFTDLRI	0.83
HLA-B*38:01	116	125	10	ERHVNANKRF	0.83
HLA-C*04:01	39	47	9	RREVYKFLF	0.83
HLA-B*15:01	72	81	10	KISEYRHYQY	0.84
HLA-B*35:03	58	67	10	NPYGVCIMCL	0.85
HLA-B*38:01	76	83	8	YRHYQYSL	0.86
HLA-C*03:02	68	76	9	RFLSKISEY	0.86
HLA-A*24:02	53	62	10	VYRDNNPYGV	0.87
HLA-A*26:01	49	60	12	DLRIVYRDNNPY	0.87
HLA-B*15:25	79	88	10	YQYSLYGKTL	0.87
HLA-B*38:01	2	15	14	FQDPATRPRTLHEL	0.87
HLA-C*04:01	44	52	9	KFLFTDLRI	0.87
HLA-A*02:01	11	19	9	TLHELCEVL	0.88
HLA-B*13:01	97	105	9	SEITIRCII	0.88
HLA-B*35:01	47	54	8	FTDLRIVY	0.88
HLA-C*06:02	76	88	13	YRHYQYSLYGKTL	0.88
HLA-A*24:02	68	76	9	RFLSKISEY	0.89
HLA-A*30:02	44	54	11	KFLFTDLRIVY	0.89
HLA-B*46:01	37	45	9	LQRREVYKF	0.89
HLA-C*03:02	45	54	10	FLFTDLRIVY	0.89
HLA-A*26:01	76	84	9	YRHYQYSLY	0.9
HLA-B*40:02	19	28	10	LEESVHEIRL	0.91
HLA-C*04:01	47	55	9	FTDLRIVYR	0.91
HLA-B*38:01	140	148	9	WRPRPVTQV	0.92
HLA-A*30:02	35	43	9	KELQRREVY	0.93
HLA-B*15:01	46	54	9	LFTDLRIVY	0.93
HLA-B*44:02	71	79	9	SKISEYRHY	0.93
HLA-B*13:02	19	26	8	LEESVHEI	0.94
HLA-B*15:02	47	54	8	FTDLRIVY	0.94
HLA-A*30:02	91	99	9	RVRKPLSEI	0.95
HLA-A*01:01	69	79	11	FLSKISEYRHY	0.96

HLA-A*26:01	47	54	8	FTDLRIVY	0.96
HLA-B*15:25	46	54	9	LFTDLRIVY	0.96
HLA-B*15:25	72	81	10	KISEYRHYQY	0.96
HLA-B*51:01	58	67	10	NPYGVCIMCL	0.96
HLA-C*03:04	81	88	8	YSLYGKTL	0.96
HLA-C*04:03	45	53	9	FLFTDLRIV	0.96
HLA-C*07:02	42	50	9	VYKFLFTDL	0.96
HLA-A*30:02	68	79	12	RFLSKISEYRHY	0.97
HLA-A*30:02	68	77	10	RFLSKISEYR	0.97
HLA-C*03:02	79	88	10	YQYSLYGKTL	0.97
HLA-B*40:02	88	96	9	LEERVRKPL	0.98
HLA-B*13:01	20	28	9	EESVHEIRL	0.99
HLA-B*15:02	74	81	8	SEYRHYQY	0.99
HLA-B*38:01	38	46	9	QRREVYKFL	0.99
HLA-B*52:01	18	26	9	VLEESVHEI	0.99
HLA-C*06:02	75	83	9	EYRHYQYSL	0.99
HLA-A*02:06	54	62	9	YRDNNPYGV	1
HLA-B*35:01	4	12	9	DPATRPRTL	1
HLA-C*08:01	2	15	14	FQDPATRPRTLHEL	1

Note: A low percentile rank (PR) showed a good binder, and only epitopes with PR≤1.00 were selected.
