# Supplementary material for: The genetic variability, phylogeny and functional significance of E6, E7 and LCR in human papillomavirus type 52 isolates in Sichuan, China
Source: Virol J. 2021 May 3;18:94. doi: 10.1186/s12985-021-01565-5 (PMC8091156; doi:10.1186/s12985-021-01565-5)
Supplement: Supplementary file 3 — Additional file 3: Table S5. Prediction results for HPV-52 E7 reference sequence HLA-I epitope peptides. Table S6. Prediction results for HPV-52 E7 variant sequence HLA-I epitope peptides. [file 12985_2021_1565_MOESM3_ESM.pdf]

Table S5 Prediction results for HPV-52 E7 reference sequence HLA-I epitope peptides

allele	start	end	length	peptide	percentile rank (PR)
HLA-B*44:02	47	55	9	AEQATSNYY	0.01
HLA-B*44:03	47	55	9	AEQATSNYY	0.01
HLA-B*15:01	45	54	10	GQAEQATSNY	0.01
HLA-B*15:25	45	54	10	GQAEQATSNY	0.04
HLA-A*30:02	51	59	9	TSNYYIVTY	0.06
HLA-C*06:02	78	86	9	LRTLQQMLL	0.07
HLA-B*44:02	47	59	13	AEQATSNYYIVTY	0.08
HLA-B*44:02	46	55	10	QAEQATSNYY	0.08
HLA-B*44:03	46	55	10	QAEQATSNYY	0.09
HLA-B*44:03	47	59	13	AEQATSNYYIVTY	0.09
HLA-A*01:01	46	54	9	QAEQATSNY	0.10
HLA-A*01:01	51	59	9	TSNYYIVTY	0.11
HLA-A*26:01	51	59	9	TSNYYIVTY	0.12
HLA-B*35:01	46	54	9	QAEQATSNY	0.12
HLA-A*01:01	46	55	10	QAEQATSNYY	0.12
HLA-B*46:01	51	59	9	TSNYYIVTY	0.13
HLA-A*02:01	84	92	9	MLLGTLQVV	0.14
HLA-B*15:01	45	55	11	GQAEQATSNYY	0.14
HLA-A*02:06	84	92	9	MLLGTLQVV	0.16
HLA-B*13:01	48	56	9	EQATSNYYI	0.16
HLA-C*03:02	51	59	9	TSNYYIVTY	0.16
HLA-B*15:02	46	54	9	QAEQATSNY	0.17
HLA-A*30:02	50	59	10	ATSNYYIVTY	0.17
HLA-C*03:04	73	81	9	STATDLRTL	0.18
HLA-A*30:02	45	54	10	GQAEQATSNY	0.18
HLA-C*07:02	78	86	9	LRTLQQMLL	0.19
HLA-B*58:02	51	59	9	TSNYYIVTY	0.20
HLA-B*58:01	51	59	9	TSNYYIVTY	0.21
HLA-B*38:01	78	86	9	LRTLQQMLL	0.21
HLA-B*15:02	51	59	9	TSNYYIVTY	0.21
HLA-B*15:25	51	59	9	TSNYYIVTY	0.21
HLA-B*35:01	51	59	9	TSNYYIVTY	0.22
HLA-B*44:03	47	54	8	AEQATSNY	0.22
HLA-A*02:06	11	19	9	YILDLQPET	0.23
HLA-B*44:02	47	54	8	AEQATSNY	0.23
HLA-A*02:01	83	91	9	QMLLGTLQV	0.24
HLA-B*15:01	51	59	9	TSNYYIVTY	0.24
HLA-B*13:02	48	56	9	EQATSNYYI	0.25
HLA-A*01:01	50	59	10	ATSNYYIVTY	0.25
HLA-B*15:02	47	55	9	AEQATSNYY	0.25
HLA-B*44:02	47	56	10	AEQATSNYYI	0.26

HLA-B*15:02	45	54	10	GQAEQATSNY	0.27
HLA-B*44:03	47	56	10	AEQATSNYYI	0.27
HLA-A*02:06	83	91	9	QMLLGTLQV	0.30
HLA-B*46:01	73	81	9	STATDLRTL	0.30
HLA-A*01:01	12	25	14	ILDLQPETTDLHCY	0.31
HLA-B*15:25	45	55	11	GQAEQATSNYY	0.31
HLA-A*26:01	50	59	10	ATSNYYIVTY	0.33
HLA-C*03:02	73	81	9	STATDLRTL	0.33
HLA-A*11:01	50	59	10	ATSNYYIVTY	0.35
HLA-A*30:02	47	55	9	AEQATSNYY	0.35
HLA-B*13:02	47	56	10	AEQATSNYYI	0.35
HLA-C*01:02	73	81	9	STATDLRTL	0.36
HLA-A*30:02	46	54	9	QAEQATSNY	0.37
HLA-B*15:25	47	55	9	AEQATSNYY	0.38
HLA-B*44:03	45	55	11	GQAEQATSNYY	0.38
HLA-A*01:01	45	54	10	GQAEQATSNY	0.39
HLA-B*52:01	48	56	9	EQATSNYYI	0.40
HLA-B*15:01	47	55	9	AEQATSNYY	0.40
HLA-B*38:01	48	56	9	EQATSNYYI	0.40
HLA-B*44:02	45	55	11	GQAEQATSNYY	0.40
HLA-A*02:01	11	19	9	YILDLQPET	0.42
HLA-B*44:03	16	25	10	QPETTDLHCY	0.42
HLA-A*30:02	2	11	10	RGDKATIKDY	0.44
HLA-B*13:01	47	55	9	AEQATSNYY	0.45
HLA-B*15:02	48	55	8	EQATSNYY	0.45
HLA-A*30:02	45	55	11	GQAEQATSNYY	0.47
HLA-A*01:01	46	59	14	QAEQATSNYYIVTY	0.49
HLA-B*44:02	16	25	10	QPETTDLHCY	0.49
HLA-A*33:03	90	98	9	QVVCPGCAR	0.50
HLA-A*11:01	51	59	9	TSNYYIVTY	0.50
HLA-B*35:01	16	25	10	QPETTDLHCY	0.53
HLA-B*44:02	42	55	14	RPDGQAEQATSNYY	0.53
HLA-B*58:01	73	81	9	STATDLRTL	0.56
HLA-A*30:02	46	55	10	QAEQATSNYY	0.57
HLA-B*44:03	42	55	14	RPDGQAEQATSNYY	0.57
HLA-B*58:01	5	13	9	KATIKDYIL	0.57
HLA-C*01:02	7	15	9	TIKDYILDL	0.57
HLA-A*26:01	46	54	9	QAEQATSNY	0.58
HLA-B*35:01	42	55	14	RPDGQAEQATSNYY	0.58
HLA-B*44:03	17	25	9	PETTDLHCY	0.60
HLA-B*58:02	5	13	9	KATIKDYIL	0.6
HLA-A*01:01	75	84	10	ATDLRTLQQM	0.61
HLA-B*15:01	50	59	10	ATSNYYIVTY	0.61

HLA-B*58:02	73	81	9	STATDLRTL	0.61
HLA-B*40:01	47	56	10	AEQATSNYYI	0.62
HLA-B*51:01	49	57	9	QATSNYYIV	0.62
HLA-B*58:02	50	59	10	ATSNYYIVTY	0.67
HLA-C*08:01	73	81	9	STATDLRTL	0.68
HLA-B*35:01	42	54	13	RPDGQAEQATSNY	0.69
HLA-C*06:02	51	59	9	TSNYYIVTY	0.69
HLA-A*01:01	75	83	9	ATDLRTLQQ	0.71
HLA-B*15:25	46	54	9	QAEQATSNY	0.71
HLA-B*15:25	50	59	10	ATSNYYIVTY	0.72
HLA-A*01:01	2	11	10	RGDKATIKDY	0.73
HLA-C*03:04	5	13	9	KATIKDYIL	0.73
HLA-B*58:01	50	59	10	ATSNYYIVTY	0.74
HLA-B*15:02	48	59	12	EQATSNYYIVTY	0.75
HLA-B*40:01	47	55	9	AEQATSNYY	0.75
HLA-A*01:01	42	54	13	RPDGQAEQATSNY	0.76
HLA-C*03:02	61	69	9	HSCDSTLRL	0.76
HLA-C*08:01	5	13	9	KATIKDYIL	0.76
HLA-B*44:02	17	25	9	PETTDLHCY	0.78
HLA-B*58:02	61	69	9	HSCDSTLRL	0.78
HLA-C*04:03	12	22	11	ILDLQPETTDL	0.79
HLA-B*44:03	3	11	9	GDKATIKDY	0.81
HLA-B*44:02	3	11	9	GDKATIKDY	0.82
HLA-B*13:01	47	56	10	AEQATSNYYI	0.84
HLA-B*35:01	46	55	10	QAEQATSNYY	0.84
HLA-A*01:01	42	55	14	RPDGQAEQATSNYY	0.85
HLA-A*02:01	84	91	8	MLLGTLQV	0.85
HLA-A*26:01	73	81	9	STATDLRTL	0.86
HLA-B*44:02	44	55	12	DGQAEQATSNYY	0.86
HLA-B*58:01	61	69	9	HSCDSTLRL	0.86
HLA-C*03:04	7	15	9	TIKDYILDL	0.86
HLA-A*01:01	44	54	11	DGQAEQATSNY	0.87
HLA-A*26:01	7	15	9	TIKDYILDL	0.87
HLA-B*15:02	46	55	10	QAEQATSNYY	0.87
HLA-A*02:06	6	15	10	ATIKDYILDL	0.88
HLA-B*46:01	46	54	9	QAEQATSNY	0.88
HLA-B*38:01	4	12	9	DKATIKDYI	0.89
HLA-B*44:03	44	55	12	DGQAEQATSNYY	0.89
HLA-C*06:02	1	8	8	MRGDKATI	0.89
HLA-A*01:01	47	55	9	AEQATSNYY	0.90
HLA-C*01:02	5	13	9	KATIKDYIL	0.91
HLA-B*15:01	46	54	9	QAEQATSNY	0.94
HLA-C*04:03	12	20	9	ILDLQPETT	0.95

HLA-B*15:01	48	55	8	EQATSNYY	0.96
HLA-B*46:01	45	54	10	GQAEQATSNY	0.96
HLA-B*58:02	79	86	8	RTLQQMLL	0.97
HLA-B*40:02	47	56	10	AEQATSNYYI	0.98
HLA-B*13:02	83	91	9	QMLLGTLQV	0.98
HLA-A*26:01	18	25	8	ETTDLHCY	0.99
HLA-B*13:02	82	91	10	QQMLLGTLQV	0.99

Note: A low percentile rank (PR) showed a good binder, and only epitopes with PR≤1.00 were selected.

Table S6 Prediction results for HPV-52 E7 variant sequence HLA-I epitope peptides

allele	start	end	length	peptide	percentile rank (PR)
HLA-B*44:02	47	55	9	AEQATDNYY	0.01
HLA-B*44:03	47	55	9	AEQATDNYY	0.01
HLA-A*01:01	50	61	12	ATDNYYIVTDCY	0.05
HLA-B*15:01	45	54	10	GQAEQATDNY	0.05
HLA-C*06:02	78	86	9	LRTLQQMLL	0.07
HLA-A*01:01	46	54	9	QAEQATDNY	0.09
HLA-A*01:01	46	55	10	QAEQATDNYY	0.11
HLA-B*13:01	48	56	9	EQATDNYYI	0.12
HLA-A*02:01	84	92	9	MLLGTLQVV	0.14
HLA-B*15:25	45	54	10	GQAEQATDNY	0.14
HLA-A*02:06	84	92	9	MLLGTLQVV	0.16
HLA-B*44:03	46	55	10	QAEQATDNYY	0.16
HLA-C*03:04	73	81	9	STATDLRTL	0.18
HLA-C*07:02	78	86	9	LRTLQQMLL	0.19
HLA-B*35:01	46	54	9	QAEQATDNY	0.20
HLA-B*44:02	46	55	10	QAEQATDNYY	0.20
HLA-B*38:01	78	86	9	LRTLQQMLL	0.21
HLA-A*33:03	91	99	9	VVCPGCARR	0.23
HLA-A*02:06	11	19	9	YILDLQPET	0.23
HLA-A*02:01	83	91	9	QMLLGTLQV	0.24
HLA-B*13:02	48	56	9	EQATDNYYI	0.27
HLA-B*13:02	47	56	10	AEQATDNYYI	0.27
HLA-B*46:01	73	81	9	STATDLRTL	0.30
HLA-A*02:06	83	91	9	QMLLGTLQV	0.30
HLA-A*01:01	12	25	14	ILDLQPETTDLHCY	0.31
HLA-B*38:01	48	56	9	EQATDNYYI	0.32
HLA-A*30:02	45	54	10	GQAEQATDNY	0.33
HLA-C*03:02	73	81	9	STATDLRTL	0.33
HLA-B*44:02	47	56	10	AEQATDNYYI	0.33
HLA-B*44:03	47	54	8	AEQATDNY	0.33
HLA-A*01:01	45	54	10	GQAEQATDNY	0.34
HLA-C*01:02	73	81	9	STATDLRTL	0.36
HLA-B*15:01	45	55	11	GQAEQATDNYY	0.38
HLA-B*44:02	47	54	8	AEQATDNY	0.38
HLA-B*44:03	47	56	10	AEQATDNYYI	0.38
HLA-B*51:01	49	57	9	QATDNYYIV	0.39
HLA-A*02:01	11	19	9	YILDLQPET	0.42
HLA-B*44:03	16	25	10	QPETTDLHCY	0.42
HLA-A*30:02	2	11	10	RGDKATIKDY	0.44
HLA-B*13:01	47	56	10	AEQATDNYYI	0.45
HLA-B*44:02	16	25	10	QPETTDLHCY	0.49

HLA-B*15:02	47	55	9	AEQATDNYY	0.50
HLA-A*33:03	90	98	9	QVVCPGCAR	0.50
HLA-B*44:03	45	55	11	GQAEQATDNYY	0.51
HLA-B*35:01	16	25	10	QPETTDLHCY	0.53
HLA-B*58:01	73	81	9	STATDLRTL	0.56
HLA-B*40:01	47	56	10	AEQATDNYYI	0.57
HLA-B*58:01	5	13	9	KATIKDYIL	0.57
HLA-C*01:02	7	15	9	TIKDYILDL	0.57
HLA-C*08:01	49	57	9	QATDNYYIV	0.58
HLA-B*35:01	42	55	14	RPDGQAEQATDNYY	0.59
HLA-B*58:02	5	13	9	KATIKDYIL	0.60
HLA-B*44:03	17	25	9	PETTDLHCY	0.60
HLA-C*03:02	61	69	9	YSCNSTLRL	0.60
HLA-A*01:01	75	84	10	ATDLRTLQQM	0.61
HLA-A*30:02	45	55	11	GQAEQATDNYY	0.61
HLA-A*33:03	90	99	10	QVVCPGCARR	0.61
HLA-B*58:02	73	81	9	STATDLRTL	0.61
HLA-B*44:03	42	55	14	RPDGQAEQATDNYY	0.64
HLA-B*13:01	47	55	9	AEQATDNYY	0.65
HLA-B*44:02	45	55	11	GQAEQATDNYY	0.65
HLA-C*07:02	53	61	9	NYYIVTDCY	0.65
HLA-A*30:02	53	61	9	NYYIVTDCY	0.66
HLA-B*15:02	45	54	10	GQAEQATDNY	0.66
HLA-B*15:25	45	55	11	GQAEQATDNYY	0.66
HLA-C*03:04	49	57	9	QATDNYYIV	0.66
HLA-A*01:01	42	54	13	RPDGQAEQATDNY	0.67
HLA-A*01:01	49	61	13	QATDNYYIVTDCY	0.67
HLA-A*30:02	47	55	9	AEQATDNYY	0.67
HLA-C*08:01	73	81	9	STATDLRTL	0.68
HLA-B*44:02	42	55	14	RPDGQAEQATDNYY	0.70
HLA-A*01:01	75	83	9	ATDLRTLQQ	0.71
HLA-B*52:01	48	56	9	EQATDNYYI	0.72
HLA-A*01:01	2	11	10	RGDKATIKDY	0.73
HLA-C*03:04	5	13	9	KATIKDYIL	0.73
HLA-C*03:04	61	69	9	YSCNSTLRL	0.73
HLA-B*15:02	46	54	9	QAEQATDNY	0.74
HLA-C*08:01	5	13	9	KATIKDYIL	0.76
HLA-B*15:01	47	55	9	AEQATDNYY	0.77
HLA-A*01:01	50	58	9	ATDNYYIVT	0.78
HLA-B*15:02	48	55	8	EQATDNYY	0.78
HLA-B*15:25	47	55	9	AEQATDNYY	0.78
HLA-B*44:02	17	25	9	PETTDLHCY	0.78
HLA-C*08:01	61	69	9	YSCNSTLRL	0.78

HLA-C*04:03	12	22	11	ILDLQPETTDL	0.79
HLA-A*30:02	46	55	10	QAEQATDNYY	0.79
HLA-C*06:02	53	61	9	NYYIVTDCY	0.79
HLA-B*44:03	3	11	9	GDKATIKDY	0.81
HLA-A*30:02	46	54	9	QAEQATDNY	0.82
HLA-B*44:02	3	11	9	GDKATIKDY	0.82
HLA-A*02:01	84	91	8	MLLGTLQV	0.85
HLA-B*35:01	46	55	10	QAEQATDNYY	0.85
HLA-A*26:01	73	81	9	STATDLRTL	0.86
HLA-C*03:04	7	15	9	TIKDYILDL	0.86
HLA-A*26:01	7	15	9	TIKDYILDL	0.87
HLA-A*02:06	6	15	10	ATIKDYILDL	0.88
HLA-B*35:01	42	54	13	RPDGQAEQATDNY	0.88
HLA-B*38:01	4	12	9	DKATIKDYI	0.89
HLA-C*06:02	1	8	8	MRGDKATI	0.89
HLA-B*40:02	47	56	10	AEQATDNYYI	0.90
HLA-A*01:01	42	55	14	RPDGQAEQATDNYY	0.91
HLA-C*01:02	5	13	9	KATIKDYIL	0.91
HLA-A*24:02	53	61	9	NYYIVTDCY	0.92
HLA-A*01:01	44	54	11	DGQAEQATDNY	0.95
HLA-C*04:03	12	20	9	ILDLQPETT	0.95
HLA-B*40:01	47	55	9	AEQATDNYY	0.96
HLA-B*58:02	61	69	9	YSCNSTLRL	0.96
HLA-C*01:02	61	69	9	YSCNSTLRL	0.96
HLA-B*58:02	79	86	8	RTLQQMLL	0.97
HLA-B*13:02	83	91	9	QMLLGTLQV	0.98
HLA-B*46:01	61	69	9	YSCNSTLRL	0.98
HLA-A*26:01	18	25	8	ETTDLHCY	0.99
HLA-B*13:02	82	91	10	QQMLLGTLQV	0.99
HLA-B*51:01	49	56	8	QATDNYYI	0.99

Note: A low percentile rank (PR) showed a good binder, and only epitopes with PR≤1.00 were selected.
