# Supplementary material for: The genetic variability, phylogeny and functional significance of E6, E7 and LCR in human papillomavirus type 52 isolates in Sichuan, China
Source: Virol J. 2021 May 3;18:94. doi: 10.1186/s12985-021-01565-5 (PMC8091156; doi:10.1186/s12985-021-01565-5)
Supplement: Supplementary file 4 — Additional file 4: Table S7. Prediction results for HPV-52 E6 reference sequence HLA-II epitope peptides. Table S8. Prediction results for HPV-52 E6 variant sequence HLA-II epitope peptides. [file 12985_2021_1565_MOESM4_ESM.pdf]

Table S7 Prediction results for HPV-52 E6 reference sequence HLA-II epitope peptides

allele	start	end	length	peptide	adjusted rank (AR)
HLA-DPA1*01:03/DPB1*04:01	42	54	13	VYKFLFTDLRIVY	0.08
HLA-DPA1*01:03/DPB1*04:01	41	54	14	EVYKFLFTDLRIVY	0.09
HLA-DPA1*01:03/DPB1*04:01	40	53	14	REVYKFLFTDLRIV	0.11
HLA-DPA1*01:03/DPB1*04:01	43	55	13	YKFLFTDLRIVYR	0.12
HLA-DPA1*01:03/DPB1*04:01	42	55	14	VYKFLFTDLRIVYR	0.13
HLA-DPA1*01:03/DPB1*04:01	41	53	13	EVYKFLFTDLRIV	0.14
HLA-DPA1*01:03/DPB1*04:01	39	53	15	RREVYKFLFTDLRIV	0.15
HLA-DPA1*01:03/DPB1*04:01	42	53	12	VYKFLFTDLRIV	0.15
HLA-DRB1*03:01	42	56	15	VYKFLFTDLRIVYRD	0.15
HLA-DPA1*01:03/DPB1*04:01	43	54	12	YKFLFTDLRIVY	0.15
HLA-DRB1*03:01	43	57	15	YKFLFTDLRIVYRDN	0.15
HLA-DRB1*03:01	41	55	15	EVYKFLFTDLRIVYR	0.17
HLA-DPA1*01:03/DPB1*04:01	40	54	15	REVYKFLFTDLRIVY	0.17
HLA-DPA1*01:03/DPB1*04:01	41	55	15	EVYKFLFTDLRIVYR	0.18
HLA-DPA1*01:03/DPB1*04:01	39	54	16	RREVYKFLFTDLRIVY	0.22
HLA-DRB1*03:01	41	56	16	EVYKFLFTDLRIVYRD	0.24
HLA-DRB1*03:01	40	55	16	REVYKFLFTDLRIVYR	0.24
HLA-DRB1*03:01	42	57	16	VYKFLFTDLRIVYRDN	0.24
HLA-DRB1*03:01	43	58	16	YKFLFTDLRIVYRDNN	0.24
HLA-DRB1*03:01	43	56	14	YKFLFTDLRIVYRD	0.26
HLA-DRB1*03:01	42	55	14	VYKFLFTDLRIVYR	0.27
HLA-DPA1*01:03/DPB1*04:01	38	53	16	QRREVYKFLFTDLRIV	0.28
HLA-DPA1*01:03/DPB1*04:01	40	55	16	REVYKFLFTDLRIVYR	0.28
HLA-DPA1*01:03/DPB1*04:01	42	56	15	VYKFLFTDLRIVYRD	0.28
HLA-DPA1*01:03/DPB1*04:01	43	56	14	YKFLFTDLRIVYRD	0.30
HLA-DRB1*03:01	44	58	15	KFLFTDLRIVYRDNN	0.32
HLA-DRB1*03:01	44	59	16	KFLFTDLRIVYRDNNP	0.33
HLA-DPA1*01:03/DPB1*04:01	41	56	16	EVYKFLFTDLRIVYRD	0.36
HLA-DPA1*01:03/DPB1*04:01	39	55	17	RREVYKFLFTDLRIVYR	0.37
HLA-DPA1*01:03/DPB1*04:01	39	52	14	RREVYKFLFTDLRI	0.38
HLA-DPA1*01:03/DPB1*04:01	38	54	17	QRREVYKFLFTDLRIVY	0.39
HLA-DRB1*03:01	44	57	14	KFLFTDLRIVYRDN	0.4
HLA-DRB1*03:01	41	57	17	EVYKFLFTDLRIVYRDN	0.41
HLA-DRB1*03:01	40	56	17	REVYKFLFTDLRIVYRD	0.41
HLA-DRB1*03:01	42	58	17	VYKFLFTDLRIVYRDNN	0.41
HLA-DRB1*03:01	43	59	17	YKFLFTDLRIVYRDNNP	0.41
HLA-DRB1*03:01	43	55	13	YKFLFTDLRIVYR	0.42
HLA-DRB1*03:01	39	55	17	RREVYKFLFTDLRIVYR	0.43
HLA-DPA1*01:03/DPB1*04:01	37	53	17	LQRREVYKFLFTDLRIV	0.44
HLA-DRB1*03:01	44	60	17	KFLFTDLRIVYRDNNPY	0.46
HLA-DPA1*01:03/DPB1*04:01	43	57	15	YKFLFTDLRIVYRDN	0.48

HLA-DRB1*03:01	44	56	13	KFLFTDLRIVYRD	0.50
HLA-DPA1*01:03/DPB1*04:01	42	57	16	VYKFLFTDLRIVYRDN	0.50
HLA-DPA1*01:03/DPB1*04:01	38	52	15	QRREVYKFLFTDLRI	0.52
HLA-DPA1*01:03/DPB1*04:01	40	52	13	REVYKFLFTDLRI	0.53
HLA-DPA1*01:03/DPB1*04:01	40	56	17	REVYKFLFTDLRIVYRD	0.55
HLA-DPA1*01:03/DPB1*04:01	44	55	12	KFLFTDLRIVYR	0.56
HLA-DRB1*03:01	44	55	12	KFLFTDLRIVYR	0.62
HLA-DRB4*01:01	21	34	14	ESVHEIRLQCVQCK	0.66
HLA-DPA1*01:03/DPB1*04:01	41	57	17	EVYKFLFTDLRIVYRDN	0.70
HLA-DPA1*01:03/DPB1*04:01	37	52	16	LQRREVYKFLFTDLRI	0.80
HLA-DPA1*01:03/DPB1*04:01	38	55	18	QRREVYKFLFTDLRIVYR	0.80
HLA-DPA1*01:03/DPB1*04:01	44	56	13	KFLFTDLRIVYRD	0.81
HLA-DPA1*03:01/DPB1*04:02	40	53	14	REVYKFLFTDLRIV	0.82
HLA-DPA1*01:03/DPB1*04:01	38	51	14	QRREVYKFLFTDLR	0.83
HLA-DPA1*01:03/DPB1*04:01	43	58	16	YKFLFTDLRIVYRDNN	0.83
HLA-DPA1*01:03/DPB1*04:01	39	51	13	RREVYKFLFTDLR	0.84
HLA-DRB4*01:01	20	34	15	EESVHEIRLQCVQCK	0.85
HLA-DRB4*01:01	21	35	15	ESVHEIRLQCVQCKK	0.85
HLA-DPA1*03:01/DPB1*04:02	40	54	15	REVYKFLFTDLRIVY	0.85
HLA-DPA1*03:01/DPB1*04:02	39	53	15	RREVYKFLFTDLRIV	0.87
HLA-DRB1*03:01	41	58	18	EVYKFLFTDLRIVYRDNN	0.89
HLA-DRB1*03:01	44	61	18	KFLFTDLRIVYRDNNPYG	0.89
HLA-DPA1*01:03/DPB1*04:01	37	54	18	LQRREVYKFLFTDLRIVY	0.89
HLA-DRB1*03:01	38	55	18	QRREVYKFLFTDLRIVYR	0.89
HLA-DRB1*03:01	40	57	18	REVYKFLFTDLRIVYRDN	0.89
HLA-DRB1*03:01	39	54	16	RREVYKFLFTDLRIVY	0.89
HLA-DRB1*03:01	39	56	18	RREVYKFLFTDLRIVYRD	0.89
HLA-DRB1*03:01	42	59	18	VYKFLFTDLRIVYRDNNP	0.89
HLA-DRB1*03:01	43	60	18	YKFLFTDLRIVYRDNNPY	0.89
HLA-DRB4*01:01	21	33	13	ESVHEIRLQCVQC	0.90
HLA-DPA1*03:01/DPB1*04:02	42	55	14	VYKFLFTDLRIVYR	0.90
HLA-DPA1*03:01/DPB1*04:02	41	54	14	EVYKFLFTDLRIVY	0.92
HLA-DRB1*03:01	40	54	15	REVYKFLFTDLRIVY	0.92
HLA-DRB4*01:01	20	33	14	EESVHEIRLQCVQC	0.93
HLA-DPA1*03:01/DPB1*04:02	43	56	14	YKFLFTDLRIVYRD	0.96
HLA-DPA1*01:03/DPB1*04:01	37	51	15	LQRREVYKFLFTDLR	0.97
HLA-DPA1*01:03/DPB1*04:01	39	56	18	RREVYKFLFTDLRIVYRD	0.98
HLA-DPA1*02:01/DPB1*05:01	42	55	14	VYKFLFTDLRIVYR	0.99
HLA-DPA1*01:03/DPB1*04:01	42	58	17	VYKFLFTDLRIVYRDNN	0.99
HLA-DPA1*03:01/DPB1*04:02	41	55	15	EVYKFLFTDLRIVYR	1.00
HLA-DPA1*01:03/DPB1*04:01	44	57	14	KFLFTDLRIVYRDN	1.02
HLA-DPA1*01:03/DPB1*04:01	37	50	14	LQRREVYKFLFTDL	1.03
HLA-DPA1*02:01/DPB1*01:01	40	53	14	REVYKFLFTDLRIV	1.04

HLA-DRB1*03:01	45	60	16	FLFTDLRIVYRDNNPY	1.06
HLA-DPA1*03:01/DPB1*04:02	42	54	13	VYKFLFTDLRIVY	1.06
HLA-DPA1*03:01/DPB1*04:02	43	55	13	YKFLFTDLRIVYR	1.08
HLA-DPA1*03:01/DPB1*04:02	41	53	13	EVYKFLFTDLRIV	1.09
HLA-DPA1*03:01/DPB1*04:02	42	56	15	VYKFLFTDLRIVYRD	1.10
HLA-DPA1*03:01/DPB1*04:02	43	57	15	YKFLFTDLRIVYRDN	1.10
HLA-DPA1*01:03/DPB1*04:01	38	50	13	QRREVYKFLFTDL	1.14
HLA-DPA1*01:03/DPB1*04:01	36	53	18	ELQRREVYKFLFTDLRIV	1.15
HLA-DPA1*01:03/DPB1*04:01	41	52	12	EVYKFLFTDLRI	1.16
HLA-DRB1*03:01	41	54	14	EVYKFLFTDLRIVY	1.18
HLA-DRB5*01:01	67	80	14	LRFLSKISEYRHYQ	1.18
HLA-DPA1*02:01/DPB1*01:01	39	52	14	RREVYKFLFTDLRI	1.18
HLA-DRB1*03:01	45	59	15	FLFTDLRIVYRDNNP	1.20
HLA-DRB4*01:01	19	33	15	LEESVHEIRLQCVQC	1.20
HLA-DRB5*01:01	67	79	13	LRFLSKISEYRHY	1.20
HLA-DPA1*02:01/DPB1*01:01	40	54	15	REVYKFLFTDLRIVY	1.20
HLA-DPA1*02:01/DPB1*05:01	40	54	15	REVYKFLFTDLRIVY	1.20
HLA-DPA1*02:01/DPB1*01:01	39	53	15	RREVYKFLFTDLRIV	1.20
HLA-DPA1*01:03/DPB1*04:01	40	57	18	REVYKFLFTDLRIVYRDN	1.24
HLA-DRB5*01:01	66	79	14	CLRFLSKISEYRHY	1.29
HLA-DPA1*02:01/DPB1*01:01	37	50	14	LQRREVYKFLFTDL	1.29
HLA-DPA1*02:01/DPB1*01:01	38	51	14	QRREVYKFLFTDLR	1.29
HLA-DPA1*02:01/DPB1*05:01	43	56	14	YKFLFTDLRIVYRD	1.29
HLA-DPA1*02:01/DPB1*05:01	41	55	15	EVYKFLFTDLRIVYR	1.30
HLA-DPA1*02:01/DPB1*05:01	42	56	15	VYKFLFTDLRIVYRD	1.30
HLA-DPA1*02:01/DPB1*01:01	39	51	13	RREVYKFLFTDLR	1.31
HLA-DPA1*02:01/DPB1*01:01	38	50	13	QRREVYKFLFTDL	1.33
HLA-DPA1*02:01/DPB1*01:01	40	52	13	REVYKFLFTDLRI	1.33
HLA-DRB1*03:01	42	54	13	VYKFLFTDLRIVY	1.34
HLA-DPA1*01:03/DPB1*04:01	36	50	15	ELQRREVYKFLFTDL	1.40
HLA-DPA1*02:01/DPB1*05:01	41	54	14	EVYKFLFTDLRIVY	1.40
HLA-DPA1*03:01/DPB1*04:02	44	57	14	KFLFTDLRIVYRDN	1.40
HLA-DPA1*02:01/DPB1*01:01	38	52	15	QRREVYKFLFTDLRI	1.40
HLA-DRB1*03:01	38	54	17	QRREVYKFLFTDLRIVY	1.40
HLA-DPA1*03:01/DPB1*04:02	42	53	12	VYKFLFTDLRIV	1.42
HLA-DPA1*02:01/DPB1*05:01	43	55	13	YKFLFTDLRIVYR	1.42
HLA-DPA1*03:01/DPB1*04:02	43	54	12	YKFLFTDLRIVY	1.45
HLA-DPA1*03:01/DPB1*04:02	41	56	16	EVYKFLFTDLRIVYRD	1.50
HLA-DRB1*03:01	45	61	17	FLFTDLRIVYRDNNPYG	1.50
HLA-DPA1*01:03/DPB1*04:01	44	58	15	KFLFTDLRIVYRDNN	1.50
HLA-DPA1*03:01/DPB1*04:02	40	55	16	REVYKFLFTDLRIVYR	1.50
HLA-DPA1*03:01/DPB1*04:02	39	54	16	RREVYKFLFTDLRIVY	1.50
HLA-DPA1*03:01/DPB1*04:02	42	57	16	VYKFLFTDLRIVYRDN	1.50

HLA-DPA1*01:03/DPB1*04:01	41	58	18	EVYKFLFTDLRIVYRDNN	1.51
HLA-DRB5*01:01	68	80	13	RFLSKISEYRHYQ	1.51
HLA-DPA1*01:03/DPB1*04:01	39	50	12	RREVYKFLFTDL	1.51
HLA-DRB5*01:01	66	78	13	CLRFLSKISEYRH	1.54
HLA-DPA1*01:03/DPB1*04:01	36	52	17	ELQRREVYKFLFTDLRI	1.55
HLA-DRB5*01:01	66	80	15	CLRFLSKISEYRHYQ	1.60
HLA-DPA1*03:01/DPB1*04:02	44	58	15	KFLFTDLRIVYRDNN	1.60
HLA-DPA1*02:01/DPB1*01:01	37	51	15	LQRREVYKFLFTDLR	1.60
HLA-DPA1*02:01/DPB1*05:01	38	52	15	QRREVYKFLFTDLRI	1.60
HLA-DRB4*01:01	20	35	16	EESVHEIRLQCVQCKK	1.62
HLA-DRB4*01:01	21	36	16	ESVHEIRLQCVQCKKE	1.62
HLA-DRB4*01:01	19	34	16	LEESVHEIRLQCVQCK	1.62
HLA-DPA1*03:01/DPB1*04:02	38	53	16	QRREVYKFLFTDLRIV	1.62
HLA-DPA1*02:01/DPB1*05:01	40	53	14	REVYKFLFTDLRIV	1.62
HLA-DPA1*02:01/DPB1*01:01	39	54	16	RREVYKFLFTDLRIVY	1.62
HLA-DRB4*01:01	22	37	16	SVHEIRLQCVQCKKEL	1.62
HLA-DRB4*01:01	23	38	16	VHEIRLQCVQCKKELQ	1.62
HLA-DPA1*01:03/DPB1*04:01	43	59	17	YKFLFTDLRIVYRDNNP	1.62
HLA-DRB1*03:01	43	54	12	YKFLFTDLRIVY	1.66
HLA-DRB5*01:01	67	81	15	LRFLSKISEYRHYQY	1.70
HLA-DPA1*02:01/DPB1*05:01	43	57	15	YKFLFTDLRIVYRDN	1.70
HLA-DPA1*01:03/DPB1*02:01	41	54	14	EVYKFLFTDLRIVY	1.72
HLA-DPA1*03:01/DPB1*04:02	44	56	13	KFLFTDLRIVYRD	1.72
HLA-DRB5*01:01	67	78	12	LRFLSKISEYRH	1.72
HLA-DRB5*01:01	65	78	14	MCLRFLSKISEYRH	1.72
HLA-DPA1*01:03/DPB1*02:01	40	53	14	REVYKFLFTDLRIV	1.72
HLA-DRB5*01:01	68	81	14	RFLSKISEYRHYQY	1.72
HLA-DPA1*01:03/DPB1*02:01	39	52	14	RREVYKFLFTDLRI	1.72
HLA-DPA1*02:01/DPB1*05:01	42	54	13	VYKFLFTDLRIVY	1.72
HLA-DPA1*02:01/DPB1*14:01	42	54	13	VYKFLFTDLRIVY	1.72
HLA-DPA1*01:03/DPB1*02:01	42	55	14	VYKFLFTDLRIVYR	1.72
HLA-DPA1*02:01/DPB1*14:01	43	55	13	YKFLFTDLRIVYR	1.72
HLA-DPA1*01:03/DPB1*04:01	36	51	16	ELQRREVYKFLFTDLR	1.73
HLA-DPA1*02:01/DPB1*01:01	40	55	16	REVYKFLFTDLRIVYR	1.73
HLA-DPA1*02:01/DPB1*05:01	40	55	16	REVYKFLFTDLRIVYR	1.73
HLA-DRB4*01:01	18	33	16	VLEESVHEIRLQCVQC	1.73
HLA-DRB5*01:01	68	79	12	RFLSKISEYRHY	1.78
HLA-DPA1*02:01/DPB1*01:01	36	50	15	ELQRREVYKFLFTDL	1.80
HLA-DPA1*02:01/DPB1*05:01	37	51	15	LQRREVYKFLFTDLR	1.80
HLA-DRB5*01:01	65	79	15	MCLRFLSKISEYRHY	1.80
HLA-DPA1*02:01/DPB1*05:01	39	53	15	RREVYKFLFTDLRIV	1.80
HLA-DRB1*03:01	45	58	14	FLFTDLRIVYRDNN	1.83
HLA-DQA1*04:01/DQB1*04:02	9	22	14	PRTLHELCEVLEES	1.83

HLA-DPA1*02:01/DPB1*14:01	42	55	14	VYKFLFTDLRIVYR	1.83
HLA-DPA1*02:01/DPB1*01:01	41	56	16	EVYKFLFTDLRIVYRD	1.85
HLA-DPA1*02:01/DPB1*05:01	41	56	16	EVYKFLFTDLRIVYRD	1.85
HLA-DPA1*02:01/DPB1*01:01	38	53	16	QRREVYKFLFTDLRIV	1.85
HLA-DPA1*02:01/DPB1*05:01	39	54	16	RREVYKFLFTDLRIVY	1.85
HLA-DPA1*02:01/DPB1*05:01	42	57	16	VYKFLFTDLRIVYRDN	1.85
HLA-DQA1*04:01/DQB1*04:02	10	22	13	RTLHELCEVLEES	1.87
HLA-DPA1*01:03/DPB1*02:01	41	55	15	EVYKFLFTDLRIVYR	1.90
HLA-DPA1*01:03/DPB1*02:01	38	52	15	QRREVYKFLFTDLRI	1.90
HLA-DPA1*01:03/DPB1*02:01	40	54	15	REVYKFLFTDLRIVY	1.90
HLA-DPA1*01:03/DPB1*02:01	39	53	15	RREVYKFLFTDLRIV	1.90
HLA-DPA1*01:03/DPB1*02:01	42	56	15	VYKFLFTDLRIVYRD	1.90
HLA-DRB3*01:01	51	64	14	RIVYRDNNPYGVCI	1.94
HLA-DQA1*04:01/DQB1*04:02	10	23	14	RTLHELCEVLEESV	1.94
HLA-DPA1*03:01/DPB1*04:02	44	59	16	KFLFTDLRIVYRDNNP	1.96
HLA-DPA1*02:01/DPB1*01:01	39	50	12	RREVYKFLFTDL	1.96
HLA-DPA1*02:01/DPB1*01:01	42	57	16	VYKFLFTDLRIVYRDN	1.96
HLA-DPA1*03:01/DPB1*04:02	43	58	16	YKFLFTDLRIVYRDNN	1.96
HLA-DRB1*11:01	61	75	15	GVCIMCLRFLSKISE	2.00
HLA-DRB3*01:01	50	64	15	LRIVYRDNNPYGVCI	2.00
HLA-DPA1*02:01/DPB1*14:01	43	54	12	YKFLFTDLRIVY	2.02
HLA-DRB1*11:01	63	76	14	CIMCLRFLSKISEY	2.05
HLA-DPA1*02:01/DPB1*01:01	41	54	14	EVYKFLFTDLRIVY	2.05
HLA-DRB1*11:01	64	77	14	IMCLRFLSKISEYR	2.05
HLA-DRB3*01:01	50	63	14	LRIVYRDNNPYGVC	2.05
HLA-DPA1*02:01/DPB1*05:01	38	51	14	QRREVYKFLFTDLR	2.05
HLA-DRB1*11:01	62	75	14	VCIMCLRFLSKISE	2.05
HLA-DPA1*01:03/DPB1*02:01	43	56	14	YKFLFTDLRIVYRD	2.05
HLA-DPA1*03:01/DPB1*04:02	45	60	16	FLFTDLRIVYRDNNPY	2.08
HLA-DRB1*11:01	63	77	15	CIMCLRFLSKISEYR	2.10
HLA-DRB5*01:01	64	78	15	IMCLRFLSKISEYRH	2.10
HLA-DRB3*01:01	51	65	15	RIVYRDNNPYGVCIM	2.10
HLA-DRB1*11:01	65	78	14	MCLRFLSKISEYRH	2.15
HLA-DPA1*02:01/DPB1*01:01	42	55	14	VYKFLFTDLRIVYR	2.15
HLA-DPA1*01:03/DPB1*02:01	41	53	13	EVYKFLFTDLRIV	2.18
HLA-DPA1*03:01/DPB1*04:02	45	57	13	FLFTDLRIVYRDN	2.18
HLA-DPA1*01:03/DPB1*02:01	40	52	13	REVYKFLFTDLRI	2.18
HLA-DQA1*04:01/DQB1*04:02	11	23	13	TLHELCEVLEESV	2.18
HLA-DPA1*01:03/DPB1*02:01	42	54	13	VYKFLFTDLRIVY	2.18
HLA-DPA1*01:03/DPB1*02:01	43	55	13	YKFLFTDLRIVYR	2.18
HLA-DPA1*02:01/DPB1*01:01	37	52	16	LQRREVYKFLFTDLRI	2.19
HLA-DPA1*02:01/DPB1*05:01	37	52	16	LQRREVYKFLFTDLRI	2.19
HLA-DPA1*02:01/DPB1*05:01	38	53	16	QRREVYKFLFTDLRIV	2.19

HLA-DRB1*11:01	64	78	15	IMCLRFLSKISEYRH	2.20
HLA-DQA1*04:01/DQB1*04:02	9	23	15	PRTLHELCEVLEESV	2.20
HLA-DQA1*04:01/DQB1*04:02	8	22	15	RPRTLHELCEVLEES	2.20
HLA-DRB1*11:01	62	76	15	VCIMCLRFLSKISEY	2.20
HLA-DPA1*03:01/DPB1*04:02	45	58	14	FLFTDLRIVYRDNN	2.26
HLA-DPA1*01:03/DPB1*02:01	37	50	14	LQRREVYKFLFTDL	2.26
HLA-DPA1*01:03/DPB1*02:01	38	51	14	QRREVYKFLFTDLR	2.26
HLA-DRB5*01:01	40	53	14	REVYKFLFTDLRIV	2.26
HLA-DQA1*04:01/DQB1*04:02	11	22	12	TLHELCEVLEES	2.28
HLA-DRB1*11:01	65	79	15	MCLRFLSKISEYRHY	2.30
HLA-DPA1*02:01/DPB1*05:01	36	51	16	ELQRREVYKFLFTDLR	2.31
HLA-DPA1*01:03/DPB1*02:01	41	56	16	EVYKFLFTDLRIVYRD	2.31
HLA-DPA1*01:03/DPB1*04:01	35	50	16	KELQRREVYKFLFTDL	2.31
HLA-DPA1*01:03/DPB1*02:01	37	52	16	LQRREVYKFLFTDLRI	2.31
HLA-DPA1*01:03/DPB1*02:01	38	53	16	QRREVYKFLFTDLRIV	2.31
HLA-DPA1*01:03/DPB1*02:01	40	55	16	REVYKFLFTDLRIVYR	2.31
HLA-DPA1*01:03/DPB1*02:01	39	54	16	RREVYKFLFTDLRIVY	2.31
HLA-DPA1*01:03/DPB1*02:01	42	57	16	VYKFLFTDLRIVYRDN	2.31
HLA-DPA1*01:03/DPB1*04:01	42	59	18	VYKFLFTDLRIVYRDNNP	2.31
HLA-DRB1*11:01	63	75	13	CIMCLRFLSKISE	2.34
HLA-DRB1*11:01	64	76	13	IMCLRFLSKISEY	2.34
HLA-DRB3*01:01	50	62	13	LRIVYRDNNPYGV	2.34
HLA-DRB1*11:01	65	77	13	MCLRFLSKISEYR	2.34
HLA-DRB3*01:01	51	63	13	RIVYRDNNPYGVC	2.34
HLA-DPA1*02:01/DPB1*01:01	42	54	13	VYKFLFTDLRIVY	2.34
HLA-DPA1*02:01/DPB1*05:01	37	50	14	LQRREVYKFLFTDL	2.37
HLA-DPA1*03:01/DPB1*04:02	41	57	17	EVYKFLFTDLRIVYRDN	2.38
HLA-DPA1*03:01/DPB1*04:02	38	54	17	QRREVYKFLFTDLRIVY	2.38
HLA-DPA1*03:01/DPB1*04:02	40	56	17	REVYKFLFTDLRIVYRD	2.38
HLA-DPA1*03:01/DPB1*04:02	39	55	17	RREVYKFLFTDLRIVYR	2.38
HLA-DPA1*03:01/DPB1*04:02	42	58	17	VYKFLFTDLRIVYRDNN	2.38
HLA-DRB3*01:01	49	63	15	DLRIVYRDNNPYGVC	2.40
HLA-DPA1*02:01/DPB1*05:01	36	50	15	ELQRREVYKFLFTDL	2.40
HLA-DPA1*02:01/DPB1*01:01	41	55	15	EVYKFLFTDLRIVYR	2.40
HLA-DRB1*03:01	45	62	18	FLFTDLRIVYRDNNPYGV	2.40
HLA-DPA1*01:03/DPB1*04:01	40	51	12	REVYKFLFTDLR	2.40
HLA-DPA1*02:01/DPB1*01:01	36	51	16	ELQRREVYKFLFTDLR	2.42
HLA-DRB3*01:01	49	62	14	DLRIVYRDNNPYGV	2.48
HLA-DRB5*01:01	41	54	14	EVYKFLFTDLRIVY	2.48
HLA-DRB1*03:01	46	59	14	LFTDLRIVYRDNNP	2.48
HLA-DPA1*02:01/DPB1*05:01	39	52	14	RREVYKFLFTDLRI	2.48
HLA-DRB5*01:01	39	52	14	RREVYKFLFTDLRI	2.48
HLA-DPA1*02:01/DPB1*01:01	79	92	14	YQYSLYGKTLEERV	2.48

HLA-DRB1*03:01	45	57	13	FLFTDLRIVYRDN	2.49
HLA-DPA1*01:03/DPB1*02:01	36	50	15	ELQRREVYKFLFTDL	2.50
HLA-DPA1*01:03/DPB1*02:01	37	51	15	LQRREVYKFLFTDLR	2.50
HLA-DRB3*02:02	50	64	15	LRIVYRDNNPYGVCI	2.50
HLA-DRB3*02:02	51	65	15	RIVYRDNNPYGVCIM	2.50
HLA-DPA1*02:01/DPB1*01:01	35	50	16	KELQRREVYKFLFTDL	2.54
HLA-DRB3*02:02	50	65	16	LRIVYRDNNPYGVCIM	2.54
HLA-DPA1*03:01/DPB1*04:02	37	53	17	LQRREVYKFLFTDLRIV	2.55
HLA-DPA1*02:01/DPB1*01:01	39	55	17	RREVYKFLFTDLRIVYR	2.55
HLA-DPA1*02:01/DPB1*05:01	39	55	17	RREVYKFLFTDLRIVYR	2.55
HLA-DRB4*01:01	18	34	17	VLEESVHEIRLQCVQCK	2.55
HLA-DRB1*03:01	37	54	18	LQRREVYKFLFTDLRIVY	2.57
HLA-DQA1*01:02/DQB1*06:02	20	33	14	EESVHEIRLQCVQC	2.58
HLA-DPA1*02:01/DPB1*14:01	41	54	14	EVYKFLFTDLRIVY	2.58
HLA-DPA1*02:01/DPB1*05:01	35	48	14	KELQRREVYKFLFT	2.58
HLA-DPA1*02:01/DPB1*14:01	42	53	12	VYKFLFTDLRIV	2.58
HLA-DRB5*01:01	41	55	15	EVYKFLFTDLRIVYR	2.60
HLA-DRB5*01:01	40	54	15	REVYKFLFTDLRIVY	2.60
HLA-DPA1*02:01/DPB1*01:01	42	56	15	VYKFLFTDLRIVYRD	2.60
HLA-DPA1*01:03/DPB1*02:01	43	57	15	YKFLFTDLRIVYRDN	2.60
HLA-DPA1*02:01/DPB1*05:01	36	48	13	ELQRREVYKFLFT	2.65
HLA-DPA1*02:01/DPB1*05:01	44	56	13	KFLFTDLRIVYRD	2.65
HLA-DPA1*01:03/DPB1*04:01	44	59	16	KFLFTDLRIVYRDNNP	2.65
HLA-DRB5*01:01	65	80	16	MCLRFLSKISEYRHYQ	2.65
HLA-DPA1*02:01/DPB1*05:01	43	54	12	YKFLFTDLRIVY	2.67
HLA-DQA1*01:02/DQB1*06:02	21	34	14	ESVHEIRLQCVQCK	2.69
HLA-DQA1*01:02/DQB1*06:02	19	32	14	LEESVHEIRLQCVQ	2.69
HLA-DRB1*03:01	46	60	15	LFTDLRIVYRDNNPY	2.70
HLA-DRB5*01:01	68	82	15	RFLSKISEYRHYQYS	2.70
HLA-DRB5*01:01	39	53	15	RREVYKFLFTDLRIV	2.70
HLA-DRB4*01:01	20	36	17	EESVHEIRLQCVQCKKE	2.73
HLA-DRB4*01:01	21	37	17	ESVHEIRLQCVQCKKEL	2.73
HLA-DPA1*03:01/DPB1*04:02	45	56	12	FLFTDLRIVYRD	2.73
HLA-DPA1*03:01/DPB1*04:02	44	55	12	KFLFTDLRIVYR	2.73
HLA-DRB4*01:01	19	35	17	LEESVHEIRLQCVQCKK	2.73
HLA-DPA1*02:01/DPB1*01:01	38	54	17	QRREVYKFLFTDLRIVY	2.73
HLA-DRB4*01:01	22	38	17	SVHEIRLQCVQCKKELQ	2.73
HLA-DRB4*01:01	23	39	17	VHEIRLQCVQCKKELQR	2.73
HLA-DRB5*01:01	64	79	16	IMCLRFLSKISEYRHY	2.77
HLA-DPA1*02:01/DPB1*05:01	43	58	16	YKFLFTDLRIVYRDNN	2.77
HLA-DPA1*02:01/DPB1*05:01	36	49	14	ELQRREVYKFLFTD	2.80
HLA-DPA1*01:03/DPB1*04:01	75	88	14	EYRHYQYSLYGKTL	2.80
HLA-DQA1*01:01/DQB1*05:01	40	53	14	REVYKFLFTDLRIV	2.80

HLA-DQA1*01:01/DQB1*05:01	40	54	15	REVYKFLFTDLRIVY	2.80
HLA-DRB3*01:01	48	62	15	TDLRIVYRDNNPYGV	2.80
HLA-DQA1*04:01/DQB1*04:02	11	24	14	TLHELCEVLEESVH	2.80
HLA-DQA1*04:01/DQB1*04:02	11	25	15	TLHELCEVLEESVHE	2.80
HLA-DQA1*01:02/DQB1*06:02	20	32	13	EESVHEIRLQCVQ	2.81
HLA-DQA1*01:02/DQB1*06:02	21	33	13	ESVHEIRLQCVQC	2.81
HLA-DPA1*02:01/DPB1*01:01	41	53	13	EVYKFLFTDLRIV	2.81
HLA-DRB5*01:01	41	53	13	EVYKFLFTDLRIV	2.81
HLA-DPA1*01:03/DPB1*02:01	38	50	13	QRREVYKFLFTDL	2.81
HLA-DRB5*01:01	40	52	13	REVYKFLFTDLRI	2.81
HLA-DPA1*01:03/DPB1*02:01	39	51	13	RREVYKFLFTDLR	2.81
HLA-DRB1*03:01	45	56	12	FLFTDLRIVYRD	2.82
HLA-DRB1*11:01	63	78	16	CIMCLRFLSKISEYRH	2.89
HLA-DRB5*01:01	66	81	16	CLRFLSKISEYRHYQY	2.89
HLA-DRB1*11:01	61	76	16	GVCIMCLRFLSKISEY	2.89
HLA-DRB1*11:01	64	79	16	IMCLRFLSKISEYRHY	2.89
HLA-DRB1*11:01	65	80	16	MCLRFLSKISEYRHYQ	2.89
HLA-DRB1*11:01	62	77	16	VCIMCLRFLSKISEYR	2.89
HLA-DRB1*11:01	60	75	16	YGVCIMCLRFLSKISE	2.89
HLA-DRB4*01:01	17	33	17	EVLEESVHEIRLQCVQC	2.90
HLA-DPA1*02:01/DPB1*05:01	38	54	17	QRREVYKFLFTDLRIVY	2.90
HLA-DPA1*02:01/DPB1*01:01	40	56	17	REVYKFLFTDLRIVYRD	2.90
HLA-DPA1*02:01/DPB1*05:01	40	56	17	REVYKFLFTDLRIVYRD	2.90
HLA-DPA1*01:03/DPB1*04:01	74	88	15	SEYRHYQYSLYGKTL	2.90
HLA-DPA1*02:01/DPB1*01:01	79	93	15	YQYSLYGKTLEERVK	2.90
HLA-DRB3*01:01	52	65	14	IVYRDNNPYGVCIM	2.91
HLA-DQA1*01:01/DQB1*05:01	41	53	13	EVYKFLFTDLRIV	2.96
HLA-DQA1*01:02/DQB1*06:02	19	31	13	LEESVHEIRLQCV	2.96
HLA-DRB1*03:01	46	58	13	LFTDLRIVYRDNN	2.96
HLA-DPA1*02:01/DPB1*05:01	38	50	13	QRREVYKFLFTDL	2.96
HLA-DQA1*01:01/DQB1*05:01	42	54	13	VYKFLFTDLRIVY	2.96
HLA-DQA1*01:02/DQB1*06:02	20	34	15	EESVHEIRLQCVQCK	3.00
HLA-DPA1*01:03/DPB1*04:01	75	89	15	EYRHYQYSLYGKTLE	3.00
HLA-DPA1*02:01/DPB1*01:01	78	92	15	HYQYSLYGKTLEERV	3.00
HLA-DQA1*01:02/DQB1*06:02	19	33	15	LEESVHEIRLQCVQC	3.00
HLA-DRB5*01:01	38	52	15	QRREVYKFLFTDLRI	3.00
HLA-DQA1*01:02/DQB1*06:02	18	32	15	VLEESVHEIRLQCVQ	3.00
HLA-DPA1*02:01/DPB1*14:01	43	56	14	YKFLFTDLRIVYRD	3.02
HLA-DPA1*02:01/DPB1*01:01	41	57	17	EVYKFLFTDLRIVYRDN	3.07
HLA-DPA1*02:01/DPB1*05:01	41	57	17	EVYKFLFTDLRIVYRDN	3.07
HLA-DPA1*03:01/DPB1*04:02	44	60	17	KFLFTDLRIVYRDNNPY	3.07
HLA-DPA1*02:01/DPB1*01:01	37	53	17	LQRREVYKFLFTDLRIV	3.07
HLA-DPA1*03:01/DPB1*04:02	43	59	17	YKFLFTDLRIVYRDNNP	3.07

HLA-DPA1*03:01/DPB1*04:02	45	59	15	FLFTDLRIVYRDNNP	3.10
HLA-DQA1*04:01/DQB1*04:02	10	24	15	RTLHELCEVLEESVH	3.10
HLA-DRB3*02:02	49	64	16	DLRIVYRDNNPYGVCI	3.12
HLA-DPA1*01:03/DPB1*02:01	36	51	16	ELQRREVYKFLFTDLR	3.12
HLA-DPA1*02:01/DPB1*05:01	41	53	13	EVYKFLFTDLRIV	3.12
HLA-DPA1*02:01/DPB1*14:01	41	53	13	EVYKFLFTDLRIV	3.12
HLA-DQA1*01:01/DQB1*05:01	41	54	14	EVYKFLFTDLRIVY	3.12
HLA-DPA1*02:01/DPB1*01:01	78	91	14	HYQYSLYGKTLEER	3.12
HLA-DPA1*01:03/DPB1*02:01	35	50	16	KELQRREVYKFLFTDL	3.12
HLA-DPA1*02:01/DPB1*05:01	44	57	14	KFLFTDLRIVYRDN	3.12
HLA-DRB5*01:01	67	82	16	LRFLSKISEYRHYQYS	3.12
HLA-DPA1*02:01/DPB1*05:01	40	52	13	REVYKFLFTDLRI	3.12
HLA-DRB3*02:02	51	66	16	RIVYRDNNPYGVCIMC	3.12
HLA-DRB1*12:01	63	77	15	CIMCLRFLSKISEYR	3.15
HLA-DQA1*01:02/DQB1*06:02	21	35	15	ESVHEIRLQCVQCKK	3.20
HLA-DPA1*02:01/DPB1*05:01	35	49	15	KELQRREVYKFLFTD	3.20
HLA-DRB3*01:01	49	64	16	DLRIVYRDNNPYGVCI	3.23
HLA-DRB1*12:01	64	77	14	IMCLRFLSKISEYR	3.23
HLA-DRB3*01:01	50	65	16	LRIVYRDNNPYGVCIM	3.23
HLA-DRB3*02:02	51	64	14	RIVYRDNNPYGVCI	3.23
HLA-DRB3*01:01	51	66	16	RIVYRDNNPYGVCIMC	3.23
HLA-DQA1*01:01/DQB1*05:01	42	55	14	VYKFLFTDLRIVYR	3.23
HLA-DPA1*01:03/DPB1*04:01	76	89	14	YRHYQYSLYGKTLE	3.23
HLA-DPA1*02:01/DPB1*01:01	42	58	17	VYKFLFTDLRIVYRDNN	3.24
HLA-DPA1*01:03/DPB1*02:01	41	52	12	EVYKFLFTDLRI	3.26
HLA-DPA1*02:01/DPB1*05:01	44	55	12	KFLFTDLRIVYR	3.26
HLA-DPA1*02:01/DPB1*14:01	44	55	12	KFLFTDLRIVYR	3.26
HLA-DRB1*11:01	65	76	12	MCLRFLSKISEY	3.26
HLA-DPA1*02:01/DPB1*01:01	40	51	12	REVYKFLFTDLR	3.26
HLA-DPA1*01:03/DPB1*02:01	42	53	12	VYKFLFTDLRIV	3.26
HLA-DQA1*01:01/DQB1*05:01	42	53	12	VYKFLFTDLRIV	3.26
HLA-DPA1*01:03/DPB1*02:01	43	54	12	YKFLFTDLRIVY	3.26
HLA-DRB3*01:01	52	64	13	IVYRDNNPYGVCI	3.27
HLA-DPA1*02:01/DPB1*01:01	79	91	13	YQYSLYGKTLEER	3.27
HLA-DPA1*02:01/DPB1*05:01	34	48	15	KKELQRREVYKFLFT	3.3
HLA-DRB1*13:02	49	62	14	DLRIVYRDNNPYGV	3.34
HLA-DRB1*12:01	64	78	15	IMCLRFLSKISEYRH	3.35
HLA-DRB1*12:01	65	78	14	MCLRFLSKISEYRH	3.39
HLA-DPA1*02:01/DPB1*14:01	41	55	15	EVYKFLFTDLRIVYR	3.40
HLA-DPA1*02:01/DPB1*01:01	77	91	15	RHYQYSLYGKTLEER	3.40
HLA-DPA1*03:01/DPB1*04:02	45	61	17	FLFTDLRIVYRDNNPYG	3.41
HLA-DPA1*01:03/DPB1*04:01	35	51	17	KELQRREVYKFLFTDLR	3.41
HLA-DPA1*02:01/DPB1*05:01	42	58	17	VYKFLFTDLRIVYRDNN	3.41

HLA-DRB5*01:01	64	77	14	IMCLRFLSKISEYR	3.45
HLA-DRB1*13:02	50	63	14	LRIVYRDNNPYGVC	3.45
HLA-DPA1*02:01/DPB1*14:01	40	53	14	REVYKFLFTDLRIV	3.45
HLA-DRB1*04:01	42	55	14	VYKFLFTDLRIVYR	3.45
HLA-DRB1*04:01	43	56	14	YKFLFTDLRIVYRD	3.45
HLA-DRB5*01:01	63	78	16	CIMCLRFLSKISEYRH	3.46
HLA-DPA1*01:03/DPB1*04:01	73	88	16	ISEYRHYQYSLYGKTL	3.46
HLA-DRB1*03:01	46	61	16	LFTDLRIVYRDNNPYG	3.46
HLA-DRB3*01:01	48	63	16	TDLRIVYRDNNPYGVC	3.46
HLA-DPA1*01:03/DPB1*04:01	76	90	15	YRHYQYSLYGKTLEE	3.50
HLA-DRB1*11:01	66	79	14	CLRFLSKISEYRHY	3.55
HLA-DPA1*01:03/DPB1*04:01	35	52	18	KELQRREVYKFLFTDLRI	3.55
HLA-DRB4*01:01	19	32	14	LEESVHEIRLQCVQ	3.55
HLA-DRB4*01:01	22	35	14	SVHEIRLQCVQCKK	3.55
HLA-DRB4*01:01	23	36	14	VHEIRLQCVQCKKE	3.55
HLA-DQA1*01:02/DQB1*06:02	20	31	12	EESVHEIRLQCV	3.56
HLA-DRB3*01:01	51	62	12	RIVYRDNNPYGV	3.56
HLA-DPA1*02:01/DPB1*01:01	36	52	17	ELQRREVYKFLFTDLRI	3.58
HLA-DPA1*02:01/DPB1*05:01	36	52	17	ELQRREVYKFLFTDLRI	3.58
HLA-DPA1*02:01/DPB1*05:01	37	53	17	LQRREVYKFLFTDLRIV	3.58
HLA-DPA1*02:01/DPB1*05:01	37	49	13	LQRREVYKFLFTD	3.59
HLA-DRB1*13:02	50	62	13	LRIVYRDNNPYGV	3.59
HLA-DPA1*02:01/DPB1*01:01	80	92	13	QYSLYGKTLEERV	3.59
HLA-DPA1*02:01/DPB1*05:01	39	51	13	RREVYKFLFTDLR	3.59
HLA-DRB4*01:01	22	34	13	SVHEIRLQCVQCK	3.59
HLA-DRB4*01:01	23	35	13	VHEIRLQCVQCKK	3.59
HLA-DRB1*13:02	50	64	15	LRIVYRDNNPYGVCI	3.60
HLA-DRB1*12:01	65	79	15	MCLRFLSKISEYRHY	3.60
HLA-DQA1*01:01/DQB1*05:01	39	53	15	RREVYKFLFTDLRIV	3.60
HLA-DPA1*02:01/DPB1*05:01	35	50	16	KELQRREVYKFLFTDL	3.69
HLA-DPA1*01:03/DPB1*02:01	43	58	16	YKFLFTDLRIVYRDNN	3.69
HLA-DRB1*04:01	41	55	15	EVYKFLFTDLRIVYR	3.70
HLA-DRB1*15:01	64	78	15	IMCLRFLSKISEYRH	3.70
HLA-DRB1*04:01	40	54	15	REVYKFLFTDLRIVY	3.70
HLA-DRB1*04:01	42	56	15	VYKFLFTDLRIVYRD	3.70
HLA-DRB1*04:01	43	57	15	YKFLFTDLRIVYRDN	3.70
HLA-DRB1*11:01	66	78	13	CLRFLSKISEYRH	3.74
HLA-DRB5*01:01	65	77	13	MCLRFLSKISEYR	3.74
HLA-DPA1*01:03/DPB1*04:01	76	88	13	YRHYQYSLYGKTL	3.74
HLA-DPA1*01:03/DPB1*02:01	36	52	17	ELQRREVYKFLFTDLRI	3.75
HLA-DPA1*01:03/DPB1*02:01	41	57	17	EVYKFLFTDLRIVYRDN	3.75
HLA-DPA1*01:03/DPB1*02:01	37	53	17	LQRREVYKFLFTDLRIV	3.75
HLA-DPA1*01:03/DPB1*02:01	38	54	17	QRREVYKFLFTDLRIVY	3.75

HLA-DPA1*01:03/DPB1*02:01	40	56	17	REVYKFLFTDLRIVYRD	3.75
HLA-DPA1*01:03/DPB1*02:01	39	55	17	RREVYKFLFTDLRIVYR	3.75
HLA-DPA1*01:03/DPB1*02:01	42	58	17	VYKFLFTDLRIVYRDNN	3.75
HLA-DRB1*04:01	41	54	14	EVYKFLFTDLRIVY	3.77
HLA-DPA1*02:01/DPB1*05:01	34	47	14	KKELQRREVYKFLF	3.77
HLA-DRB3*02:02	50	63	14	LRIVYRDNNPYGVC	3.77
HLA-DPA1*02:01/DPB1*01:01	80	93	14	QYSLYGKTLEERVK	3.77
HLA-DRB1*13:02	51	64	14	RIVYRDNNPYGVCI	3.77
HLA-DQA1*01:02/DQB1*06:02	18	31	14	VLEESVHEIRLQCV	3.77
HLA-DQA1*01:01/DQB1*05:01	41	55	15	EVYKFLFTDLRIVYR	3.80
HLA-DRB3*01:01	52	66	15	IVYRDNNPYGVCIMC	3.80
HLA-DQA1*01:01/DQB1*05:01	42	56	15	VYKFLFTDLRIVYRD	3.80
HLA-DRB1*11:01	66	81	16	CLRFLSKISEYRHYQY	3.81
HLA-DQA1*04:01/DQB1*04:02	8	23	16	RPRTLHELCEVLEESV	3.81
HLA-DPA1*01:03/DPB1*04:01	74	89	16	SEYRHYQYSLYGKTLE	3.81
HLA-DQA1*01:02/DQB1*06:02	21	32	12	ESVHEIRLQCVQ	3.85
HLA-DRB5*01:01	41	52	12	EVYKFLFTDLRI	3.85
HLA-DPA1*01:03/DPB1*04:01	43	60	18	YKFLFTDLRIVYRDNNPY	3.85
HLA-DRB4*01:01	20	32	13	EESVHEIRLQCVQ	3.90
HLA-DRB4*01:01	23	37	15	VHEIRLQCVQCKKEL	3.90
HLA-DPA1*02:01/DPB1*01:01	35	51	17	KELQRREVYKFLFTDLR	3.92
HLA-DRB1*11:01	67	82	16	LRFLSKISEYRHYQYS	3.92
HLA-DRB3*02:02	50	66	17	LRIVYRDNNPYGVCIMC	3.92
HLA-DQA1*04:01/DQB1*04:02	10	25	16	RTLHELCEVLEESVHE	3.92
HLA-DQA1*04:01/DQB1*04:02	7	22	16	TRPRTLHELCEVLEES	3.92
HLA-DPA1*01:03/DPB1*04:01	60	73	14	YGVCIMCLRFLSKI	3.98
HLA-DPA1*02:01/DPB1*14:01	40	54	15	REVYKFLFTDLRIVY	4.00
HLA-DRB1*13:02	48	62	15	TDLRIVYRDNNPYGV	4.00
HLA-DPA1*02:01/DPB1*14:01	42	56	15	VYKFLFTDLRIVYRD	4.00
HLA-DRB3*01:01	47	62	16	FTDLRIVYRDNNPYGV	4.04
HLA-DPA1*02:01/DPB1*05:01	35	47	13	KELQRREVYKFLF	4.05
HLA-DRB3*02:02	49	65	17	DLRIVYRDNNPYGVCIM	4.09
HLA-DPA1*02:01/DPB1*05:01	35	51	17	KELQRREVYKFLFTDLR	4.09
HLA-DRB5*01:01	63	77	15	CIMCLRFLSKISEYR	4.10
HLA-DRB3*02:02	49	63	15	DLRIVYRDNNPYGVC	4.10
HLA-DRB4*01:01	22	36	15	SVHEIRLQCVQCKKE	4.10
HLA-DRB4*01:01	18	32	15	VLEESVHEIRLQCVQ	4.10
HLA-DRB1*11:01	64	75	12	IMCLRFLSKISE	4.15
HLA-DPA1*02:01/DPB1*01:01	42	53	12	VYKFLFTDLRIV	4.15
HLA-DQA1*04:01/DQB1*04:02	9	24	16	PRTLHELCEVLEESVH	4.16
HLA-DRB1*13:02	49	63	15	DLRIVYRDNNPYGVC	4.20
HLA-DPA1*02:01/DPB1*05:01	44	58	15	KFLFTDLRIVYRDNN	4.20
HLA-DRB1*12:01	67	81	15	LRFLSKISEYRHYQY	4.20

HLA-DRB1*12:01	63	78	16	CIMCLRFLSKISEYRH	4.27
HLA-DPA1*01:03/DPB1*04:01	75	90	16	EYRHYQYSLYGKTLEE	4.27
HLA-DRB1*12:01	64	79	16	IMCLRFLSKISEYRHY	4.27
HLA-DRB1*11:01	66	80	15	CLRFLSKISEYRHYQ	4.30
HLA-DRB1*12:01	65	77	13	MCLRFLSKISEYR	4.37
HLA-DRB1*13:02	51	63	13	RIVYRDNNPYGVC	4.37
HLA-DRB1*04:01	42	54	13	VYKFLFTDLRIVY	4.37
HLA-DRB1*04:01	43	55	13	YKFLFTDLRIVYR	4.37
HLA-DRB1*12:01	62	77	16	VCIMCLRFLSKISEYR	4.39
HLA-DRB1*13:02	51	65	15	RIVYRDNNPYGVCIM	4.40
HLA-DPA1*01:03/DPB1*02:01	44	57	14	KFLFTDLRIVYRDN	4.41
HLA-DPA1*03:01/DPB1*04:02	38	55	18	QRREVYKFLFTDLRIVYR	4.44
HLA-DPA1*03:01/DPB1*04:02	39	56	18	RREVYKFLFTDLRIVYRD	4.44
HLA-DPA1*02:01/DPB1*05:01	37	48	12	LQRREVYKFLFT	4.45
HLA-DPA1*01:03/DPB1*02:01	39	50	12	RREVYKFLFTDL	4.45
HLA-DRB4*01:01	23	34	12	VHEIRLQCVQCK	4.45
HLA-DRB1*15:01	64	79	16	IMCLRFLSKISEYRHY	4.50
HLA-DRB5*01:01	40	55	16	REVYKFLFTDLRIVYR	4.50
HLA-DQA1*04:01/DQB1*04:02	11	26	16	TLHELCEVLEESVHEI	4.50
HLA-DRB1*12:01	66	79	14	CLRFLSKISEYRHY	4.52
HLA-DPA1*01:03/DPB1*04:01	61	74	14	GVCIMCLRFLSKIS	4.52
HLA-DPA1*01:03/DPB1*02:01	44	56	13	KFLFTDLRIVYRD	4.52
HLA-DRB5*01:01	64	80	17	IMCLRFLSKISEYRHYQ	4.60
HLA-DPA1*01:03/DPB1*04:01	34	50	17	KKELQRREVYKFLFTDL	4.60
HLA-DPA1*02:01/DPB1*01:01	34	50	17	KKELQRREVYKFLFTDL	4.60
HLA-DRB5*01:01	68	83	16	RFLSKISEYRHYQYSL	4.62
HLA-DPA1*02:01/DPB1*01:01	76	91	16	YRHYQYSLYGKTLEER	4.62
HLA-DRB5*01:01	114	127	14	EKERHVNANKRFHN	4.63
HLA-DRB1*04:01	23	36	14	VHEIRLQCVQCKKE	4.63
HLA-DPA1*02:01/DPB1*01:01	43	56	14	YKFLFTDLRIVYRD	4.63
HLA-DPA1*01:03/DPB1*04:01	61	73	13	GVCIMCLRFLSKI	4.68
HLA-DRB5*01:01	41	56	16	EVYKFLFTDLRIVYRD	4.73
HLA-DRB5*01:01	39	54	16	RREVYKFLFTDLRIVY	4.73
HLA-DRB5*01:01	42	57	16	VYKFLFTDLRIVYRDN	4.73
HLA-DPA1*01:03/DPB1*04:01	76	91	16	YRHYQYSLYGKTLEER	4.73
HLA-DRB1*11:01	66	77	12	CLRFLSKISEYR	4.74
HLA-DRB5*01:01	66	77	12	CLRFLSKISEYR	4.74
HLA-DPA1*03:01/DPB1*04:02	41	58	18	EVYKFLFTDLRIVYRDNN	4.74
HLA-DRB3*01:01	52	63	12	IVYRDNNPYGVC	4.74
HLA-DPA1*01:03/DPB1*02:01	44	55	12	KFLFTDLRIVYR	4.74
HLA-DRB1*03:01	46	57	12	LFTDLRIVYRDN	4.74
HLA-DPA1*03:01/DPB1*04:02	37	54	18	LQRREVYKFLFTDLRIVY	4.74
HLA-DPA1*02:01/DPB1*05:01	38	55	18	QRREVYKFLFTDLRIVYR	4.74

HLA-DPA1*03:01/DPB1*04:02	40	57	18	REVYKFLFTDLRIVYRDN	4.74
HLA-DQA1*04:01/DQB1*04:02	8	21	14	RPRTLHELCEVLEE	4.74
HLA-DPA1*03:01/DPB1*04:02	39	52	14	RREVYKFLFTDLRI	4.74
HLA-DPA1*02:01/DPB1*05:01	42	53	12	VYKFLFTDLRIV	4.74
HLA-DPA1*03:01/DPB1*04:02	42	59	18	VYKFLFTDLRIVYRDNNP	4.74
HLA-DRB1*11:01	63	79	17	CIMCLRFLSKISEYRHY	4.77
HLA-DRB1*11:01	61	77	17	GVCIMCLRFLSKISEYR	4.77
HLA-DRB1*11:01	64	80	17	IMCLRFLSKISEYRHYQ	4.77
HLA-DRB1*11:01	65	81	17	MCLRFLSKISEYRHYQY	4.77
HLA-DRB1*11:01	59	75	17	PYGVCIMCLRFLSKISE	4.77
HLA-DRB1*11:01	62	78	17	VCIMCLRFLSKISEYRH	4.77
HLA-DRB1*11:01	60	76	17	YGVCIMCLRFLSKISEY	4.77
HLA-DPA1*02:01/DPB1*14:01	44	56	13	KFLFTDLRIVYRD	4.83
HLA-DPA1*01:03/DPB1*04:01	62	74	13	VCIMCLRFLSKIS	4.83
HLA-DRB1*15:01	66	81	16	CLRFLSKISEYRHYQY	4.85
HLA-DRB1*15:01	65	80	16	MCLRFLSKISEYRHYQ	4.85
HLA-DRB5*01:01	38	53	16	QRREVYKFLFTDLRIV	4.85
HLA-DQA1*01:01/DQB1*05:01	40	55	16	REVYKFLFTDLRIVYR	4.85
HLA-DQA1*01:01/DQB1*05:01	39	54	16	RREVYKFLFTDLRIVY	4.85
HLA-DPA1*02:01/DPB1*01:01	79	94	16	YQYSLYGKTLEERVKK	4.85
HLA-DRB3*02:02	52	66	15	IVYRDNNPYGVCIMC	4.90
HLA-DRB1*04:01	22	36	15	SVHEIRLQCVQCKKE	4.90
HLA-DRB3*02:02	51	67	17	RIVYRDNNPYGVCIMCL	4.94
HLA-DRB3*02:02	48	64	17	TDLRIVYRDNNPYGVCI	4.94
HLA-DRB5*01:01	113	126	14	EEKERHVNANKRFH	4.95
HLA-DRB1*04:01	24	37	14	HEIRLQCVQCKKEL	4.95
HLA-DRB1*03:01	50	63	14	LRIVYRDNNPYGVC	4.95
HLA-DRB1*04:01	41	56	16	EVYKFLFTDLRIVYRD	4.96
HLA-DPA1*02:01/DPB1*01:01	78	93	16	HYQYSLYGKTLEERVK	4.96
HLA-DPA1*02:01/DPB1*05:01	34	49	16	KKELQRREVYKFLFTD	4.96
HLA-DRB5*01:01	37	52	16	LQRREVYKFLFTDLRI	4.96
HLA-DRB1*12:01	65	80	16	MCLRFLSKISEYRHYQ	4.96
HLA-DRB1*04:01	40	55	16	REVYKFLFTDLRIVYR	4.96
HLA-DRB1*04:01	39	54	16	RREVYKFLFTDLRIVY	4.96
HLA-DRB3*02:02	48	63	16	TDLRIVYRDNNPYGVC	4.96
HLA-DRB1*04:01	42	57	16	VYKFLFTDLRIVYRDN	4.96
HLA-DRB1*04:01	43	58	16	YKFLFTDLRIVYRDNN	4.96
HLA-DRB5*01:01	43	58	16	YKFLFTDLRIVYRDNN	4.96
HLA-DPA1*02:01/DPB1*01:01	43	55	13	YKFLFTDLRIVYR	4.99
HLA-DPA1*02:01/DPB1*05:01	33	47	15	CKKELQRREVYKFLF	5.00
HLA-DPA1*02:01/DPB1*01:01	80	94	15	QYSLYGKTLEERVKK	5.00
HLA-DRB1*04:01	23	37	15	VHEIRLQCVQCKKEL	5.00

Note: A low adjusted rank (AR) showed a good binder, and only epitopes with AR⩽5 were selected.

Table S8 Prediction results for HPV-52 E6 variant sequence HLA-II epitope peptides

allele	start	end	length	peptide	adjusted rank (AR)
HLA-DPA1*01:03/DPB1*04:01	42	54	13	VYKFLFTDLRIVY	0.08
HLA-DPA1*01:03/DPB1*04:01	41	54	14	EVYKFLFTDLRIVY	0.09
HLA-DPA1*01:03/DPB1*04:01	40	53	14	REVYKFLFTDLRIV	0.11
HLA-DPA1*01:03/DPB1*04:01	43	55	13	YKFLFTDLRIVYR	0.12
HLA-DPA1*01:03/DPB1*04:01	42	55	14	VYKFLFTDLRIVYR	0.13
HLA-DPA1*01:03/DPB1*04:01	41	53	13	EVYKFLFTDLRIV	0.14
HLA-DPA1*01:03/DPB1*04:01	39	53	15	RREVYKFLFTDLRIV	0.15
HLA-DPA1*01:03/DPB1*04:01	42	53	12	VYKFLFTDLRIV	0.15
HLA-DRB1*03:01	42	56	15	VYKFLFTDLRIVYRD	0.15
HLA-DPA1*01:03/DPB1*04:01	43	54	12	YKFLFTDLRIVY	0.15
HLA-DRB1*03:01	43	57	15	YKFLFTDLRIVYRDN	0.15
HLA-DRB1*03:01	41	55	15	EVYKFLFTDLRIVYR	0.17
HLA-DPA1*01:03/DPB1*04:01	40	54	15	REVYKFLFTDLRIVY	0.17
HLA-DPA1*01:03/DPB1*04:01	41	55	15	EVYKFLFTDLRIVYR	0.18
HLA-DPA1*01:03/DPB1*04:01	39	54	16	RREVYKFLFTDLRIVY	0.22
HLA-DRB1*03:01	41	56	16	EVYKFLFTDLRIVYRD	0.24
HLA-DRB1*03:01	40	55	16	REVYKFLFTDLRIVYR	0.24
HLA-DRB1*03:01	42	57	16	VYKFLFTDLRIVYRDN	0.24
HLA-DRB1*03:01	43	58	16	YKFLFTDLRIVYRDNN	0.24
HLA-DRB1*03:01	43	56	14	YKFLFTDLRIVYRD	0.26
HLA-DRB1*03:01	42	55	14	VYKFLFTDLRIVYR	0.27
HLA-DPA1*01:03/DPB1*04:01	38	53	16	QRREVYKFLFTDLRIV	0.28
HLA-DPA1*01:03/DPB1*04:01	40	55	16	REVYKFLFTDLRIVYR	0.28
HLA-DPA1*01:03/DPB1*04:01	42	56	15	VYKFLFTDLRIVYRD	0.28
HLA-DPA1*01:03/DPB1*04:01	43	56	14	YKFLFTDLRIVYRD	0.30
HLA-DRB1*03:01	44	58	15	KFLFTDLRIVYRDNN	0.32
HLA-DRB1*03:01	44	59	16	KFLFTDLRIVYRDNNP	0.33
HLA-DPA1*01:03/DPB1*04:01	41	56	16	EVYKFLFTDLRIVYRD	0.36
HLA-DPA1*01:03/DPB1*04:01	39	55	17	RREVYKFLFTDLRIVYR	0.37
HLA-DPA1*01:03/DPB1*04:01	39	52	14	RREVYKFLFTDLRI	0.38
HLA-DPA1*01:03/DPB1*04:01	38	54	17	QRREVYKFLFTDLRIVY	0.39
HLA-DRB1*03:01	44	57	14	KFLFTDLRIVYRDN	0.40
HLA-DRB1*03:01	41	57	17	EVYKFLFTDLRIVYRDN	0.41
HLA-DRB1*03:01	40	56	17	REVYKFLFTDLRIVYRD	0.41
HLA-DRB1*03:01	42	58	17	VYKFLFTDLRIVYRDNN	0.41
HLA-DRB1*03:01	43	59	17	YKFLFTDLRIVYRDNNP	0.41
HLA-DRB1*03:01	43	55	13	YKFLFTDLRIVYR	0.42
HLA-DRB1*03:01	39	55	17	RREVYKFLFTDLRIVYR	0.43
HLA-DPA1*01:03/DPB1*04:01	37	53	17	LQRREVYKFLFTDLRIV	0.44
HLA-DRB1*03:01	44	60	17	KFLFTDLRIVYRDNNPY	0.46
HLA-DPA1*01:03/DPB1*04:01	43	57	15	YKFLFTDLRIVYRDN	0.48

HLA-DRB1*03:01	44	56	13	KFLFTDLRIVYRD	0.50
HLA-DPA1*01:03/DPB1*04:01	42	57	16	VYKFLFTDLRIVYRDN	0.50
HLA-DPA1*01:03/DPB1*04:01	38	52	15	QRREVYKFLFTDLRI	0.52
HLA-DPA1*01:03/DPB1*04:01	40	52	13	REVYKFLFTDLRI	0.53
HLA-DPA1*01:03/DPB1*04:01	40	56	17	REVYKFLFTDLRIVYRD	0.55
HLA-DPA1*01:03/DPB1*04:01	44	55	12	KFLFTDLRIVYR	0.56
HLA-DRB1*03:01	44	55	12	KFLFTDLRIVYR	0.62
HLA-DRB4*01:01	21	34	14	ESVHEIRLQCVQCK	0.66
HLA-DPA1*01:03/DPB1*04:01	41	57	17	EVYKFLFTDLRIVYRDN	0.70
HLA-DPA1*01:03/DPB1*04:01	37	52	16	LQRREVYKFLFTDLRI	0.80
HLA-DPA1*01:03/DPB1*04:01	38	55	18	QRREVYKFLFTDLRIVYR	0.80
HLA-DPA1*01:03/DPB1*04:01	44	56	13	KFLFTDLRIVYRD	0.81
HLA-DPA1*03:01/DPB1*04:02	40	53	14	REVYKFLFTDLRIV	0.82
HLA-DPA1*01:03/DPB1*04:01	38	51	14	QRREVYKFLFTDLR	0.83
HLA-DPA1*01:03/DPB1*04:01	43	58	16	YKFLFTDLRIVYRDNN	0.83
HLA-DPA1*01:03/DPB1*04:01	39	51	13	RREVYKFLFTDLR	0.84
HLA-DRB4*01:01	20	34	15	EESVHEIRLQCVQCK	0.85
HLA-DRB4*01:01	21	35	15	ESVHEIRLQCVQCKK	0.85
HLA-DPA1*03:01/DPB1*04:02	40	54	15	REVYKFLFTDLRIVY	0.85
HLA-DPA1*03:01/DPB1*04:02	39	53	15	RREVYKFLFTDLRIV	0.87
HLA-DRB1*03:01	41	58	18	EVYKFLFTDLRIVYRDNN	0.89
HLA-DRB1*03:01	44	61	18	KFLFTDLRIVYRDNNPYG	0.89
HLA-DPA1*01:03/DPB1*04:01	37	54	18	LQRREVYKFLFTDLRIVY	0.89
HLA-DRB1*03:01	38	55	18	QRREVYKFLFTDLRIVYR	0.89
HLA-DRB1*03:01	40	57	18	REVYKFLFTDLRIVYRDN	0.89
HLA-DRB1*03:01	39	54	16	RREVYKFLFTDLRIVY	0.89
HLA-DRB1*03:01	39	56	18	RREVYKFLFTDLRIVYRD	0.89
HLA-DRB1*03:01	42	59	18	VYKFLFTDLRIVYRDNNP	0.89
HLA-DRB1*03:01	43	60	18	YKFLFTDLRIVYRDNNPY	0.89
HLA-DRB4*01:01	21	33	13	ESVHEIRLQCVQC	0.90
HLA-DPA1*03:01/DPB1*04:02	42	55	14	VYKFLFTDLRIVYR	0.90
HLA-DPA1*03:01/DPB1*04:02	41	54	14	EVYKFLFTDLRIVY	0.92
HLA-DRB1*03:01	40	54	15	REVYKFLFTDLRIVY	0.92
HLA-DRB4*01:01	20	33	14	EESVHEIRLQCVQC	0.93
HLA-DPA1*03:01/DPB1*04:02	43	56	14	YKFLFTDLRIVYRD	0.96
HLA-DPA1*01:03/DPB1*04:01	37	51	15	LQRREVYKFLFTDLR	0.97
HLA-DPA1*01:03/DPB1*04:01	39	56	18	RREVYKFLFTDLRIVYRD	0.98
HLA-DPA1*02:01/DPB1*05:01	42	55	14	VYKFLFTDLRIVYR	0.99
HLA-DPA1*01:03/DPB1*04:01	42	58	17	VYKFLFTDLRIVYRDNN	0.99
HLA-DPA1*03:01/DPB1*04:02	41	55	15	EVYKFLFTDLRIVYR	1.00
HLA-DPA1*01:03/DPB1*04:01	44	57	14	KFLFTDLRIVYRDN	1.02
HLA-DPA1*01:03/DPB1*04:01	37	50	14	LQRREVYKFLFTDL	1.03
HLA-DPA1*02:01/DPB1*01:01	40	53	14	REVYKFLFTDLRIV	1.04

HLA-DRB1*03:01	45	60	16	FLFTDLRIVYRDNNPY	1.06
HLA-DPA1*03:01/DPB1*04:02	42	54	13	VYKFLFTDLRIVY	1.06
HLA-DPA1*03:01/DPB1*04:02	43	55	13	YKFLFTDLRIVYR	1.08
HLA-DPA1*03:01/DPB1*04:02	41	53	13	EVYKFLFTDLRIV	1.09
HLA-DPA1*03:01/DPB1*04:02	42	56	15	VYKFLFTDLRIVYRD	1.10
HLA-DPA1*03:01/DPB1*04:02	43	57	15	YKFLFTDLRIVYRDN	1.10
HLA-DPA1*01:03/DPB1*04:01	38	50	13	QRREVYKFLFTDL	1.14
HLA-DPA1*01:03/DPB1*04:01	36	53	18	ELQRREVYKFLFTDLRIV	1.15
HLA-DPA1*01:03/DPB1*04:01	41	52	12	EVYKFLFTDLRI	1.16
HLA-DRB1*03:01	41	54	14	EVYKFLFTDLRIVY	1.18
HLA-DRB5*01:01	67	80	14	LRFLSKISEYRHYQ	1.18
HLA-DPA1*02:01/DPB1*01:01	39	52	14	RREVYKFLFTDLRI	1.18
HLA-DRB1*03:01	45	59	15	FLFTDLRIVYRDNNP	1.20
HLA-DRB4*01:01	19	33	15	LEESVHEIRLQCVQC	1.20
HLA-DRB5*01:01	67	79	13	LRFLSKISEYRHY	1.20
HLA-DPA1*02:01/DPB1*01:01	40	54	15	REVYKFLFTDLRIVY	1.20
HLA-DPA1*02:01/DPB1*05:01	40	54	15	REVYKFLFTDLRIVY	1.20
HLA-DPA1*02:01/DPB1*01:01	39	53	15	RREVYKFLFTDLRIV	1.20
HLA-DPA1*01:03/DPB1*04:01	40	57	18	REVYKFLFTDLRIVYRDN	1.24
HLA-DRB5*01:01	66	79	14	CLRFLSKISEYRHY	1.29
HLA-DPA1*02:01/DPB1*01:01	37	50	14	LQRREVYKFLFTDL	1.29
HLA-DPA1*02:01/DPB1*01:01	38	51	14	QRREVYKFLFTDLR	1.29
HLA-DPA1*02:01/DPB1*05:01	43	56	14	YKFLFTDLRIVYRD	1.29
HLA-DPA1*02:01/DPB1*05:01	41	55	15	EVYKFLFTDLRIVYR	1.30
HLA-DPA1*02:01/DPB1*05:01	42	56	15	VYKFLFTDLRIVYRD	1.30
HLA-DPA1*02:01/DPB1*01:01	39	51	13	RREVYKFLFTDLR	1.31
HLA-DPA1*02:01/DPB1*01:01	38	50	13	QRREVYKFLFTDL	1.33
HLA-DPA1*02:01/DPB1*01:01	40	52	13	REVYKFLFTDLRI	1.33
HLA-DRB1*03:01	42	54	13	VYKFLFTDLRIVY	1.34
HLA-DPA1*01:03/DPB1*04:01	36	50	15	ELQRREVYKFLFTDL	1.40
HLA-DPA1*02:01/DPB1*05:01	41	54	14	EVYKFLFTDLRIVY	1.40
HLA-DPA1*03:01/DPB1*04:02	44	57	14	KFLFTDLRIVYRDN	1.40
HLA-DPA1*02:01/DPB1*01:01	38	52	15	QRREVYKFLFTDLRI	1.40
HLA-DRB1*03:01	38	54	17	QRREVYKFLFTDLRIVY	1.40
HLA-DPA1*03:01/DPB1*04:02	42	53	12	VYKFLFTDLRIV	1.42
HLA-DPA1*02:01/DPB1*05:01	43	55	13	YKFLFTDLRIVYR	1.42
HLA-DPA1*03:01/DPB1*04:02	43	54	12	YKFLFTDLRIVY	1.45
HLA-DPA1*03:01/DPB1*04:02	41	56	16	EVYKFLFTDLRIVYRD	1.50
HLA-DRB1*03:01	45	61	17	FLFTDLRIVYRDNNPYG	1.50
HLA-DPA1*01:03/DPB1*04:01	44	58	15	KFLFTDLRIVYRDNN	1.50
HLA-DPA1*03:01/DPB1*04:02	40	55	16	REVYKFLFTDLRIVYR	1.50
HLA-DPA1*03:01/DPB1*04:02	39	54	16	RREVYKFLFTDLRIVY	1.50
HLA-DPA1*03:01/DPB1*04:02	42	57	16	VYKFLFTDLRIVYRDN	1.50

HLA-DPA1*01:03/DPB1*04:01	41	58	18	EVYKFLFTDLRIVYRDNN	1.51
HLA-DRB5*01:01	68	80	13	RFLSKISEYRHYQ	1.51
HLA-DPA1*01:03/DPB1*04:01	39	50	12	RREVYKFLFTDL	1.51
HLA-DRB5*01:01	66	78	13	CLRFLSKISEYRH	1.54
HLA-DPA1*01:03/DPB1*04:01	36	52	17	ELQRREVYKFLFTDLRI	1.55
HLA-DRB5*01:01	66	80	15	CLRFLSKISEYRHYQ	1.60
HLA-DPA1*03:01/DPB1*04:02	44	58	15	KFLFTDLRIVYRDNN	1.60
HLA-DPA1*02:01/DPB1*01:01	37	51	15	LQRREVYKFLFTDLR	1.60
HLA-DPA1*02:01/DPB1*05:01	38	52	15	QRREVYKFLFTDLRI	1.60
HLA-DRB4*01:01	20	35	16	EESVHEIRLQCVQCKK	1.62
HLA-DRB4*01:01	21	36	16	ESVHEIRLQCVQCKKE	1.62
HLA-DRB4*01:01	19	34	16	LEESVHEIRLQCVQCK	1.62
HLA-DPA1*03:01/DPB1*04:02	38	53	16	QRREVYKFLFTDLRIV	1.62
HLA-DPA1*02:01/DPB1*05:01	40	53	14	REVYKFLFTDLRIV	1.62
HLA-DPA1*02:01/DPB1*01:01	39	54	16	RREVYKFLFTDLRIVY	1.62
HLA-DRB4*01:01	22	37	16	SVHEIRLQCVQCKKEL	1.62
HLA-DRB4*01:01	23	38	16	VHEIRLQCVQCKKELQ	1.62
HLA-DPA1*01:03/DPB1*04:01	43	59	17	YKFLFTDLRIVYRDNNP	1.62
HLA-DRB1*03:01	43	54	12	YKFLFTDLRIVY	1.66
HLA-DRB5*01:01	67	81	15	LRFLSKISEYRHYQY	1.70
HLA-DPA1*02:01/DPB1*05:01	43	57	15	YKFLFTDLRIVYRDN	1.70
HLA-DPA1*01:03/DPB1*02:01	41	54	14	EVYKFLFTDLRIVY	1.72
HLA-DPA1*03:01/DPB1*04:02	44	56	13	KFLFTDLRIVYRD	1.72
HLA-DRB5*01:01	67	78	12	LRFLSKISEYRH	1.72
HLA-DRB5*01:01	65	78	14	MCLRFLSKISEYRH	1.72
HLA-DPA1*01:03/DPB1*02:01	40	53	14	REVYKFLFTDLRIV	1.72
HLA-DRB5*01:01	68	81	14	RFLSKISEYRHYQY	1.72
HLA-DPA1*01:03/DPB1*02:01	39	52	14	RREVYKFLFTDLRI	1.72
HLA-DPA1*02:01/DPB1*05:01	42	54	13	VYKFLFTDLRIVY	1.72
HLA-DPA1*02:01/DPB1*14:01	42	54	13	VYKFLFTDLRIVY	1.72
HLA-DPA1*01:03/DPB1*02:01	42	55	14	VYKFLFTDLRIVYR	1.72
HLA-DPA1*02:01/DPB1*14:01	43	55	13	YKFLFTDLRIVYR	1.72
HLA-DPA1*01:03/DPB1*04:01	36	51	16	ELQRREVYKFLFTDLR	1.73
HLA-DPA1*02:01/DPB1*01:01	40	55	16	REVYKFLFTDLRIVYR	1.73
HLA-DPA1*02:01/DPB1*05:01	40	55	16	REVYKFLFTDLRIVYR	1.73
HLA-DRB4*01:01	18	33	16	VLEESVHEIRLQCVQC	1.73
HLA-DRB5*01:01	68	79	12	RFLSKISEYRHY	1.78
HLA-DPA1*02:01/DPB1*01:01	36	50	15	ELQRREVYKFLFTDL	1.80
HLA-DPA1*02:01/DPB1*05:01	37	51	15	LQRREVYKFLFTDLR	1.80
HLA-DRB5*01:01	65	79	15	MCLRFLSKISEYRHY	1.80
HLA-DPA1*02:01/DPB1*05:01	39	53	15	RREVYKFLFTDLRIV	1.80
HLA-DRB1*03:01	45	58	14	FLFTDLRIVYRDNN	1.83
HLA-DQA1*04:01/DQB1*04:02	9	22	14	PRTLHELCEVLEES	1.83

HLA-DPA1*02:01/DPB1*14:01	42	55	14	VYKFLFTDLRIVYR	1.83
HLA-DPA1*02:01/DPB1*01:01	41	56	16	EVYKFLFTDLRIVYRD	1.85
HLA-DPA1*02:01/DPB1*05:01	41	56	16	EVYKFLFTDLRIVYRD	1.85
HLA-DPA1*02:01/DPB1*01:01	38	53	16	QRREVYKFLFTDLRIV	1.85
HLA-DPA1*02:01/DPB1*05:01	39	54	16	RREVYKFLFTDLRIVY	1.85
HLA-DPA1*02:01/DPB1*05:01	42	57	16	VYKFLFTDLRIVYRDN	1.85
HLA-DQA1*04:01/DQB1*04:02	10	22	13	RTLHELCEVLEES	1.87
HLA-DPA1*01:03/DPB1*02:01	41	55	15	EVYKFLFTDLRIVYR	1.90
HLA-DPA1*01:03/DPB1*02:01	38	52	15	QRREVYKFLFTDLRI	1.90
HLA-DPA1*01:03/DPB1*02:01	40	54	15	REVYKFLFTDLRIVY	1.90
HLA-DPA1*01:03/DPB1*02:01	39	53	15	RREVYKFLFTDLRIV	1.90
HLA-DPA1*01:03/DPB1*02:01	42	56	15	VYKFLFTDLRIVYRD	1.90
HLA-DRB3*01:01	51	64	14	RIVYRDNNPYGVCI	1.94
HLA-DQA1*04:01/DQB1*04:02	10	23	14	RTLHELCEVLEESV	1.94
HLA-DPA1*03:01/DPB1*04:02	44	59	16	KFLFTDLRIVYRDNNP	1.96
HLA-DPA1*02:01/DPB1*01:01	39	50	12	RREVYKFLFTDL	1.96
HLA-DPA1*02:01/DPB1*01:01	42	57	16	VYKFLFTDLRIVYRDN	1.96
HLA-DPA1*03:01/DPB1*04:02	43	58	16	YKFLFTDLRIVYRDNN	1.96
HLA-DRB1*11:01	61	75	15	GVCIMCLRFLSKISE	2.00
HLA-DRB3*01:01	50	64	15	LRIVYRDNNPYGVCI	2.00
HLA-DPA1*02:01/DPB1*14:01	43	54	12	YKFLFTDLRIVY	2.02
HLA-DRB1*11:01	63	76	14	CIMCLRFLSKISEY	2.05
HLA-DPA1*02:01/DPB1*01:01	41	54	14	EVYKFLFTDLRIVY	2.05
HLA-DRB1*11:01	64	77	14	IMCLRFLSKISEYR	2.05
HLA-DRB3*01:01	50	63	14	LRIVYRDNNPYGVC	2.05
HLA-DPA1*02:01/DPB1*05:01	38	51	14	QRREVYKFLFTDLR	2.05
HLA-DRB1*11:01	62	75	14	VCIMCLRFLSKISE	2.05
HLA-DPA1*01:03/DPB1*02:01	43	56	14	YKFLFTDLRIVYRD	2.05
HLA-DPA1*03:01/DPB1*04:02	45	60	16	FLFTDLRIVYRDNNPY	2.08
HLA-DRB1*11:01	63	77	15	CIMCLRFLSKISEYR	2.10
HLA-DRB5*01:01	64	78	15	IMCLRFLSKISEYRH	2.10
HLA-DRB3*01:01	51	65	15	RIVYRDNNPYGVCIM	2.10
HLA-DRB1*11:01	65	78	14	MCLRFLSKISEYRH	2.15
HLA-DPA1*02:01/DPB1*01:01	42	55	14	VYKFLFTDLRIVYR	2.15
HLA-DPA1*01:03/DPB1*02:01	41	53	13	EVYKFLFTDLRIV	2.18
HLA-DPA1*03:01/DPB1*04:02	45	57	13	FLFTDLRIVYRDN	2.18
HLA-DPA1*01:03/DPB1*02:01	40	52	13	REVYKFLFTDLRI	2.18
HLA-DQA1*04:01/DQB1*04:02	11	23	13	TLHELCEVLEESV	2.18
HLA-DPA1*01:03/DPB1*02:01	42	54	13	VYKFLFTDLRIVY	2.18
HLA-DPA1*01:03/DPB1*02:01	43	55	13	YKFLFTDLRIVYR	2.18
HLA-DPA1*02:01/DPB1*01:01	37	52	16	LQRREVYKFLFTDLRI	2.19
HLA-DPA1*02:01/DPB1*05:01	37	52	16	LQRREVYKFLFTDLRI	2.19
HLA-DPA1*02:01/DPB1*05:01	38	53	16	QRREVYKFLFTDLRIV	2.19

HLA-DRB1*11:01	64	78	15	IMCLRFLSKISEYRH	2.20
HLA-DQA1*04:01/DQB1*04:02	9	23	15	PRTLHELCEVLEESV	2.20
HLA-DQA1*04:01/DQB1*04:02	8	22	15	RPRTLHELCEVLEES	2.20
HLA-DRB1*11:01	62	76	15	VCIMCLRFLSKISEY	2.20
HLA-DPA1*03:01/DPB1*04:02	45	58	14	FLFTDLRIVYRDNN	2.26
HLA-DPA1*01:03/DPB1*02:01	37	50	14	LQRREVYKFLFTDL	2.26
HLA-DPA1*01:03/DPB1*02:01	38	51	14	QRREVYKFLFTDLR	2.26
HLA-DRB5*01:01	40	53	14	REVYKFLFTDLRIV	2.26
HLA-DQA1*04:01/DQB1*04:02	11	22	12	TLHELCEVLEES	2.28
HLA-DRB1*11:01	65	79	15	MCLRFLSKISEYRHY	2.30
HLA-DPA1*02:01/DPB1*05:01	36	51	16	ELQRREVYKFLFTDLR	2.31
HLA-DPA1*01:03/DPB1*02:01	41	56	16	EVYKFLFTDLRIVYRD	2.31
HLA-DPA1*01:03/DPB1*04:01	35	50	16	KELQRREVYKFLFTDL	2.31
HLA-DPA1*01:03/DPB1*02:01	37	52	16	LQRREVYKFLFTDLRI	2.31
HLA-DPA1*01:03/DPB1*02:01	38	53	16	QRREVYKFLFTDLRIV	2.31
HLA-DPA1*01:03/DPB1*02:01	40	55	16	REVYKFLFTDLRIVYR	2.31
HLA-DPA1*01:03/DPB1*02:01	39	54	16	RREVYKFLFTDLRIVY	2.31
HLA-DPA1*01:03/DPB1*02:01	42	57	16	VYKFLFTDLRIVYRDN	2.31
HLA-DPA1*01:03/DPB1*04:01	42	59	18	VYKFLFTDLRIVYRDNNP	2.31
HLA-DRB1*11:01	63	75	13	CIMCLRFLSKISE	2.34
HLA-DRB1*11:01	64	76	13	IMCLRFLSKISEY	2.34
HLA-DRB3*01:01	50	62	13	LRIVYRDNNPYGV	2.34
HLA-DRB1*11:01	65	77	13	MCLRFLSKISEYR	2.34
HLA-DRB3*01:01	51	63	13	RIVYRDNNPYGVC	2.34
HLA-DPA1*02:01/DPB1*01:01	42	54	13	VYKFLFTDLRIVY	2.34
HLA-DPA1*02:01/DPB1*05:01	37	50	14	LQRREVYKFLFTDL	2.37
HLA-DPA1*03:01/DPB1*04:02	41	57	17	EVYKFLFTDLRIVYRDN	2.38
HLA-DPA1*03:01/DPB1*04:02	38	54	17	QRREVYKFLFTDLRIVY	2.38
HLA-DPA1*03:01/DPB1*04:02	40	56	17	REVYKFLFTDLRIVYRD	2.38
HLA-DPA1*03:01/DPB1*04:02	39	55	17	RREVYKFLFTDLRIVYR	2.38
HLA-DPA1*03:01/DPB1*04:02	42	58	17	VYKFLFTDLRIVYRDNN	2.38
HLA-DRB3*01:01	49	63	15	DLRIVYRDNNPYGVC	2.40
HLA-DPA1*02:01/DPB1*05:01	36	50	15	ELQRREVYKFLFTDL	2.40
HLA-DPA1*02:01/DPB1*01:01	41	55	15	EVYKFLFTDLRIVYR	2.40
HLA-DRB1*03:01	45	62	18	FLFTDLRIVYRDNNPYGV	2.40
HLA-DPA1*01:03/DPB1*04:01	40	51	12	REVYKFLFTDLR	2.40
HLA-DPA1*02:01/DPB1*01:01	36	51	16	ELQRREVYKFLFTDLR	2.42
HLA-DRB3*01:01	49	62	14	DLRIVYRDNNPYGV	2.48
HLA-DRB5*01:01	41	54	14	EVYKFLFTDLRIVY	2.48
HLA-DRB1*03:01	46	59	14	LFTDLRIVYRDNNP	2.48
HLA-DPA1*02:01/DPB1*05:01	39	52	14	RREVYKFLFTDLRI	2.48
HLA-DRB5*01:01	39	52	14	RREVYKFLFTDLRI	2.48
HLA-DPA1*02:01/DPB1*01:01	79	92	14	YQYSLYGKTLEERV	2.48

HLA-DRB1*03:01	45	57	13	FLFTDLRIVYRDN	2.49
HLA-DPA1*01:03/DPB1*02:01	36	50	15	ELQRREVYKFLFTDL	2.50
HLA-DPA1*01:03/DPB1*02:01	37	51	15	LQRREVYKFLFTDLR	2.50
HLA-DRB3*02:02	50	64	15	LRIVYRDNNPYGVCI	2.50
HLA-DRB3*02:02	51	65	15	RIVYRDNNPYGVCIM	2.50
HLA-DPA1*02:01/DPB1*01:01	35	50	16	KELQRREVYKFLFTDL	2.54
HLA-DRB3*02:02	50	65	16	LRIVYRDNNPYGVCIM	2.54
HLA-DPA1*03:01/DPB1*04:02	37	53	17	LQRREVYKFLFTDLRIV	2.55
HLA-DPA1*02:01/DPB1*01:01	39	55	17	RREVYKFLFTDLRIVYR	2.55
HLA-DPA1*02:01/DPB1*05:01	39	55	17	RREVYKFLFTDLRIVYR	2.55
HLA-DRB4*01:01	18	34	17	VLEESVHEIRLQCVQCK	2.55
HLA-DRB1*03:01	37	54	18	LQRREVYKFLFTDLRIVY	2.57
HLA-DQA1*01:02/DQB1*06:02	20	33	14	EESVHEIRLQCVQC	2.58
HLA-DPA1*02:01/DPB1*14:01	41	54	14	EVYKFLFTDLRIVY	2.58
HLA-DPA1*02:01/DPB1*05:01	35	48	14	KELQRREVYKFLFT	2.58
HLA-DPA1*02:01/DPB1*14:01	42	53	12	VYKFLFTDLRIV	2.58
HLA-DRB5*01:01	41	55	15	EVYKFLFTDLRIVYR	2.60
HLA-DRB5*01:01	40	54	15	REVYKFLFTDLRIVY	2.60
HLA-DPA1*02:01/DPB1*01:01	42	56	15	VYKFLFTDLRIVYRD	2.60
HLA-DPA1*01:03/DPB1*02:01	43	57	15	YKFLFTDLRIVYRDN	2.60
HLA-DPA1*02:01/DPB1*05:01	36	48	13	ELQRREVYKFLFT	2.65
HLA-DPA1*02:01/DPB1*05:01	44	56	13	KFLFTDLRIVYRD	2.65
HLA-DPA1*01:03/DPB1*04:01	44	59	16	KFLFTDLRIVYRDNNP	2.65
HLA-DRB5*01:01	65	80	16	MCLRFLSKISEYRHYQ	2.65
HLA-DPA1*02:01/DPB1*05:01	43	54	12	YKFLFTDLRIVY	2.67
HLA-DQA1*01:02/DQB1*06:02	21	34	14	ESVHEIRLQCVQCK	2.69
HLA-DQA1*01:02/DQB1*06:02	19	32	14	LEESVHEIRLQCVQ	2.69
HLA-DRB1*03:01	46	60	15	LFTDLRIVYRDNNPY	2.70
HLA-DRB5*01:01	68	82	15	RFLSKISEYRHYQYS	2.70
HLA-DRB5*01:01	39	53	15	RREVYKFLFTDLRIV	2.70
HLA-DRB4*01:01	20	36	17	EESVHEIRLQCVQCKKE	2.73
HLA-DRB4*01:01	21	37	17	ESVHEIRLQCVQCKKEL	2.73
HLA-DPA1*03:01/DPB1*04:02	45	56	12	FLFTDLRIVYRD	2.73
HLA-DPA1*03:01/DPB1*04:02	44	55	12	KFLFTDLRIVYR	2.73
HLA-DRB4*01:01	19	35	17	LEESVHEIRLQCVQCKK	2.73
HLA-DPA1*02:01/DPB1*01:01	38	54	17	QRREVYKFLFTDLRIVY	2.73
HLA-DRB4*01:01	22	38	17	SVHEIRLQCVQCKKELQ	2.73
HLA-DRB4*01:01	23	39	17	VHEIRLQCVQCKKELQR	2.73
HLA-DRB5*01:01	64	79	16	IMCLRFLSKISEYRHY	2.77
HLA-DPA1*02:01/DPB1*05:01	43	58	16	YKFLFTDLRIVYRDNN	2.77
HLA-DPA1*02:01/DPB1*05:01	36	49	14	ELQRREVYKFLFTD	2.80
HLA-DPA1*01:03/DPB1*04:01	75	88	14	EYRHYQYSLYGKTL	2.80
HLA-DQA1*01:01/DQB1*05:01	40	53	14	REVYKFLFTDLRIV	2.80

HLA-DQA1*01:01/DQB1*05:01	40	54	15	REVYKFLFTDLRIVY	2.80
HLA-DRB3*01:01	48	62	15	TDLRIVYRDNNPYGV	2.80
HLA-DQA1*04:01/DQB1*04:02	11	24	14	TLHELCEVLEESVH	2.80
HLA-DQA1*04:01/DQB1*04:02	11	25	15	TLHELCEVLEESVHE	2.80
HLA-DQA1*01:02/DQB1*06:02	20	32	13	EESVHEIRLQCVQ	2.81
HLA-DQA1*01:02/DQB1*06:02	21	33	13	ESVHEIRLQCVQC	2.81
HLA-DPA1*02:01/DPB1*01:01	41	53	13	EVYKFLFTDLRIV	2.81
HLA-DRB5*01:01	41	53	13	EVYKFLFTDLRIV	2.81
HLA-DPA1*01:03/DPB1*02:01	38	50	13	QRREVYKFLFTDL	2.81
HLA-DRB5*01:01	40	52	13	REVYKFLFTDLRI	2.81
HLA-DPA1*01:03/DPB1*02:01	39	51	13	RREVYKFLFTDLR	2.81
HLA-DRB1*03:01	45	56	12	FLFTDLRIVYRD	2.82
HLA-DRB1*11:01	63	78	16	CIMCLRFLSKISEYRH	2.89
HLA-DRB5*01:01	66	81	16	CLRFLSKISEYRHYQY	2.89
HLA-DRB1*11:01	61	76	16	GVCIMCLRFLSKISEY	2.89
HLA-DRB1*11:01	64	79	16	IMCLRFLSKISEYRHY	2.89
HLA-DRB1*11:01	65	80	16	MCLRFLSKISEYRHYQ	2.89
HLA-DRB1*11:01	62	77	16	VCIMCLRFLSKISEYR	2.89
HLA-DRB1*11:01	60	75	16	YGVCIMCLRFLSKISE	2.89
HLA-DRB4*01:01	17	33	17	EVLEESVHEIRLQCVQC	2.90
HLA-DPA1*02:01/DPB1*05:01	38	54	17	QRREVYKFLFTDLRIVY	2.90
HLA-DPA1*02:01/DPB1*01:01	40	56	17	REVYKFLFTDLRIVYRD	2.90
HLA-DPA1*02:01/DPB1*05:01	40	56	17	REVYKFLFTDLRIVYRD	2.90
HLA-DPA1*01:03/DPB1*04:01	74	88	15	SEYRHYQYSLYGKTL	2.90
HLA-DPA1*02:01/DPB1*01:01	79	93	15	YQYSLYGKTLEERVR	2.90
HLA-DRB3*01:01	52	65	14	IVYRDNNPYGVCIM	2.91
HLA-DQA1*01:01/DQB1*05:01	41	53	13	EVYKFLFTDLRIV	2.96
HLA-DQA1*01:02/DQB1*06:02	19	31	13	LEESVHEIRLQCV	2.96
HLA-DRB1*03:01	46	58	13	LFTDLRIVYRDNN	2.96
HLA-DPA1*02:01/DPB1*05:01	38	50	13	QRREVYKFLFTDL	2.96
HLA-DQA1*01:01/DQB1*05:01	42	54	13	VYKFLFTDLRIVY	2.96
HLA-DQA1*01:02/DQB1*06:02	20	34	15	EESVHEIRLQCVQCK	3.00
HLA-DPA1*01:03/DPB1*04:01	75	89	15	EYRHYQYSLYGKTLE	3.00
HLA-DPA1*02:01/DPB1*01:01	78	92	15	HYQYSLYGKTLEERV	3.00
HLA-DQA1*01:02/DQB1*06:02	19	33	15	LEESVHEIRLQCVQC	3.00
HLA-DRB5*01:01	38	52	15	QRREVYKFLFTDLRI	3.00
HLA-DQA1*01:02/DQB1*06:02	18	32	15	VLEESVHEIRLQCVQ	3.00
HLA-DPA1*02:01/DPB1*14:01	43	56	14	YKFLFTDLRIVYRD	3.02
HLA-DPA1*02:01/DPB1*01:01	41	57	17	EVYKFLFTDLRIVYRDN	3.07
HLA-DPA1*02:01/DPB1*05:01	41	57	17	EVYKFLFTDLRIVYRDN	3.07
HLA-DPA1*03:01/DPB1*04:02	44	60	17	KFLFTDLRIVYRDNNPY	3.07
HLA-DPA1*02:01/DPB1*01:01	37	53	17	LQRREVYKFLFTDLRIV	3.07
HLA-DPA1*03:01/DPB1*04:02	43	59	17	YKFLFTDLRIVYRDNNP	3.07

HLA-DPA1*03:01/DPB1*04:02	45	59	15	FLFTDLRIVYRDNNP	3.10
HLA-DQA1*04:01/DQB1*04:02	10	24	15	RTLHELCEVLEESVH	3.10
HLA-DRB3*02:02	49	64	16	DLRIVYRDNNPYGVCI	3.12
HLA-DPA1*01:03/DPB1*02:01	36	51	16	ELQRREVYKFLFTDLR	3.12
HLA-DPA1*02:01/DPB1*05:01	41	53	13	EVYKFLFTDLRIV	3.12
HLA-DPA1*02:01/DPB1*14:01	41	53	13	EVYKFLFTDLRIV	3.12
HLA-DQA1*01:01/DQB1*05:01	41	54	14	EVYKFLFTDLRIVY	3.12
HLA-DPA1*02:01/DPB1*01:01	78	91	14	HYQYSLYGKTLEER	3.12
HLA-DPA1*01:03/DPB1*02:01	35	50	16	KELQRREVYKFLFTDL	3.12
HLA-DPA1*02:01/DPB1*05:01	44	57	14	KFLFTDLRIVYRDN	3.12
HLA-DRB5*01:01	67	82	16	LRFLSKISEYRHYQYS	3.12
HLA-DPA1*02:01/DPB1*05:01	40	52	13	REVYKFLFTDLRI	3.12
HLA-DRB3*02:02	51	66	16	RIVYRDNNPYGVCIMC	3.12
HLA-DRB1*12:01	63	77	15	CIMCLRFLSKISEYR	3.15
HLA-DQA1*01:02/DQB1*06:02	21	35	15	ESVHEIRLQCVQCKK	3.20
HLA-DPA1*02:01/DPB1*05:01	35	49	15	KELQRREVYKFLFTD	3.20
HLA-DRB3*01:01	49	64	16	DLRIVYRDNNPYGVCI	3.23
HLA-DRB1*12:01	64	77	14	IMCLRFLSKISEYR	3.23
HLA-DRB3*01:01	50	65	16	LRIVYRDNNPYGVCIM	3.23
HLA-DRB3*02:02	51	64	14	RIVYRDNNPYGVCI	3.23
HLA-DRB3*01:01	51	66	16	RIVYRDNNPYGVCIMC	3.23
HLA-DQA1*01:01/DQB1*05:01	42	55	14	VYKFLFTDLRIVYR	3.23
HLA-DPA1*01:03/DPB1*04:01	76	89	14	YRHYQYSLYGKTLE	3.23
HLA-DPA1*02:01/DPB1*01:01	42	58	17	VYKFLFTDLRIVYRDNN	3.24
HLA-DPA1*01:03/DPB1*02:01	41	52	12	EVYKFLFTDLRI	3.26
HLA-DPA1*02:01/DPB1*05:01	44	55	12	KFLFTDLRIVYR	3.26
HLA-DPA1*02:01/DPB1*14:01	44	55	12	KFLFTDLRIVYR	3.26
HLA-DRB1*11:01	65	76	12	MCLRFLSKISEY	3.26
HLA-DPA1*02:01/DPB1*01:01	40	51	12	REVYKFLFTDLR	3.26
HLA-DPA1*01:03/DPB1*02:01	42	53	12	VYKFLFTDLRIV	3.26
HLA-DQA1*01:01/DQB1*05:01	42	53	12	VYKFLFTDLRIV	3.26
HLA-DPA1*01:03/DPB1*02:01	43	54	12	YKFLFTDLRIVY	3.26
HLA-DRB3*01:01	52	64	13	IVYRDNNPYGVCI	3.27
HLA-DPA1*02:01/DPB1*01:01	79	91	13	YQYSLYGKTLEER	3.27
HLA-DPA1*02:01/DPB1*05:01	34	48	15	KKELQRREVYKFLFT	3.30
HLA-DRB1*13:02	49	62	14	DLRIVYRDNNPYGV	3.34
HLA-DRB1*12:01	64	78	15	IMCLRFLSKISEYRH	3.35
HLA-DRB1*12:01	65	78	14	MCLRFLSKISEYRH	3.39
HLA-DPA1*02:01/DPB1*14:01	41	55	15	EVYKFLFTDLRIVYR	3.40
HLA-DPA1*02:01/DPB1*01:01	77	91	15	RHYQYSLYGKTLEER	3.40
HLA-DPA1*03:01/DPB1*04:02	45	61	17	FLFTDLRIVYRDNNPYG	3.41
HLA-DPA1*01:03/DPB1*04:01	35	51	17	KELQRREVYKFLFTDLR	3.41
HLA-DPA1*02:01/DPB1*05:01	42	58	17	VYKFLFTDLRIVYRDNN	3.41

HLA-DRB5*01:01	64	77	14	IMCLRFLSKISEYR	3.45
HLA-DRB1*13:02	50	63	14	LRIVYRDNNPYGVC	3.45
HLA-DPA1*02:01/DPB1*14:01	40	53	14	REVYKFLFTDLRIV	3.45
HLA-DRB1*04:01	42	55	14	VYKFLFTDLRIVYR	3.45
HLA-DRB1*04:01	43	56	14	YKFLFTDLRIVYRD	3.45
HLA-DRB5*01:01	63	78	16	CIMCLRFLSKISEYRH	3.46
HLA-DPA1*01:03/DPB1*04:01	73	88	16	ISEYRHYQYSLYGKTL	3.46
HLA-DRB1*03:01	46	61	16	LFTDLRIVYRDNNPYG	3.46
HLA-DRB3*01:01	48	63	16	TDLRIVYRDNNPYGVC	3.46
HLA-DPA1*01:03/DPB1*04:01	76	90	15	YRHYQYSLYGKTLEE	3.50
HLA-DRB1*11:01	66	79	14	CLRFLSKISEYRHY	3.55
HLA-DPA1*01:03/DPB1*04:01	35	52	18	KELQRREVYKFLFTDLRI	3.55
HLA-DRB4*01:01	19	32	14	LEESVHEIRLQCVQ	3.55
HLA-DRB4*01:01	22	35	14	SVHEIRLQCVQCKK	3.55
HLA-DRB4*01:01	23	36	14	VHEIRLQCVQCKKE	3.55
HLA-DQA1*01:02/DQB1*06:02	20	31	12	EESVHEIRLQCV	3.56
HLA-DRB3*01:01	51	62	12	RIVYRDNNPYGV	3.56
HLA-DPA1*02:01/DPB1*01:01	36	52	17	ELQRREVYKFLFTDLRI	3.58
HLA-DPA1*02:01/DPB1*05:01	36	52	17	ELQRREVYKFLFTDLRI	3.58
HLA-DPA1*02:01/DPB1*05:01	37	53	17	LQRREVYKFLFTDLRIV	3.58
HLA-DPA1*02:01/DPB1*05:01	37	49	13	LQRREVYKFLFTD	3.59
HLA-DRB1*13:02	50	62	13	LRIVYRDNNPYGV	3.59
HLA-DPA1*02:01/DPB1*01:01	80	92	13	QYSLYGKTLEERV	3.59
HLA-DPA1*02:01/DPB1*05:01	39	51	13	RREVYKFLFTDLR	3.59
HLA-DRB4*01:01	22	34	13	SVHEIRLQCVQCK	3.59
HLA-DRB4*01:01	23	35	13	VHEIRLQCVQCKK	3.59
HLA-DRB1*13:02	50	64	15	LRIVYRDNNPYGVCI	3.60
HLA-DRB1*12:01	65	79	15	MCLRFLSKISEYRHY	3.60
HLA-DQA1*01:01/DQB1*05:01	39	53	15	RREVYKFLFTDLRIV	3.6
HLA-DPA1*02:01/DPB1*01:01	80	93	14	QYSLYGKTLEERVR	3.66
HLA-DPA1*02:01/DPB1*05:01	35	50	16	KELQRREVYKFLFTDL	3.69
HLA-DPA1*01:03/DPB1*02:01	43	58	16	YKFLFTDLRIVYRDNN	3.69
HLA-DRB1*04:01	41	55	15	EVYKFLFTDLRIVYR	3.7
HLA-DRB1*15:01	64	78	15	IMCLRFLSKISEYRH	3.70
HLA-DRB1*04:01	40	54	15	REVYKFLFTDLRIVY	3.70
HLA-DRB1*04:01	42	56	15	VYKFLFTDLRIVYRD	3.70
HLA-DRB1*04:01	43	57	15	YKFLFTDLRIVYRDN	3.70
HLA-DRB1*11:01	66	78	13	CLRFLSKISEYRH	3.74
HLA-DRB5*01:01	65	77	13	MCLRFLSKISEYR	3.74
HLA-DPA1*01:03/DPB1*04:01	76	88	13	YRHYQYSLYGKTL	3.74
HLA-DPA1*01:03/DPB1*02:01	36	52	17	ELQRREVYKFLFTDLRI	3.75
HLA-DPA1*01:03/DPB1*02:01	41	57	17	EVYKFLFTDLRIVYRDN	3.75
HLA-DPA1*01:03/DPB1*02:01	37	53	17	LQRREVYKFLFTDLRIV	3.75

HLA-DPA1*01:03/DPB1*02:01	38	54	17	QRREVYKFLFTDLRIVY	3.75
HLA-DPA1*01:03/DPB1*02:01	40	56	17	REVYKFLFTDLRIVYRD	3.75
HLA-DPA1*01:03/DPB1*02:01	39	55	17	RREVYKFLFTDLRIVYR	3.75
HLA-DPA1*01:03/DPB1*02:01	42	58	17	VYKFLFTDLRIVYRDNN	3.75
HLA-DRB1*04:01	41	54	14	EVYKFLFTDLRIVY	3.77
HLA-DPA1*02:01/DPB1*05:01	34	47	14	KKELQRREVYKFLF	3.77
HLA-DRB3*02:02	50	63	14	LRIVYRDNNPYGVC	3.77
HLA-DRB1*13:02	51	64	14	RIVYRDNNPYGVCI	3.77
HLA-DQA1*01:02/DQB1*06:02	18	31	14	VLEESVHEIRLQCV	3.77
HLA-DQA1*01:01/DQB1*05:01	41	55	15	EVYKFLFTDLRIVYR	3.80
HLA-DRB3*01:01	52	66	15	IVYRDNNPYGVCIMC	3.80
HLA-DQA1*01:01/DQB1*05:01	42	56	15	VYKFLFTDLRIVYRD	3.80
HLA-DRB1*11:01	66	81	16	CLRFLSKISEYRHYQY	3.81
HLA-DQA1*04:01/DQB1*04:02	8	23	16	RPRTLHELCEVLEESV	3.81
HLA-DPA1*01:03/DPB1*04:01	74	89	16	SEYRHYQYSLYGKTLE	3.81
HLA-DQA1*01:02/DQB1*06:02	21	32	12	ESVHEIRLQCVQ	3.85
HLA-DRB5*01:01	41	52	12	EVYKFLFTDLRI	3.85
HLA-DPA1*01:03/DPB1*04:01	43	60	18	YKFLFTDLRIVYRDNNPY	3.85
HLA-DRB4*01:01	20	32	13	EESVHEIRLQCVQ	3.90
HLA-DRB4*01:01	23	37	15	VHEIRLQCVQCKKEL	3.90
HLA-DPA1*02:01/DPB1*01:01	35	51	17	KELQRREVYKFLFTDLR	3.92
HLA-DRB1*11:01	67	82	16	LRFLSKISEYRHYQYS	3.92
HLA-DRB3*02:02	50	66	17	LRIVYRDNNPYGVCIMC	3.92
HLA-DQA1*04:01/DQB1*04:02	10	25	16	RTLHELCEVLEESVHE	3.92
HLA-DQA1*04:01/DQB1*04:02	7	22	16	TRPRTLHELCEVLEES	3.92
HLA-DPA1*01:03/DPB1*04:01	60	73	14	YGVCIMCLRFLSKI	3.98
HLA-DPA1*02:01/DPB1*14:01	40	54	15	REVYKFLFTDLRIVY	4.00
HLA-DRB1*13:02	48	62	15	TDLRIVYRDNNPYGV	4.00
HLA-DPA1*02:01/DPB1*14:01	42	56	15	VYKFLFTDLRIVYRD	4.00
HLA-DRB3*01:01	47	62	16	FTDLRIVYRDNNPYGV	4.04
HLA-DPA1*02:01/DPB1*05:01	35	47	13	KELQRREVYKFLF	4.05
HLA-DRB3*02:02	49	65	17	DLRIVYRDNNPYGVCIM	4.09
HLA-DPA1*02:01/DPB1*05:01	35	51	17	KELQRREVYKFLFTDLR	4.09
HLA-DRB5*01:01	63	77	15	CIMCLRFLSKISEYR	4.10
HLA-DRB3*02:02	49	63	15	DLRIVYRDNNPYGVC	4.10
HLA-DRB4*01:01	22	36	15	SVHEIRLQCVQCKKE	4.10
HLA-DRB4*01:01	18	32	15	VLEESVHEIRLQCVQ	4.10
HLA-DRB1*11:01	64	75	12	IMCLRFLSKISE	4.15
HLA-DPA1*02:01/DPB1*01:01	42	53	12	VYKFLFTDLRIV	4.15
HLA-DQA1*04:01/DQB1*04:02	9	24	16	PRTLHELCEVLEESVH	4.16
HLA-DRB1*13:02	49	63	15	DLRIVYRDNNPYGVC	4.20
HLA-DPA1*02:01/DPB1*05:01	44	58	15	KFLFTDLRIVYRDNN	4.20
HLA-DRB1*12:01	67	81	15	LRFLSKISEYRHYQY	4.20

HLA-DRB1*12:01	63	78	16	CIMCLRFLSKISEYRH	4.27
HLA-DPA1*01:03/DPB1*04:01	75	90	16	EYRHYQYSLYGKTLEE	4.27
HLA-DRB1*12:01	64	79	16	IMCLRFLSKISEYRHY	4.27
HLA-DRB1*11:01	66	80	15	CLRFLSKISEYRHYQ	4.30
HLA-DRB1*12:01	65	77	13	MCLRFLSKISEYR	4.37
HLA-DRB1*13:02	51	63	13	RIVYRDNNPYGVC	4.37
HLA-DRB1*04:01	42	54	13	VYKFLFTDLRIVY	4.37
HLA-DRB1*04:01	43	55	13	YKFLFTDLRIVYR	4.37
HLA-DRB1*12:01	62	77	16	VCIMCLRFLSKISEYR	4.39
HLA-DRB1*13:02	51	65	15	RIVYRDNNPYGVCIM	4.40
HLA-DPA1*01:03/DPB1*02:01	44	57	14	KFLFTDLRIVYRDN	4.41
HLA-DPA1*03:01/DPB1*04:02	38	55	18	QRREVYKFLFTDLRIVYR	4.44
HLA-DPA1*03:01/DPB1*04:02	39	56	18	RREVYKFLFTDLRIVYRD	4.44
HLA-DPA1*02:01/DPB1*05:01	37	48	12	LQRREVYKFLFT	4.45
HLA-DPA1*01:03/DPB1*02:01	39	50	12	RREVYKFLFTDL	4.45
HLA-DRB4*01:01	23	34	12	VHEIRLQCVQCK	4.45
HLA-DRB1*15:01	64	79	16	IMCLRFLSKISEYRHY	4.50
HLA-DRB5*01:01	40	55	16	REVYKFLFTDLRIVYR	4.50
HLA-DQA1*04:01/DQB1*04:02	11	26	16	TLHELCEVLEESVHEI	4.5
HLA-DRB1*12:01	66	79	14	CLRFLSKISEYRHY	4.52
HLA-DPA1*01:03/DPB1*04:01	61	74	14	GVCIMCLRFLSKIS	4.52
HLA-DPA1*01:03/DPB1*02:01	44	56	13	KFLFTDLRIVYRD	4.52
HLA-DRB5*01:01	64	80	17	IMCLRFLSKISEYRHYQ	4.60
HLA-DPA1*01:03/DPB1*04:01	34	50	17	KKELQRREVYKFLFTDL	4.60
HLA-DPA1*02:01/DPB1*01:01	34	50	17	KKELQRREVYKFLFTDL	4.60
HLA-DRB5*01:01	68	83	16	RFLSKISEYRHYQYSL	4.62
HLA-DPA1*02:01/DPB1*01:01	76	91	16	YRHYQYSLYGKTLEER	4.62
HLA-DRB5*01:01	114	127	14	EKERHVNANKRFHN	4.63
HLA-DRB1*04:01	23	36	14	VHEIRLQCVQCKKE	4.63
HLA-DPA1*02:01/DPB1*01:01	43	56	14	YKFLFTDLRIVYRD	4.63
HLA-DPA1*01:03/DPB1*04:01	61	73	13	GVCIMCLRFLSKI	4.68
HLA-DRB5*01:01	41	56	16	EVYKFLFTDLRIVYRD	4.73
HLA-DRB5*01:01	39	54	16	RREVYKFLFTDLRIVY	4.73
HLA-DRB5*01:01	42	57	16	VYKFLFTDLRIVYRDN	4.73
HLA-DPA1*02:01/DPB1*01:01	79	94	16	YQYSLYGKTLEERVRK	4.73
HLA-DPA1*01:03/DPB1*04:01	76	91	16	YRHYQYSLYGKTLEER	4.73
HLA-DRB1*11:01	66	77	12	CLRFLSKISEYR	4.74
HLA-DRB5*01:01	66	77	12	CLRFLSKISEYR	4.74
HLA-DPA1*03:01/DPB1*04:02	41	58	18	EVYKFLFTDLRIVYRDNN	4.74
HLA-DRB3*01:01	52	63	12	IVYRDNNPYGVC	4.74
HLA-DPA1*01:03/DPB1*02:01	44	55	12	KFLFTDLRIVYR	4.74
HLA-DRB1*03:01	46	57	12	LFTDLRIVYRDN	4.74
HLA-DPA1*03:01/DPB1*04:02	37	54	18	LQRREVYKFLFTDLRIVY	4.74

HLA-DPA1*02:01/DPB1*05:01	38	55	18	QRREVYKFLFTDLRIVYR	4.74
HLA-DPA1*03:01/DPB1*04:02	40	57	18	REVYKFLFTDLRIVYRDN	4.74
HLA-DQA1*04:01/DQB1*04:02	8	21	14	RPRTLHELCEVLEE	4.74
HLA-DPA1*03:01/DPB1*04:02	39	52	14	RREVYKFLFTDLRI	4.74
HLA-DPA1*02:01/DPB1*05:01	42	53	12	VYKFLFTDLRIV	4.74
HLA-DPA1*03:01/DPB1*04:02	42	59	18	VYKFLFTDLRIVYRDNNP	4.74
HLA-DRB1*11:01	63	79	17	CIMCLRFLSKISEYRHY	4.77
HLA-DRB1*11:01	61	77	17	GVCIMCLRFLSKISEYR	4.77
HLA-DRB1*11:01	64	80	17	IMCLRFLSKISEYRHYQ	4.77
HLA-DRB1*11:01	65	81	17	MCLRFLSKISEYRHYQY	4.77
HLA-DRB1*11:01	59	75	17	PYGVCIMCLRFLSKISE	4.77
HLA-DRB1*11:01	62	78	17	VCIMCLRFLSKISEYRH	4.77
HLA-DRB1*11:01	60	76	17	YGVCIMCLRFLSKISEY	4.77
HLA-DPA1*02:01/DPB1*14:01	44	56	13	KFLFTDLRIVYRD	4.83
HLA-DPA1*01:03/DPB1*04:01	62	74	13	VCIMCLRFLSKIS	4.83
HLA-DRB1*15:01	66	81	16	CLRFLSKISEYRHYQY	4.85
HLA-DRB1*15:01	65	80	16	MCLRFLSKISEYRHYQ	4.85
HLA-DRB5*01:01	38	53	16	QRREVYKFLFTDLRIV	4.85
HLA-DQA1*01:01/DQB1*05:01	40	55	16	REVYKFLFTDLRIVYR	4.85
HLA-DQA1*01:01/DQB1*05:01	39	54	16	RREVYKFLFTDLRIVY	4.85
HLA-DRB3*02:02	52	66	15	IVYRDNNPYGVCIMC	4.90
HLA-DPA1*02:01/DPB1*01:01	80	94	15	QYSLYGKTLEERVRK	4.90
HLA-DRB1*04:01	22	36	15	SVHEIRLQCVQCKKE	4.90
HLA-DRB3*02:02	51	67	17	RIVYRDNNPYGVCIMCL	4.94
HLA-DRB3*02:02	48	64	17	TDLRIVYRDNNPYGVCI	4.94
HLA-DRB5*01:01	113	126	14	EEKERHVNANKRFH	4.95
HLA-DRB1*04:01	24	37	14	HEIRLQCVQCKKEL	4.95
HLA-DRB1*03:01	50	63	14	LRIVYRDNNPYGVC	4.95
HLA-DRB1*04:01	41	56	16	EVYKFLFTDLRIVYRD	4.96
HLA-DPA1*02:01/DPB1*01:01	78	93	16	HYQYSLYGKTLEERVR	4.96
HLA-DPA1*02:01/DPB1*05:01	34	49	16	KKELQRREVYKFLFTD	4.96
HLA-DRB5*01:01	37	52	16	LQRREVYKFLFTDLRI	4.96
HLA-DRB1*12:01	65	80	16	MCLRFLSKISEYRHYQ	4.96
HLA-DRB1*04:01	40	55	16	REVYKFLFTDLRIVYR	4.96
HLA-DRB1*04:01	39	54	16	RREVYKFLFTDLRIVY	4.96
HLA-DRB3*02:02	48	63	16	TDLRIVYRDNNPYGVC	4.96
HLA-DRB1*04:01	42	57	16	VYKFLFTDLRIVYRDN	4.96
HLA-DRB1*04:01	43	58	16	YKFLFTDLRIVYRDNN	4.96
HLA-DRB5*01:01	43	58	16	YKFLFTDLRIVYRDNN	4.96
HLA-DPA1*02:01/DPB1*01:01	43	55	13	YKFLFTDLRIVYR	4.99
HLA-DPA1*02:01/DPB1*05:01	33	47	15	CKKELQRREVYKFLF	5.00
HLA-DRB1*04:01	23	37	15	VHEIRLQCVQCKKEL	5.00

Note: A low adjusted rank (AR) showed a good binder，and only epitopes with AR≤5 were selected.
