# Supplementary material for: The genetic variability, phylogeny and functional significance of E6, E7 and LCR in human papillomavirus type 52 isolates in Sichuan, China
Source: Virol J. 2021 May 3;18:94. doi: 10.1186/s12985-021-01565-5 (PMC8091156; doi:10.1186/s12985-021-01565-5)
Supplement: Supplementary file 5 — Additional file 5: Table S9. Prediction results for HPV-52 E7 reference sequence HLA-II epitope peptides. Table S10. Prediction results for HPV-52 E7 variant sequence HLA-II epitope peptides. [file 12985_2021_1565_MOESM5_ESM.pdf]

Table S9 Prediction results for HPV-52 E7 reference sequence HLA-II epitope peptides

allele	start	end	length	peptide	adjusted rank (AR)
HLA-DQA1*03:01/DQB1*03:02	6	19	14	ATIKDYILDLQPET	0.44
HLA-DQA1*03:01/DQB1*03:02	7	20	14	TIKDYILDLQPETT	0.45
HLA-DQA1*03:01/DQB1*03:02	8	21	14	IKDYILDLQPETTD	0.48
HLA-DQA1*03:01/DQB1*03:02	7	19	13	TIKDYILDLQPET	0.50
HLA-DQA1*03:01/DQB1*03:02	5	18	14	KATIKDYILDLQPE	0.51
HLA-DQA1*03:01/DQB1*03:02	8	19	12	IKDYILDLQPET	0.53
HLA-DQA1*03:01/DQB1*03:02	5	19	15	KATIKDYILDLQPET	0.53
HLA-DQA1*03:01/DQB1*03:02	6	20	15	ATIKDYILDLQPETT	0.55
HLA-DQA1*03:01/DQB1*03:02	7	21	15	TIKDYILDLQPETTD	0.58
HLA-DQA1*03:01/DQB1*03:02	8	20	13	IKDYILDLQPETT	0.59
HLA-DQA1*03:01/DQB1*03:02	4	18	15	DKATIKDYILDLQPE	0.60
HLA-DQA1*03:01/DQB1*03:02	6	18	13	ATIKDYILDLQPE	0.61
HLA-DQA1*03:01/DQB1*03:02	7	18	12	TIKDYILDLQPE	0.62
HLA-DQA1*03:01/DQB1*03:02	8	22	15	IKDYILDLQPETTDL	0.64
HLA-DQA1*03:01/DQB1*03:02	5	20	16	KATIKDYILDLQPETT	0.87
HLA-DQA1*03:01/DQB1*03:02	6	21	16	ATIKDYILDLQPETTD	0.88
HLA-DQA1*03:01/DQB1*03:02	4	19	16	DKATIKDYILDLQPET	0.91
HLA-DQA1*03:01/DQB1*03:02	3	18	16	GDKATIKDYILDLQPE	0.92
HLA-DQA1*03:01/DQB1*03:02	7	22	16	TIKDYILDLQPETTDL	1.03
HLA-DQA1*03:01/DQB1*03:02	8	23	16	IKDYILDLQPETTDLH	1.15
HLA-DQA1*03:01/DQB1*03:02	3	19	17	GDKATIKDYILDLQPET	1.53
HLA-DQA1*03:01/DQB1*03:02	2	18	17	RGDKATIKDYILDLQPE	1.65
HLA-DQA1*03:01/DQB1*03:02	9	24	16	KDYILDLQPETTDLHC	1.73
HLA-DRB3*01:01	6	19	14	ATIKDYILDLQPET	1.83
HLA-DRB3*01:01	8	21	14	IKDYILDLQPETTD	1.83
HLA-DRB3*01:01	9	22	14	KDYILDLQPETTDL	1.83
HLA-DRB3*01:01	7	20	14	TIKDYILDLQPETT	1.83
HLA-DQA1*03:01/DQB1*03:02	4	20	17	DKATIKDYILDLQPETT	1.87
HLA-DRB3*01:01	8	20	13	IKDYILDLQPETT	1.87
HLA-DQA1*03:01/DQB1*03:02	5	21	17	KATIKDYILDLQPETTD	1.87
HLA-DRB3*01:01	9	21	13	KDYILDLQPETTD	1.87
HLA-DRB3*01:01	7	19	13	TIKDYILDLQPET	1.87
HLA-DQA1*05:01/DQB1*02:01	8	21	14	IKDYILDLQPETTD	1.94
HLA-DRB3*01:01	8	22	15	IKDYILDLQPETTDL	2.00
HLA-DRB3*01:01	9	23	15	KDYILDLQPETTDLH	2.00
HLA-DQA1*05:01/DQB1*02:01	7	21	15	TIKDYILDLQPETTD	2.00
HLA-DRB3*01:01	7	21	15	TIKDYILDLQPETTD	2.00
HLA-DQA1*05:01/DQB1*02:01	7	20	14	TIKDYILDLQPETT	2.05
HLA-DRB3*01:01	6	20	15	ATIKDYILDLQPETT	2.10
HLA-DRB3*01:01	5	19	15	KATIKDYILDLQPET	2.10
HLA-DQA1*03:01/DQB1*03:02	6	22	17	ATIKDYILDLQPETTDL	2.21

HLA-DQA1*05:01/DQB1*02:01	8	22	15	IKDYILDLQPETTDL	2.30
HLA-DQA1*03:01/DQB1*03:02	7	23	17	TIKDYILDLQPETTDLH	2.38
HLA-DQA1*05:01/DQB1*02:01	6	20	15	ATIKDYILDLQPETT	2.40
HLA-DQA1*05:01/DQB1*02:01	9	22	14	KDYILDLQPETTDL	2.48
HLA-DQA1*03:01/DQB1*03:02	8	24	17	IKDYILDLQPETTDLHC	2.55
HLA-DRB3*01:01	8	19	12	IKDYILDLQPET	2.58
HLA-DRB3*01:01	9	20	12	KDYILDLQPETT	2.67
HLA-DQA1*05:01/DQB1*02:01	6	21	16	ATIKDYILDLQPETTD	2.77
HLA-DQA1*05:01/DQB1*02:01	7	22	16	TIKDYILDLQPETTDL	2.77
HLA-DQA1*05:01/DQB1*02:01	8	20	13	IKDYILDLQPETT	2.81
HLA-DQA1*05:01/DQB1*02:01	9	21	13	KDYILDLQPETTD	2.96
HLA-DQA1*01:01/DQB1*05:01	5	18	14	KATIKDYILDLQPE	3.12
HLA-DQA1*03:01/DQB1*03:02	9	22	14	KDYILDLQPETTDL	3.12
HLA-DQA1*03:01/DQB1*03:02	10	23	14	DYILDLQPETTDLH	3.23
HLA-DRB3*01:01	8	23	16	IKDYILDLQPETTDLH	3.23
HLA-DRB3*01:01	7	22	16	TIKDYILDLQPETTDL	3.23
HLA-DQA1*03:01/DQB1*03:02	2	19	18	RGDKATIKDYILDLQPET	3.26
HLA-DQA1*05:01/DQB1*02:01	6	19	14	ATIKDYILDLQPET	3.34
HLA-DRB3*01:01	6	21	16	ATIKDYILDLQPETTD	3.35
HLA-DQA1*05:01/DQB1*02:01	5	20	16	KATIKDYILDLQPETT	3.35
HLA-DRB3*01:01	9	24	16	KDYILDLQPETTDLHC	3.35
HLA-DQA1*05:01/DQB1*02:01	9	23	15	KDYILDLQPETTDLH	3.40
HLA-DRB3*01:01	4	19	16	DKATIKDYILDLQPET	3.46
HLA-DRB3*01:01	10	25	16	DYILDLQPETTDLHCY	3.46
HLA-DRB3*01:01	5	20	16	KATIKDYILDLQPETT	3.46
HLA-DQA1*03:01/DQB1*03:02	1	18	18	MRGDKATIKDYILDLQPE	3.55
HLA-DQA1*03:01/DQB1*03:02	10	25	16	DYILDLQPETTDLHCY	3.58
HLA-DQA1*03:01/DQB1*03:02	9	25	17	KDYILDLQPETTDLHCY	3.58
HLA-DQA1*03:01/DQB1*03:02	10	22	13	DYILDLQPETTDL	3.59
HLA-DQA1*03:01/DQB1*03:02	9	21	13	KDYILDLQPETTD	3.59
HLA-DQA1*01:01/DQB1*05:01	4	18	15	DKATIKDYILDLQPE	3.60
HLA-DQA1*05:01/DQB1*02:01	8	23	16	IKDYILDLQPETTDLH	3.69
HLA-DQA1*01:01/DQB1*05:01	5	19	15	KATIKDYILDLQPET	3.70
HLA-DPA1*03:01/DPB1*04:02	78	93	16	LRTLQQMLLGTLQVVC	3.81
HLA-DQA1*03:01/DQB1*03:02	4	21	18	DKATIKDYILDLQPETTD	3.85
HLA-DQA1*03:01/DQB1*03:02	3	20	18	GDKATIKDYILDLQPETT	3.85
HLA-DQA1*03:01/DQB1*03:02	9	23	15	KDYILDLQPETTDLH	3.90
HLA-DQA1*05:01/DQB1*02:01	7	19	13	TIKDYILDLQPET	3.90
HLA-DPA1*03:01/DPB1*04:02	77	92	16	DLRTLQQMLLGTLQVV	3.92
HLA-DQA1*03:01/DQB1*03:02	10	24	15	DYILDLQPETTDLHC	4.00
HLA-DQA1*01:01/DQB1*05:01	5	17	13	KATIKDYILDLQP	4.05
HLA-DQA1*01:01/DQB1*05:01	4	17	14	DKATIKDYILDLQP	4.09
HLA-DQA1*03:01/DQB1*03:02	10	21	12	DYILDLQPETTD	4.15

HLA-DQA1*03:01/DQB1*03:02	9	20	12	KDYILDLQPETT	4.15
HLA-DRB4*01:01	77	90	14	DLRTLQQMLLGTLQ	4.20
HLA-DQA1*05:01/DQB1*02:01	5	19	15	KATIKDYILDLQPET	4.20
HLA-DRB4*01:01	76	89	14	TDLRTLQQMLLGTL	4.20
HLA-DPA1*03:01/DPB1*04:02	78	92	15	LRTLQQMLLGTLQVV	4.30
HLA-DQA1*01:01/DQB1*05:01	4	16	13	DKATIKDYILDLQ	4.37
HLA-DQA1*01:01/DQB1*05:01	3	16	14	GDKATIKDYILDLQ	4.41
HLA-DQA1*05:01/DQB1*02:01	6	22	17	ATIKDYILDLQPETTDL	4.43
HLA-DQA1*05:01/DQB1*02:01	5	21	17	KATIKDYILDLQPETTD	4.43
HLA-DQA1*03:01/DQB1*03:02	5	22	18	KATIKDYILDLQPETTDL	4.44
HLA-DPA1*03:01/DPB1*04:02	78	91	14	LRTLQQMLLGTLQV	4.52
HLA-DQA1*05:01/DQB1*02:01	9	20	12	KDYILDLQPETT	4.74
HLA-DQA1*01:01/DQB1*05:01	3	17	15	GDKATIKDYILDLQP	4.8
HLA-DRB4*01:01	77	89	13	DLRTLQQMLLGTL	4.83
HLA-DQA1*05:01/DQB1*02:01	9	24	16	KDYILDLQPETTDLHC	4.85
HLA-DQA1*01:01/DQB1*05:01	6	19	14	ATIKDYILDLQPET	4.95
HLA-DRB4*01:01	76	90	15	TDLRTLQQMLLGTLQ	5.00

Note: A low adjusted rank (AR) showed a good binder，and only epitopes with AR≤5 were selected.

Table S10 Prediction results for HPV-52 E7 variant sequence HLA-II epitope peptides

allele	start	end	length	peptide	adjusted rank (AR)
HLA-DQA1*03:01/DQB1*03:02	6	19	14	ATIKDYILDLQPET	0.44
HLA-DQA1*03:01/DQB1*03:02	7	20	14	TIKDYILDLQPETT	0.45
HLA-DQA1*03:01/DQB1*03:02	8	21	14	IKDYILDLQPETTD	0.48
HLA-DQA1*03:01/DQB1*03:02	7	19	13	TIKDYILDLQPET	0.5
HLA-DQA1*03:01/DQB1*03:02	5	18	14	KATIKDYILDLQPE	0.51
HLA-DQA1*03:01/DQB1*03:02	8	19	12	IKDYILDLQPET	0.53
HLA-DQA1*03:01/DQB1*03:02	5	19	15	KATIKDYILDLQPET	0.53
HLA-DQA1*03:01/DQB1*03:02	6	20	15	ATIKDYILDLQPETT	0.55
HLA-DQA1*03:01/DQB1*03:02	7	21	15	TIKDYILDLQPETTD	0.58
HLA-DQA1*03:01/DQB1*03:02	8	20	13	IKDYILDLQPETT	0.59
HLA-DQA1*03:01/DQB1*03:02	4	18	15	DKATIKDYILDLQPE	0.60
HLA-DQA1*03:01/DQB1*03:02	6	18	13	ATIKDYILDLQPE	0.61
HLA-DQA1*03:01/DQB1*03:02	7	18	12	TIKDYILDLQPE	0.62
HLA-DQA1*03:01/DQB1*03:02	8	22	15	IKDYILDLQPETTDL	0.64
HLA-DQA1*03:01/DQB1*03:02	5	20	16	KATIKDYILDLQPETT	0.87
HLA-DQA1*03:01/DQB1*03:02	6	21	16	ATIKDYILDLQPETTD	0.88
HLA-DQA1*03:01/DQB1*03:02	4	19	16	DKATIKDYILDLQPET	0.91
HLA-DQA1*03:01/DQB1*03:02	3	18	16	GDKATIKDYILDLQPE	0.92
HLA-DQA1*03:01/DQB1*03:02	7	22	16	TIKDYILDLQPETTDL	1.03
HLA-DQA1*03:01/DQB1*03:02	8	23	16	IKDYILDLQPETTDLH	1.15
HLA-DQA1*03:01/DQB1*03:02	3	19	17	GDKATIKDYILDLQPET	1.53
HLA-DQA1*03:01/DQB1*03:02	2	18	17	RGDKATIKDYILDLQPE	1.65
HLA-DQA1*03:01/DQB1*03:02	9	24	16	KDYILDLQPETTDLHC	1.73
HLA-DRB3*01:01	6	19	14	ATIKDYILDLQPET	1.83
HLA-DRB3*01:01	8	21	14	IKDYILDLQPETTD	1.83
HLA-DRB3*01:01	9	22	14	KDYILDLQPETTDL	1.83
HLA-DRB3*01:01	7	20	14	TIKDYILDLQPETT	1.83
HLA-DQA1*03:01/DQB1*03:02	4	20	17	DKATIKDYILDLQPETT	1.87
HLA-DRB3*01:01	8	20	13	IKDYILDLQPETT	1.87
HLA-DQA1*03:01/DQB1*03:02	5	21	17	KATIKDYILDLQPETTD	1.87
HLA-DRB3*01:01	9	21	13	KDYILDLQPETTD	1.87
HLA-DRB3*01:01	7	19	13	TIKDYILDLQPET	1.87
HLA-DQA1*05:01/DQB1*02:01	8	21	14	IKDYILDLQPETTD	1.94
HLA-DRB3*01:01	8	22	15	IKDYILDLQPETTDL	2.00
HLA-DRB3*01:01	9	23	15	KDYILDLQPETTDLH	2.00
HLA-DQA1*05:01/DQB1*02:01	7	21	15	TIKDYILDLQPETTD	2.00
HLA-DRB3*01:01	7	21	15	TIKDYILDLQPETTD	2.00
HLA-DQA1*05:01/DQB1*02:01	7	20	14	TIKDYILDLQPETT	2.05
HLA-DRB3*01:01	6	20	15	ATIKDYILDLQPETT	2.10
HLA-DRB3*01:01	5	19	15	KATIKDYILDLQPET	2.10
HLA-DQA1*03:01/DQB1*03:02	6	22	17	ATIKDYILDLQPETTDL	2.21

HLA-DQA1*05:01/DQB1*02:01	8	22	15	IKDYILDLQPETTDL	2.30
HLA-DQA1*03:01/DQB1*03:02	7	23	17	TIKDYILDLQPETTDLH	2.38
HLA-DQA1*05:01/DQB1*02:01	6	20	15	ATIKDYILDLQPETT	2.40
HLA-DQA1*05:01/DQB1*02:01	9	22	14	KDYILDLQPETTDL	2.48
HLA-DQA1*03:01/DQB1*03:02	8	24	17	IKDYILDLQPETTDLHC	2.55
HLA-DRB3*01:01	8	19	12	IKDYILDLQPET	2.58
HLA-DRB3*01:01	9	20	12	KDYILDLQPETT	2.67
HLA-DQA1*05:01/DQB1*02:01	6	21	16	ATIKDYILDLQPETTD	2.77
HLA-DQA1*05:01/DQB1*02:01	7	22	16	TIKDYILDLQPETTDL	2.77
HLA-DRB3*02:02	58	72	15	TDCYSCNSTLRLCIH	2.80
HLA-DQA1*05:01/DQB1*02:01	8	20	13	IKDYILDLQPETT	2.81
HLA-DRB3*02:02	57	71	15	VTDCYSCNSTLRLCI	2.90
HLA-DQA1*05:01/DQB1*02:01	9	21	13	KDYILDLQPETTD	2.96
HLA-DRB3*02:02	57	72	16	VTDCYSCNSTLRLCIH	3.00
HLA-DQA1*01:01/DQB1*05:01	5	18	14	KATIKDYILDLQPE	3.12
HLA-DQA1*03:01/DQB1*03:02	9	22	14	KDYILDLQPETTDL	3.12
HLA-DQA1*03:01/DQB1*03:02	10	23	14	DYILDLQPETTDLH	3.23
HLA-DRB3*01:01	8	23	16	IKDYILDLQPETTDLH	3.23
HLA-DRB3*02:02	56	71	16	IVTDCYSCNSTLRLCI	3.23
HLA-DRB3*02:02	58	73	16	TDCYSCNSTLRLCIHS	3.23
HLA-DRB3*01:01	7	22	16	TIKDYILDLQPETTDL	3.23
HLA-DQA1*03:01/DQB1*03:02	2	19	18	RGDKATIKDYILDLQPET	3.26
HLA-DQA1*05:01/DQB1*02:01	6	19	14	ATIKDYILDLQPET	3.34
HLA-DRB3*01:01	6	21	16	ATIKDYILDLQPETTD	3.35
HLA-DQA1*05:01/DQB1*02:01	5	20	16	KATIKDYILDLQPETT	3.35
HLA-DRB3*01:01	9	24	16	KDYILDLQPETTDLHC	3.35
HLA-DQA1*05:01/DQB1*02:01	9	23	15	KDYILDLQPETTDLH	3.40
HLA-DRB3*02:02	58	71	14	TDCYSCNSTLRLCI	3.45
HLA-DRB3*01:01	4	19	16	DKATIKDYILDLQPET	3.46
HLA-DRB3*01:01	10	25	16	DYILDLQPETTDLHCY	3.46
HLA-DRB3*01:01	5	20	16	KATIKDYILDLQPETT	3.46
HLA-DRB3*02:02	59	73	15	DCYSCNSTLRLCIHS	3.50
HLA-DQA1*03:01/DQB1*03:02	1	18	18	MRGDKATIKDYILDLQPE	3.55
HLA-DQA1*03:01/DQB1*03:02	10	25	16	DYILDLQPETTDLHCY	3.58
HLA-DQA1*03:01/DQB1*03:02	9	25	17	KDYILDLQPETTDLHCY	3.58
HLA-DQA1*03:01/DQB1*03:02	10	22	13	DYILDLQPETTDL	3.59
HLA-DQA1*03:01/DQB1*03:02	9	21	13	KDYILDLQPETTD	3.59
HLA-DQA1*01:01/DQB1*05:01	4	18	15	DKATIKDYILDLQPE	3.60
HLA-DRB3*02:02	59	72	14	DCYSCNSTLRLCIH	3.66
HLA-DQA1*05:01/DQB1*02:01	8	23	16	IKDYILDLQPETTDLH	3.69
HLA-DQA1*01:01/DQB1*05:01	5	19	15	KATIKDYILDLQPET	3.70
HLA-DPA1*03:01/DPB1*04:02	78	93	16	LRTLQQMLLGTLQVVC	3.81
HLA-DQA1*03:01/DQB1*03:02	4	21	18	DKATIKDYILDLQPETTD	3.85

HLA-DQA1*03:01/DQB1*03:02	3	20	18	GDKATIKDYILDLQPETT	3.85
HLA-DQA1*03:01/DQB1*03:02	9	23	15	KDYILDLQPETTDLH	3.90
HLA-DQA1*05:01/DQB1*02:01	7	19	13	TIKDYILDLQPET	3.90
HLA-DPA1*03:01/DPB1*04:02	77	92	16	DLRTLQQMLLGTLQVV	3.92
HLA-DQA1*03:01/DQB1*03:02	10	24	15	DYILDLQPETTDLHC	4.00
HLA-DQA1*01:01/DQB1*05:01	5	17	13	KATIKDYILDLQP	4.05
HLA-DQA1*01:01/DQB1*05:01	4	17	14	DKATIKDYILDLQP	4.09
HLA-DQA1*03:01/DQB1*03:02	10	21	12	DYILDLQPETTD	4.15
HLA-DQA1*03:01/DQB1*03:02	9	20	12	KDYILDLQPETT	4.15
HLA-DRB4*01:01	77	90	14	DLRTLQQMLLGTLQ	4.20
HLA-DQA1*05:01/DQB1*02:01	5	19	15	KATIKDYILDLQPET	4.20
HLA-DRB4*01:01	76	89	14	TDLRTLQQMLLGTL	4.20
HLA-DRB3*02:02	59	74	16	DCYSCNSTLRLCIHST	4.27
HLA-DPA1*03:01/DPB1*04:02	78	92	15	LRTLQQMLLGTLQVV	4.30
HLA-DQA1*01:01/DQB1*05:01	4	16	13	DKATIKDYILDLQ	4.37
HLA-DQA1*01:01/DQB1*05:01	3	16	14	GDKATIKDYILDLQ	4.41
HLA-DQA1*05:01/DQB1*02:01	6	22	17	ATIKDYILDLQPETTDL	4.43
HLA-DQA1*05:01/DQB1*02:01	5	21	17	KATIKDYILDLQPETTD	4.43
HLA-DQA1*03:01/DQB1*03:02	5	22	18	KATIKDYILDLQPETTDL	4.44
HLA-DRB3*02:02	60	74	15	CYSCNSTLRLCIHST	4.50
HLA-DRB3*02:02	60	73	14	CYSCNSTLRLCIHS	4.52
HLA-DPA1*03:01/DPB1*04:02	78	91	14	LRTLQQMLLGTLQV	4.52
HLA-DRB3*02:02	56	70	15	IVTDCYSCNSTLRLC	4.60
HLA-DQA1*01:02/DQB1*06:02	60	73	14	CYSCNSTLRLCIHS	4.74
HLA-DQA1*01:02/DQB1*06:02	59	72	14	DCYSCNSTLRLCIH	4.74
HLA-DQA1*05:01/DQB1*02:01	9	20	12	KDYILDLQPETT	4.74
HLA-DQA1*01:02/DQB1*06:02	62	75	14	SCNSTLRLCIHSTA	4.74
HLA-DQA1*01:02/DQB1*06:02	61	74	14	YSCNSTLRLCIHST	4.74
HLA-DRB3*02:02	56	72	17	IVTDCYSCNSTLRLCIH	4.77
HLA-DRB3*02:02	57	73	17	VTDCYSCNSTLRLCIHS	4.77
HLA-DQA1*01:01/DQB1*05:01	3	17	15	GDKATIKDYILDLQP	4.80
HLA-DRB4*01:01	77	89	13	DLRTLQQMLLGTL	4.83
HLA-DQA1*05:01/DQB1*02:01	9	24	16	KDYILDLQPETTDLHC	4.85
HLA-DRB3*02:02	55	70	16	YIVTDCYSCNSTLRLC	4.85
HLA-DQA1*01:01/DQB1*05:01	6	19	14	ATIKDYILDLQPET	4.95
HLA-DRB4*01:01	76	90	15	TDLRTLQQMLLGTLQ	5.00

Note: A low adjusted rank (AR) showed a good binder，and only epitopes with AR≤5 were selected.
