# Supplementary material for: The genetic variability, phylogeny and functional significance of E6, E7 and LCR in human papillomavirus type 52 isolates in Sichuan, China
Source: Virol J. 2021 May 3;18:94. doi: 10.1186/s12985-021-01565-5 (PMC8091156; doi:10.1186/s12985-021-01565-5)
Supplement: Supplementary file 6 — Additional file 6: Table S11. Predicted linear B cell epitopes of HPV-52 E6. Table S12. Predicted linear B cell epitopes of HPV-52 E7. [file 12985_2021_1565_MOESM6_ESM.pdf]

Table S11 Predicted linear B cell epitopes of HPV-52 E6

reference				variant			
Rank	Sequence	Start position	Score	Rank	Sequence	Start position	Score
1	MGRWTGRCSECWRPRP	129	0.91	1	MGRWTGRCSECWRPRP	129	0.91
1	TPLCPEEKERHVNANK	108	0.91	1	TPLCPEEKERHVNANK	108	0.91
2	YGVCIMCLRFLSKISE	60	0.9	2	EERVRKPLSEITIRCI	89	0.9
3	EERVKKPLSEITIRCI	89	0.87	2	YGVCIMCLRFLSKISE	60	0.9
4	DLRIVYRDNNPYGVCI	49	0.83	3	YSLYGKTLEERVRKPL	81	0.84
5	YSLYGKTLEERVKKPL	81	0.81	4	DLRIVYRDNNPYGVCI	49	0.83
6	CEVLEESVHEIRLQCV	16	0.79	5	CEVLEESVHEIRLQCV	16	0.79
6	ERHVNANKRFHNIMGR	116	0.79	5	ERHVNANKRFHNIMGR	116	0.79
7	KKELQRREVYKFLFTD	34	0.74	6	KKELQRREVYKFLFTD	34	0.74
8	ISEYRHYQYSLYGKTL	73	0.73	6	QDPATRPRTLHELCEV	3	0.74
9	EITIRCIICQTPLCPE	98	0.72	7	ISEYRHYQYSLYGKTL	73	0.73
10	EDPATRPRTLHELCEV	3	0.68	8	EITIRCIICQTPLCPE	98	0.72
11	VHEIRLQCVQCKKELQ	23	0.65	9	VHEIRLQCVQCKKELQ	23	0.65

Table S12 Predicted linear B cell epitopes of HPV-52 E7

reference				variant			
Rank	Sequence	Start position	Score	Rank	Sequence	Start position	Score
1	HCYEQLGDSSDEEDTD	23	0.9	1	EEDIDGVDRPDGQAEQ	34	0.9
2	IVTYCHSCDSTLRLCI	56	0.86	2	DGQAEQATDNYYIVTD	44	0.85
3	ATSNYYIVTYCHSCDS	50	0.85	3	EQLGDSSDEEDIDGVD	26	0.84
3	DTDGVDRPDGQAEQAT	36	0.85	4	ATDNYYIVTDCYSCNS	50	0.76
4	GDSSDEEDTDGVDRPD	29	0.78	5	KATIKDYILDLQPETT	5	0.75
5	KATIKDYILDLQPETT	5	0.75	6	CIHSTATDLRTLQQML	70	0.71
6	CIHSTATDLRTLQQML	70	0.71	6	PETTDLHCYEQLGDSS	17	0.71
6	PETTDLHCYEQLGDSS	17	0.71	7	RTLQQMLLGTLQVVCP	79	0.67
7	SCDSTLRLCIHSTATD	62	0.69	8	IVTDCYSCNSTLRLCI	56	0.64
8	RTLQQMLLGTLQVVCP	79	0.67	9	SCNSTLRLCIHSTATD	62	0.59
